# CMTM6 drives cisplatin resistance by regulating Wnt signaling through the ENO-1/AKT/GSK3**β** axis

**DOI:** 10.1172/jci.insight.143643

**Published:** 2021-02-22

**Authors:** Pallavi Mohapatra, Omprakash Shriwas, Sibasish Mohanty, Arup Ghosh, Shuchi Smita, Sandeep Rai Kaushik, Rakesh Arya, Rachna Rath, Saroj Kumar Das Majumdar, Dillip Kumar Muduly, Sunil K. Raghav, Ranjan K. Nanda, Rupesh Dash

**Affiliations:** 1 Institute of Life Sciences, Bhubaneswar, India.; 2 Regional Centre for Biotechnology, Faridabad, India.; 3 Manipal Academy of Higher Education, Manipal, India.; 4 Translational Health Group, International Centre for Genetic Engineering and Biotechnology, New Delhi, India.; 5 Sriram Chandra Bhanj Medical College and Hospital, Cuttack, India.; 6 Department of Radiotherapy and; 7 Department of Surgical Oncology, All India Institute of Medical Sciences, Bhubaneswar, India.

**Keywords:** Cell Biology, Oncology, Head and neck cancer, Molecular biology, Signal transduction

## Abstract

Rewiring tumor cells to undergo drug-induced apoptosis is a promising way to overcome chemoresistance. Therefore, identifying causative factors for chemoresistance is of high importance. Unbiased global proteome profiling of sensitive, early, and late cisplatin-resistant oral squamous cell carcinoma (OSCC) lines identified CMTM6 as a top-ranked upregulated protein. Analyses of OSCC patient tumor samples demonstrated significantly higher CMTM6 expression in chemotherapy (CT) nonresponders as compared with CT responders. In addition, a significant association between higher CMTM6 expression and poorer relapse-free survival in esophageal squamous cell carcinoma, head and neck squamous cell carcinoma, and lung squamous cell carcinoma was observed from Kaplan-Meier plot analysis. Stable knockdown (KD) of CMTM6 restored cisplatin-mediated cell death in chemoresistant OSCC lines. Upon CMTM6 overexpression in CMTM6-KD lines, the cisplatin-resistant phenotype was rescued. The patient-derived cell xenograft model of chemoresistant OSCC displaying CMTM6 depletion restored the cisplatin-induced cell death and tumor burden substantially. The transcriptome analysis of CMTM6-KD and control chemoresistant cells depicted enrichment of the Wnt signaling pathway. We demonstrated that CMTM6 interaction with membrane-bound Enolase-1 stabilized its expression, leading to activation of Wnt signaling mediated by AKT–glycogen synthase kinase-3β. CMTM6 has been identified as a stabilizer of programmed cell death ligand 1. Therefore, as CMTM6 facilitates tumor cells for immune evasion and mediates cisplatin resistance, it could be a promising therapeutic target for treating therapy-resistant OSCC.

## Graphical abstract

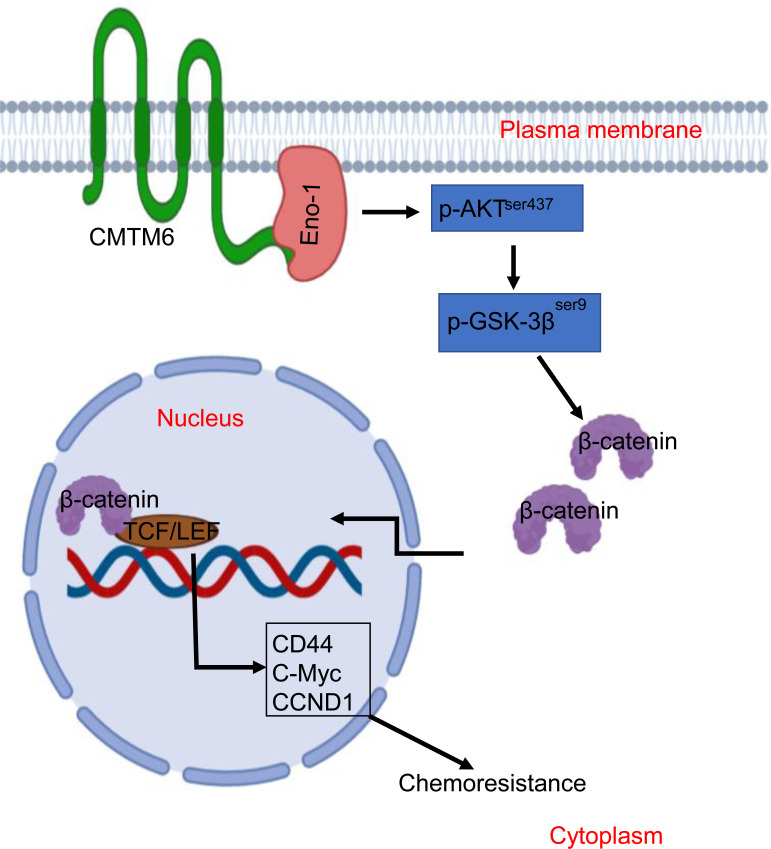

## Introduction

Head and neck squamous cell carcinoma (HNSCC) is the sixth most common cancer worldwide, with approximately 53,260 new cases reported annually in the United States alone (1). Almost 90% of HNSCC cancer cases are oral squamous cell carcinoma (OSCC), which has emerged as the most common cancer in developing countries. In India, every year 80,000 new OSCC cases are reported with a mortality of approximately 46,000 (2). Most patients with OSCC present with locally advanced (stage III or IV) disease. The treatment modalities of advanced OSCC are surgical removal of primary tumor followed by chemotherapy (CT) and radiotherapy (3). However, neoadjuvant CT is commonly prescribed for surgically unresectable OSCC tumors (4). Despite the availability of treatment modalities, the 5-year survival rate of patients with advanced tongue OSCC remains less than 50%, indicating therapy resistance.

Chemoresistance is one of the major factors for treatment failure in OSCC. The common CT regimens for OSCC are cisplatin alone or cisplatin, 5-fluorouracil (5FU), and docetaxel (TPF) (5). The tumor shows initial positive response to CT, but later it acquires chemoresistance and patients experience relapse with onset of metastatic diseases. The chemoresistant properties could be attributed to enhanced cancer stem cell (CSC) population, decreased drug accumulation, reduced drug-target interaction, reduced apoptotic response, and enhanced autophagy (6). These hallmarks present the endpoint events, when cancer cells had already acquired chemoresistance. Few attempts have been made to understand the molecular mechanism of chemoresistance in HNSCC. In 2012, Peng and colleagues demonstrated that tongue cancer CT resistance-associated protein 1 (TCRP1) is a modulator of cisplatin resistance in OSCC. TCRP1 expression is elevated specifically in cisplatin-resistant cells but not in 5FU-resistant cancer cells. Analysis of clinical samples indicated that TCRP1-positive OSCC patients are resistant to cisplatin (7). Similarly, a shRNA-based human kinome study elucidated that microtubule-associated serine/threonine kinase 1 (MAST1) is a major driver of cisplatin resistance in HNSCC. MAST1 inhibitor lestaurtinib efficiently sensitized chemoresistant cells to cisplatin. Overall, the study suggests that MAST1 is a viable target to overcome cisplatin resistance (8).

CMTM6 represents the CMTM family member proteins, a family consisting of 8 members (9). The CMTM6 gene is located on chromosome 3p22 (10), and all the CMTM proteins belong to a chemokine-like factor gene superfamily, similar to the chemokine and transmembrane 4 superfamilies (9). CMTM6 is a type 3 transmembrane protein with a MARVEL domain consisting of 4 transmembrane helices. It is well established that MARVEL domain proteins play an important role in regulating the trafficking of transmembrane proteins (11). The subcellular localization of CMTM6 is mostly in plasma membrane and expressed in several tissues of the human body (The Human Protein Atlas, https://www.proteinatlas.org/ENSG00000091317-CMTM6/tissue). Until 2017, very little was known about the function of this novel protein. A genome-wide CRISPR-based screening in pancreatic cancer cell line identified that CMTM6 stabilizes programmed cell death ligand 1 (PD-L1) expression. Interestingly, CMTM6 interacts and colocalizes with PD-L1 in plasma membrane. Again, CMTM6 prevents the lysosome-mediated degradation of PD-L1, therefore, it stabilizes PD-L1 expression (12, 13). Overall, these studies suggest that CMTM6 is an important factor for immune invasion by tumor cells. Additional studies suggest that knocking down CMTM6 results in decreased PD-L1 expression and increased infiltration of CD8^+^ and CD4^+^ T cells, that in turn increased the antitumor immunity in HNSCC (14). CMTM6 expression is also upregulated in high-grade malignant glioma, and it can be correlated with poor prognosis of glioma patients (15). In lung cancer patients, CMTM6 acts as a predictor for programmed cell death 1 (PD-1) inhibitor therapy; i.e., patients with higher CMTM6 expression respond well to PD-1 inhibitors (16, 17). Therefore, because CMTM6 expression has been detected in several tissues, it is predicted that it might have several biological functions other than triggering immune evasion by tumor cells.

Here, to elucidate the causative factors responsible for acquired chemoresistance, we have performed global proteomic profiling of sensitive, early, and late cisplatin-resistant OSCC lines. The top-ranked upregulated protein CMTM6 was selected for validation in multiple cell lines and patient-derived biopsy samples. shRNA-based depletion of CMTM6 in cisplatin-resistant cells and in vivo patient-derived cell (PDC1) xenograft models restored drug-induced apoptosis as evident from substantial reduction of tumor burden. Ultimately, we demonstrated the interaction of CMTM6 with membrane-bound Enolase-1 regulates Wnt signaling through the AKT/GSK3β/β-catenin axis, augmenting the chemoresistant phenotype.

## Results

### Generation and characterization of early and late cisplatin-resistant cell lines.

First, we evaluated the cisplatin-induced cell death in a sensitive, early, and late cisplatin-resistant pattern (CisS, CisR4M, and CisR8M) of H357, SCC9, and SCC4 cells by performing MTT (cell viability) assay. We observed complete acquired resistance in CisR8M and partial resistance in CisR4M cells (Supplemental Figure 1, A and B; supplemental material available online with this article; https://doi.org/10.1172/jci.insight.143643DS1). In addition, we established a CisS and CisR8M pattern of A549 (lung carcinoma) and A375 (melanoma) cells (Supplemental Figure 1C).

### Global proteomic profiling of cisplatin-resistant cell lines identified CMTM6 as a potential candidate.

**Figure 1:**
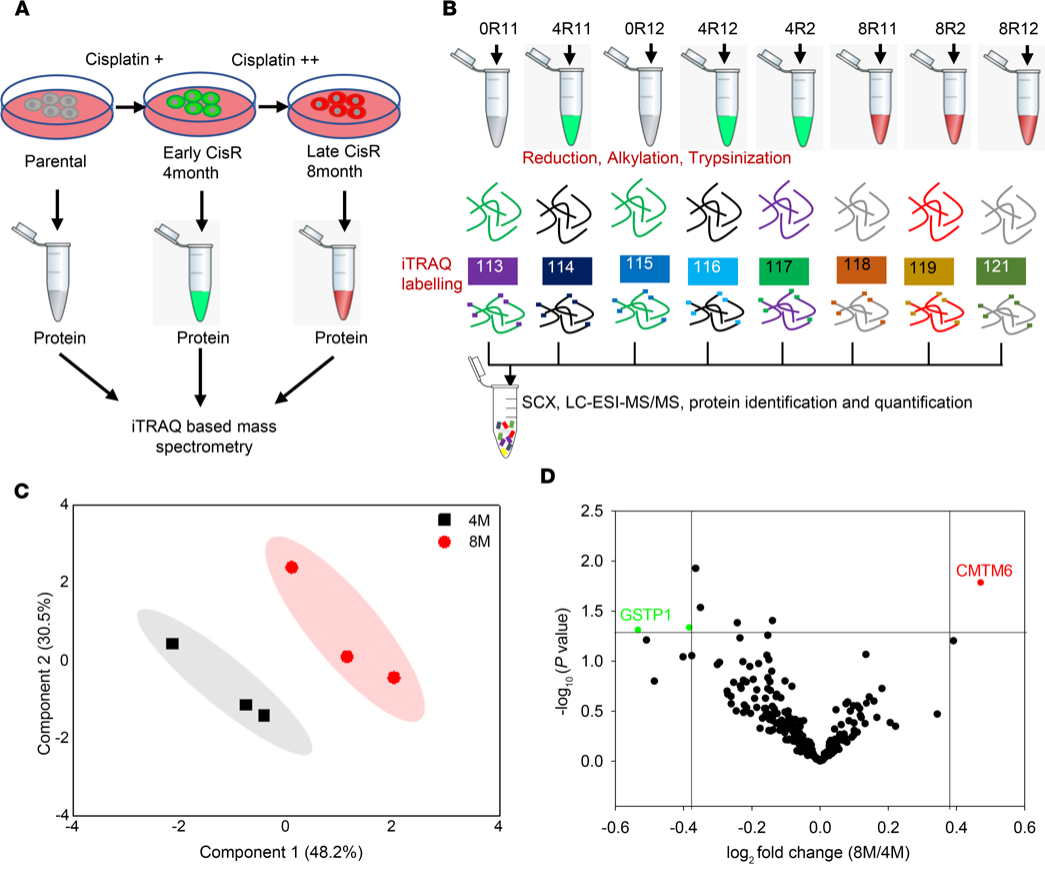
iTRAQ-based proteomic analysis. (**A**) Schematic representation of sensitive, early, and late cisplatin-resistant OSCC line for global proteomic profiling. The establishment of sensitive, early, and late resistant cells is described in Methods. (**B**) The lysates were isolated from parental sensitive (H357CisS), early (H357CisR4M), and late (H357CisR8M) cisplatin-resistant cells and subjected to global proteomic profiling. The schematic diagram depicts the iTRAQ labeling strategy for proteomic analysis. 0R11 and 0R12 are biological replicates of the H357CisS group, 4R11: 4R12 and 4R2 are technical and biological replicates of the H357CisR4M group, and 8R11: 8R12 and 8R2 are technical and biological replicates of H357CisR8M group. (**C**) Principal component analysis (PCA) of global proteomic profiling sensitive, early (4M), and late resistant cells (8M). (**D**) Volcano plot indicating deregulated genes in proteome profiling of sensitive and cisplatin-resistant cells. CMTM6 is the top-ranked upregulated genes in 4M and 8M cisplatin-resistant groups. SCX, strong cation exchange; and LC-ESI, liquid chromatography electrospray ionization.

To explore the causative factors responsible for acquired cisplatin resistance in OSCC, we performed an unbiased and global proteomic profiling of H357CisS, H357CisR4M, and H357CisR8M cells. We identified 367 proteins to be differentially regulated in CisR4M and CisR8M as compared with cisplatin sensitive control cells (Supplemental Table 3). Principal component analysis (PCA) using all identified proteins with their differential fold change values grouped them into 2 isolated clusters (Figure 1, A–C). The dendrogram and volcano plot show CMTM6 as the top-ranked upregulated protein in late cisplatin-resistant cells compared with early resistant cells and their sensitive counterpart (Figure 1D and Supplemental Figure 2). Based on these findings, CMTM6 was selected for further experimental validation and to explore its potential role in driving cisplatin resistance.

### CMTM6 expression is elevated and positively correlated with PD-L1 in cisplatin-resistant cancers.

**Figure 2:**
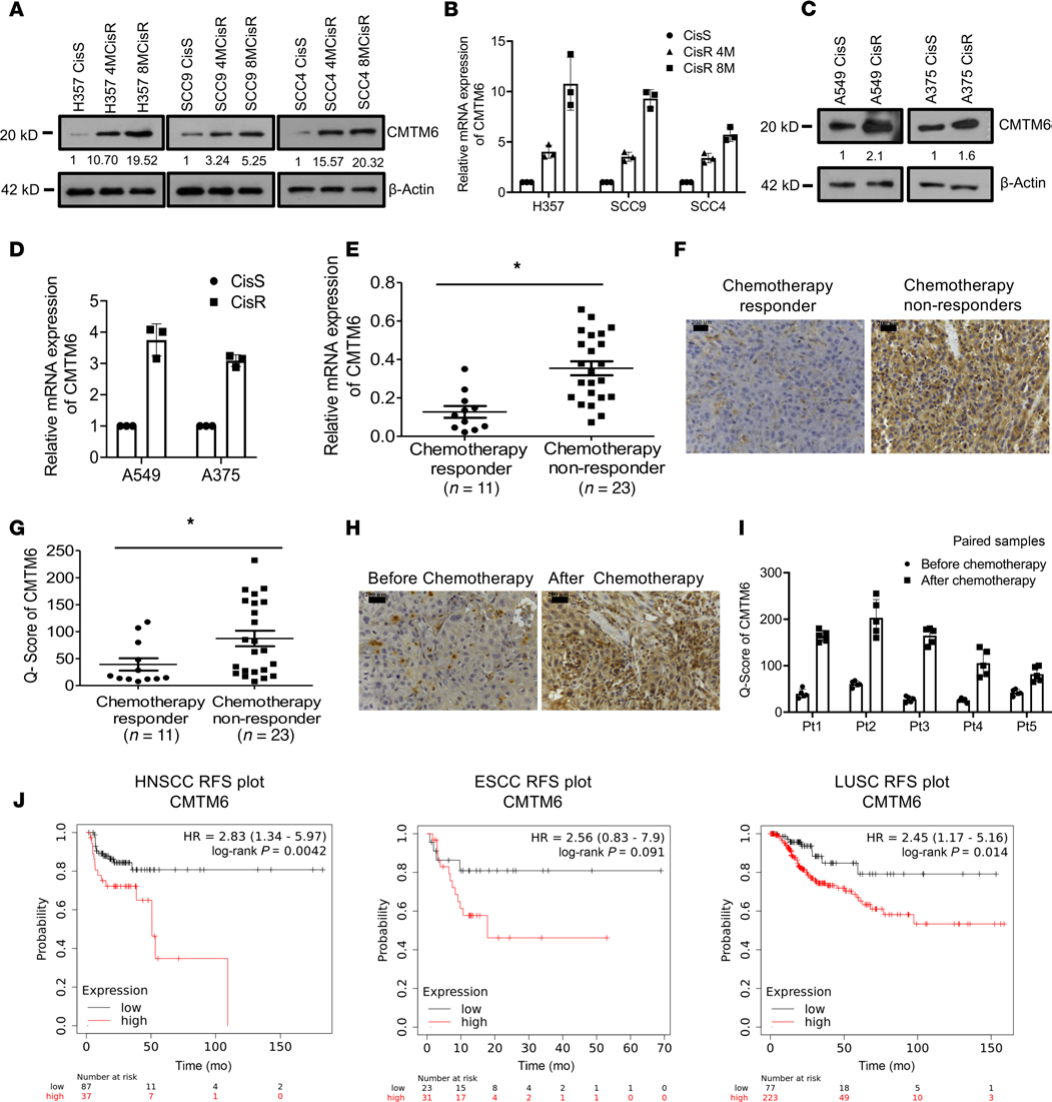
CMTM6 is upregulated in chemoresistant squamous cell carcinomas. (**A**) Cell lysates from indicated resistant and sensitive OSCC cells were isolated and subjected to immunoblotting (*n* = 3) against CMTM6 and β-actin antibodies. (**B**) Relative mRNA (fold change) CMTM6 expression was analyzed by quantitative real-time PCR (qRT-PCR) in indicated cells (mean ± SEM, *n* = 3), *P < 0.05 by 1-way ANOVA. (**C**) Cell lysates from indicated resistant and sensitive OSCC cells were isolated and subjected to immunoblotting (*n* = 3) against CMTM6 and β-actin antibodies. (**D**) Relative mRNA (fold change) CMTM6 expression was analyzed by qRT-PCR in indicated cells (mean ± SEM, *n* = 3), *P < 0.05 by 1-way ANOVA. (**E**) Relative mRNA expression of CMTM6 was analyzed by qRT-PCR in different chemotherapy nonresponder (CT nonresponder) OSCC tumors as compared with CT responder tumors (median, *n* = 11 for CT responder and *n* = 23 for CT nonresponder). *P < 0.05 by 2-tailed Student’s *t* test. (**F**) Protein expression of CMTM6 was analyzed by IHC in CT responder and CT nonresponder OSCC tumors. Scale bars: 50 μm. (**G**) IHC scoring for CMTM6 from **J** (Q score = staining intensity × percent of staining) (median, *n* = 11 for CT responder and *n* = 23 for CT nonresponder). *P < 0.05 by 2-tailed Student’s *t* test. (**H**) CMTM6 protein expression was analyzed by IHC in pre– and post–TPF-treated paired tumor samples for CT nonresponder patients. Scale bars: 50 μm. (**I**) IHC scoring for CMTM6 from **H** (Q score = staining intensity × percent of IHC staining). (**J**) Relapse-free survival (RFS) plot for CMTM6 using Kaplain-Meier Plotter.

To investigate CMTM6 expression and its association with cisplatin resistance in human cancers, CMTM6 expression was examined in cisplatin-sensitive and -resistant human cancer cell lines and in tumor tissues of OSCC patients. CMTM6 expression was evaluated in H357, SCC9, and SCC4 cell lines showing early and late chemoresistant patterns (H357CisR4M, H357CisR8M, SCC4CisR4M, SCC4CisR8M, SCC9CisR4M, and SCC9CisR8M). CMTM6 expression at protein as well as mRNA levels were upregulated in CisR4M and CisR8M cells with respect to CisS counterparts in all cell lines (Figure 2, A and B). Also, elevated CMTM6 expression was observed in A549CisR and A375CisR cells as compared with the sensitive cells (Figure 2, C and D). To evaluate the clinical significance of this observation, we monitored CMTM6 expression in tumors isolated from neoadjuvant-treated CT responder and CT nonresponder OSCC patients. Based on real-time quantitative PCR (qRT-PCR) and IHC analysis, higher abundance of CMTM6 was observed in tumor tissues of nonresponders as compared with responder OSCC patients (Figure 2, E–G). Similarly, we determined the CMTM6 expression in drug-naive and post-CT nonresponder paired tumor samples from the same patient. We found that the post–CT-treated tumor samples showed higher CMTM6 expression (Figure 2, H and I). CMTM6 has been reported as an important regulator of PD-L1 expression (12). PD-L1 expression was found to be upregulated in both chemoresistant cancer lines as well as in CT nonresponder patients, compared with sensitive counterparts (Supplemental Figure 3, A–E). CMTM6 and PD-L1 expression also showed a positive correlation in CT nonresponders (Supplemental Figure 3F). Further, the Kaplan-Meier plotter (KM Plotter) tool also depicted a significant association of CMTM6 as well as PD-L1 expression and poor relapse-free survival in ESCC, HNSCC, and lung squamous cell carcinoma (Figure 2J and Supplemental Figure 3G). Overall, it was found that CMTM6 expression is significantly elevated in chemoresistant lines, which is positively correlated with PD-L1 expression level.

### CMTM6 depletion reverses cisplatin resistance phenotype.

**Figure 3:**
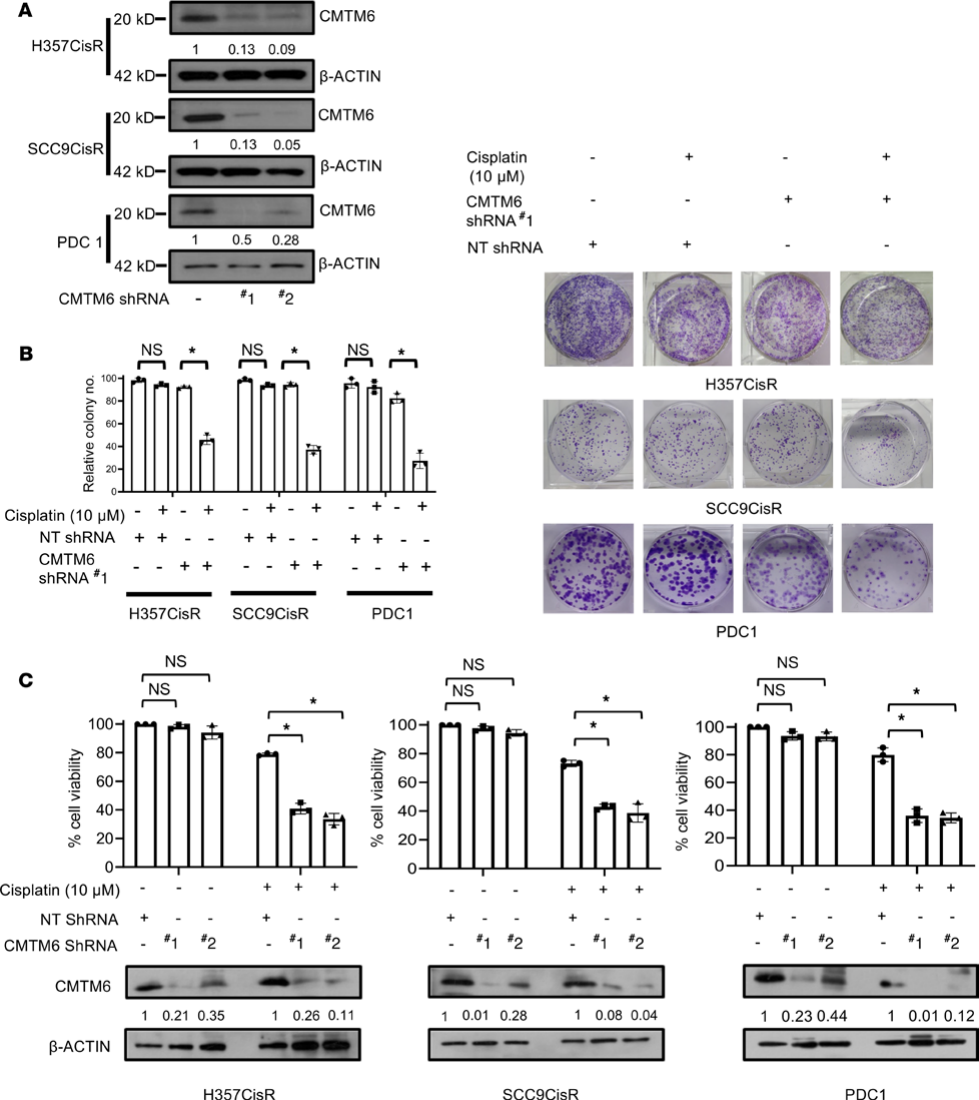
Lentivirus-mediated stable KD of CMTM6 reduces cell growth and proliferation of cisplatin-resistant OSCC. (**A**) Cisplatin-resistant OSCC lines were stably transfected with NTShRNA and CMTM6ShRNA as described in Methods. ShRNA#1 targets CMTM6 mRNA and ShRNA#2 targets 5′UTR of CMTM6 mRNA. Lysates were collected from indicated stable clone, and immunoblotting (*n* = 3) was performed with anti-CMTM6 and β-actin antibodies. (**B**) Cisplatin-resistant cells stably expressing NTShRNA and CMTM6ShRNA were treated with cisplatin for 12 days and colony-forming assays performed as described in Methods (left). Relative colony number (*n* = 3, *P < 0.05 by 2-way ANOVA). Photographs of colony-forming assay in each group (right). (**C**) Cisplatin-resistant cells stably expressing NTShRNA and CMTM6ShRNA were treated with cisplatin for 48 hours, and cell viability was determined by MTT assay (*n* = 3), *P < 0.05 by 2-way ANOVA.

**Figure 4:**
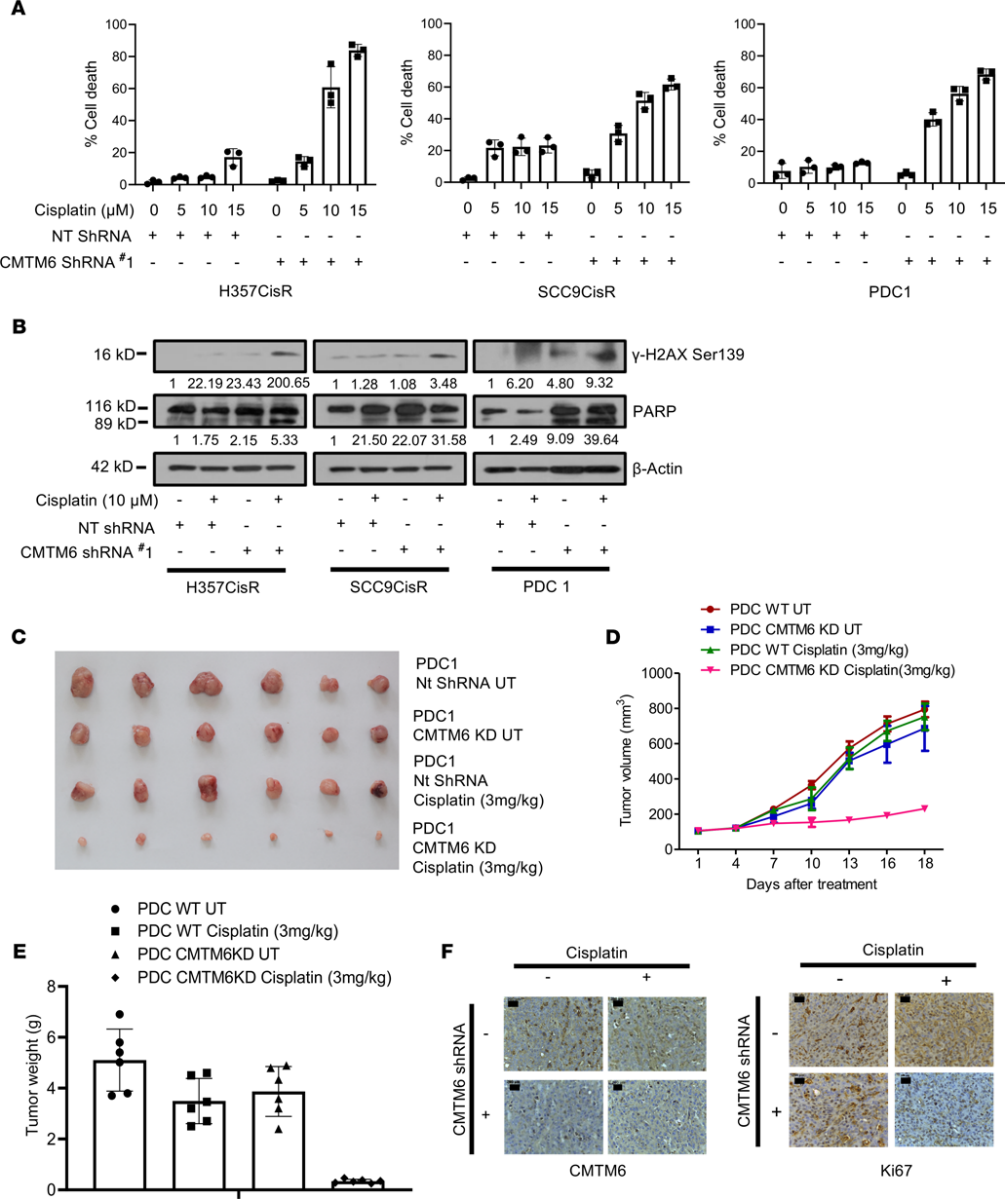
CMTM6 KD restores cisplatin-induced cell death in drug-resistant OSCC. (**A**) Cisplatin-resistant cells stably expressing NTShRNA and CMTM6ShRNA were treated with cisplatin for 48 hours, after which cell death was determined by annexin V/7AAD assay using flow cytometer. Bar diagrams indicate the percentage of cell death with respective treated groups (mean ± SEM, *n* = 3). Two-way ANOVA. (**B**) Cisplatin-resistant cells stably expressing NTShRNA and CMTM6ShRNA were treated with cisplatin for 48 hours, and immunoblotting (*n* = 3) was performed with indicated antibodies. (**C**) Patient-derived cells (PDC1) established from tumor of CT nonresponder patient. PDC1 cells stably expressing NtShRNA were implanted in the right upper flank of athymic male nude mice, and PDC1 cells stably expressing CMTM6ShRNA#1 (PDC1 CMTM6KD) were implanted in the left upper flank, after which they were treated with cisplatin at indicated concentration. At the end of the experiment mice were euthanized, and tumors were isolated and photographed (*n* = 6). (**D**) Tumor growth was measured at the indicated time point using digital slide calipers and plotted as a graph (mean ± SEM, *n* = 6). Two-way ANOVA. (**E**) Bar diagram indicates the tumor weight measured at the end of the experiment (mean ± SEM, *n* = 6). Two-way ANOVA. (**F**) After completion of treatment, tumors were isolated, and paraffin-embedded sections were prepared as described in Methods to perform IHC with indicated antibodies. Scale bars: 50 μm.

To delineate the potential role of CMTM6 as a major driver of cisplatin resistance, stable CMTM6-knockdown (CMTM6-KD) cell clones were generated in chemoresistant lines using a lentivirus-based shRNA approach. To knock down CMTM6, we used 2 shRNAs, one targeting the coding sequence (CMTM6 shRNA#1) and the other targeting 5′UTR (CMTM6 shRNA#2) of CMTM6 mRNA. Stable clones generated by both shRNAs showed efficient depletion of CMTM6 (Figure 3A). Cell viability and cell death assay data suggested that CMTM6KD significantly sensitized the chemoresistant cells to cisplatin treatment (Figure 3, B and C, and Figure 4A). Similarly, after knocking down CMTM6 in patient-derived primary tumor cells not responding to taxol and platinum therapy (TP), PDC1 cells reversed resistance and became sensitive to cisplatin (Figure 3, B and C, and Figure 4A). Immunoblotting data showed that knocking down CMTM6 in chemoresistant cells substantially increased cisplatin-induced cleaved poly (ADP-ribose) polymerase (PARP) and γ-H2AX expression (Figure 4B). To evaluate the in vivo efficacy of CMTM6 KD in reversing chemoresistance, we generated PDC1-based (patient 1 from the CT nonresponder group: Supplemental Table 1) based xenografts in nude mice. Here, we implanted control shRNA–transduced PDC1 cells in the right upper flank and CMTM6 shRNA–transduced PDC1 cells in the left upper flank of the same mice. Treatment with cisplatin (3 mg/kg) markedly reduced the tumor burden in the CMTM6 shRNA group but not in the control shRNA–treated (NTsh-treated) group (Figure 4, C–E). We also observed markedly decreased cell proliferation in cisplatin-treated CMTM6 shRNA–treated tumors (Figure 4F). These data suggest CMTM6 dependency of chemoresistant OSCC cells.

### Exogenous CMTM6 expression reversed chemoresistant phenotype in KD cells.

**Figure 5:**
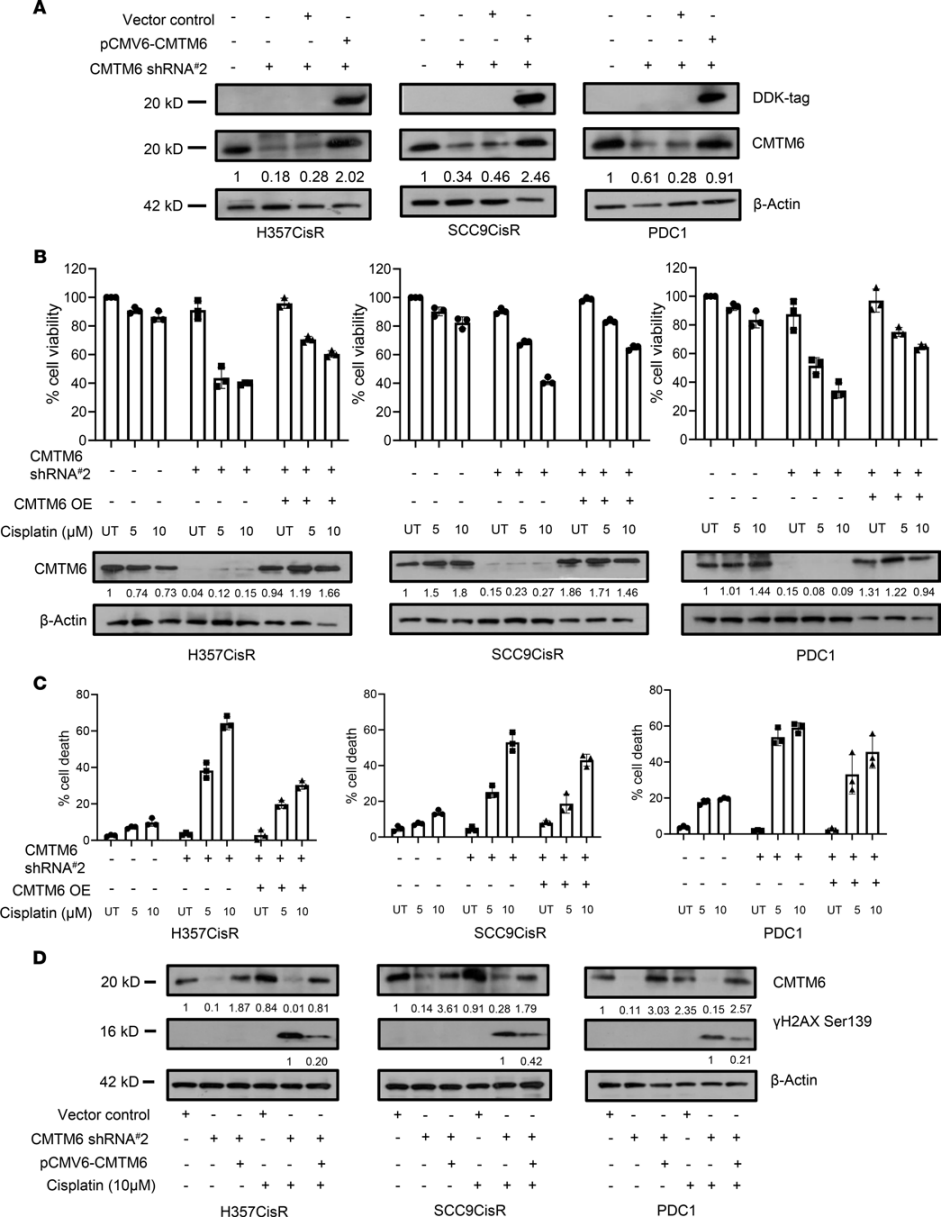
Ectopic CMTM6 overexpression rescued the drug-resistant phenotype in CMTM6-KD cells. (**A**) For ectopic overexpression, pCMV6-Entry-CMTM6 (MYC-DDK tagged) and control vector were transiently transfected to indicated CMTMKD (ShRNA#2) cells, and immunoblotting (*n* = 3) was performed with indicated antibodies. Efficient overexpression was evident from CMTM6 and DDK expression. (**B**) CMTM6 was overexpressed in chemoresistant cells stably expressing CMTM6ShRNA#2 and treated with cisplatin with indicated concentration for 48 hours, after which cell viability was determined by MTT assay (*n* = 3), 2-way ANOVA. (**C**) Cells were treated as indicated in **B**, and cell death was determined by annexin V/7AAD assay using flow cytometer. Bar diagrams indicate the percentage of cell death with respective treated groups (mean ± SEM, *n* = 3), 2-way ANOVA. (**D**) CMTM6 was overexpressed in chemoresistant cells stably expressing CMTM6ShRNA#2 followed by cisplatin treatment for 48 hours, and immunoblotting (*n* = 3) was performed with indicated antibodies.

To confirm the potential role of CMTM6 in modulating cisplatin resistance, CMTM6 was transiently overexpressed in chemoresistant cells that were stably transduced with CMTM6 shRNA#2 that targets 5′UTR of the CMTM6 gene (Figure 5A). The MTT assay and annexin-V/7AAD staining data depicted that ectopic overexpression of CMTM6 in CMTM6-KD drug-resistant cells resulted in reversal of the cisplatin-resistant phenotype (Figure 5, B and C). Similarly, transient overexpression of CMTM6 decreased γ-H2AX expression in CMTM6-depleted cells (Figure 5D). Furthermore, we demonstrated that CMTM6 overexpression also contributed to cisplatin-resistant phenotype in cisplatin-sensitive H357 and SCC9 lines (Supplemental Figure 4, A and B). These findings further support our hypothesis that CMTM6 regulates cisplatin resistance.

### CMTM6 regulates AKT/glycogen synthase kinase-3β signaling through membrane Enolase-1.

**Figure 6:**
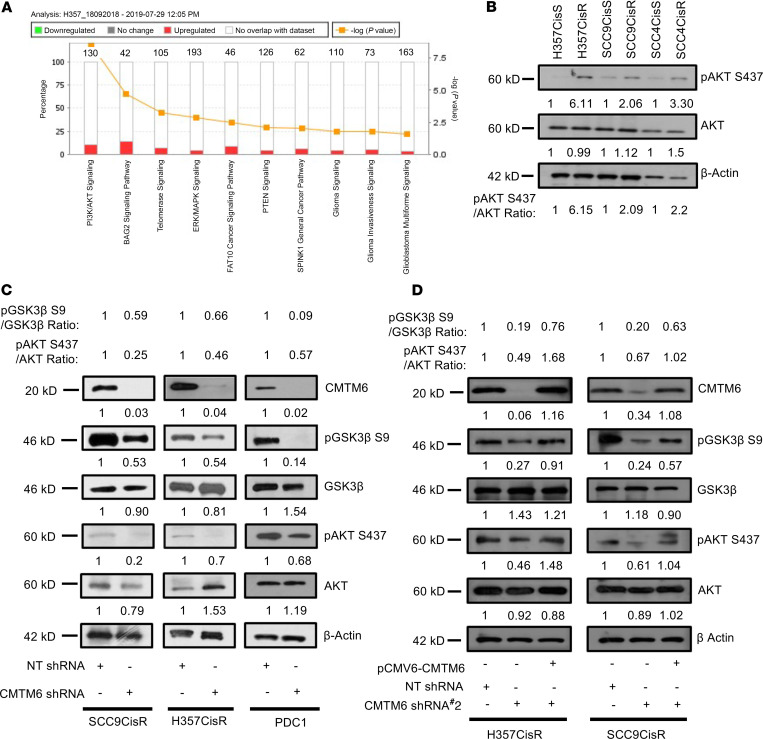
CMTM6 activates AKT/GSK3β signaling. (**A**) Deregulated genes from proteomic profiling were subjected to Ingenuity Pathway Analysis, and pathways involved are indicated as a bar diagram. (**B**) Cell lysates were prepared from indicated sensitive and resistant cancer lines and subjected to immunoblotting (*n* = 3) with specified antibodies. (**C**) Lysates were isolated and subjected to immunoblotting (*n* = 3) with indicated antibodies in chemoresistant cells stably expression NTShRNA or CMTM6ShRNA#1. (**D**) CMTM6 was overexpressed in chemoresistant cells stably expressing NTShRNA or CMTM6ShRNA#2, and immunoblotting (*n* = 3) was performed with indicated antibodies.

**Figure 7:**
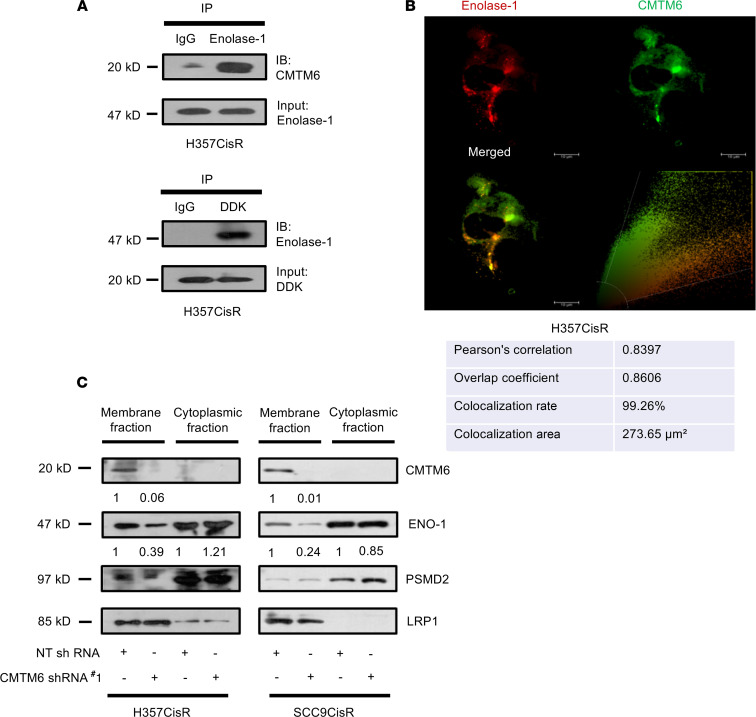
CMTM6 stabilizes membrane Enolase-1 expression. (**A**) Lysates were isolated from H357CiSR and immunoprecipitated with Enolase-1 and immunoblotting was performed with CMTM6. H357CisR was transfected with pCMV6-Entry-CMTM6 (MYC-DDK tagged), after which the lysates were isolated and IP followed by IB was performed with indicated antibodies. (**B**) H357CisR cells were subjected to immunostaining with anti-CMTM6 and anti–Enolase-1 using confocal microscope as described in Methods. (**C**) Lysates were isolated from membrane and cytoplasmic fraction, and immunoblotting (*n* = 3) was performed using indicated antibodies.

To explore the molecular mechanisms underlying cisplatin resistance, the list of differentially regulated proteins from global proteomic analysis was generated through the use of Ingenuity Pathway Analysis (IPA). Multiple functional pathways were found to be regulated in acquired CT-resistant cells with significant enrichment of PI3K/AKT signaling (Figure 6A), which was further validated by immunoblotting for AKT and phosphorylation level of AKT (p-AKT S437) (Figure 6B). To investigate whether CMTM6 has any role to play in the regulation of PI3K/AKT signaling, we evaluated expression of AKT, p-AKT (S437), and its downstream glycogen synthase kinase (GSK) and inhibitory phosphorylation p-GSK3β S9 in CMTM6-depleted and control vector–treated chemoresistant cells. The immunoblotting data show that KD of CMTM6 in chemoresistant cells resulted in substantial reduction of p-AKT and p-GSK3β expression (Figure 6C). On the contrary, CMTM6 overexpression in CMTM6-KD chemoresistant cells reversed p-AKT (S437) and p-GSK3β (S9) expression (Figure 6D). Earlier, it was reported that Enolase-1 can activate AKT signaling (18). Further, mass spectrometry–based analysis by Burr and colleagues (12) showed that CMTM6 interacts with Enolase-1. Therefore, we confirmed that CMTM6 interacted with Enolase-1 by coimmunoprecipitation and confocal microscopy (Figure 7, A and B). Additionally, we analyzed Enolase-1 expression in cytoplasmic and membrane fractions in CMTM6-KD and control vector–transduced chemoresistant cells. We found substantial reduction of membrane Enolase-1 in CMTM6-KD cells (Figure 7C). Again, cell viability and cell death assay showed that ENO-1 KD significantly sensitized chemoresistant lines to cisplatin treatment (Supplemental Figure 5, A and B). These data strongly demonstrated the role of the CMTM6/Enolase-1/AKT signaling axis in regulation of cisplatin resistance.

### Transcriptome analysis revealed CMTM6 as a potential modulator of WNT signaling.

**Figure 8:**
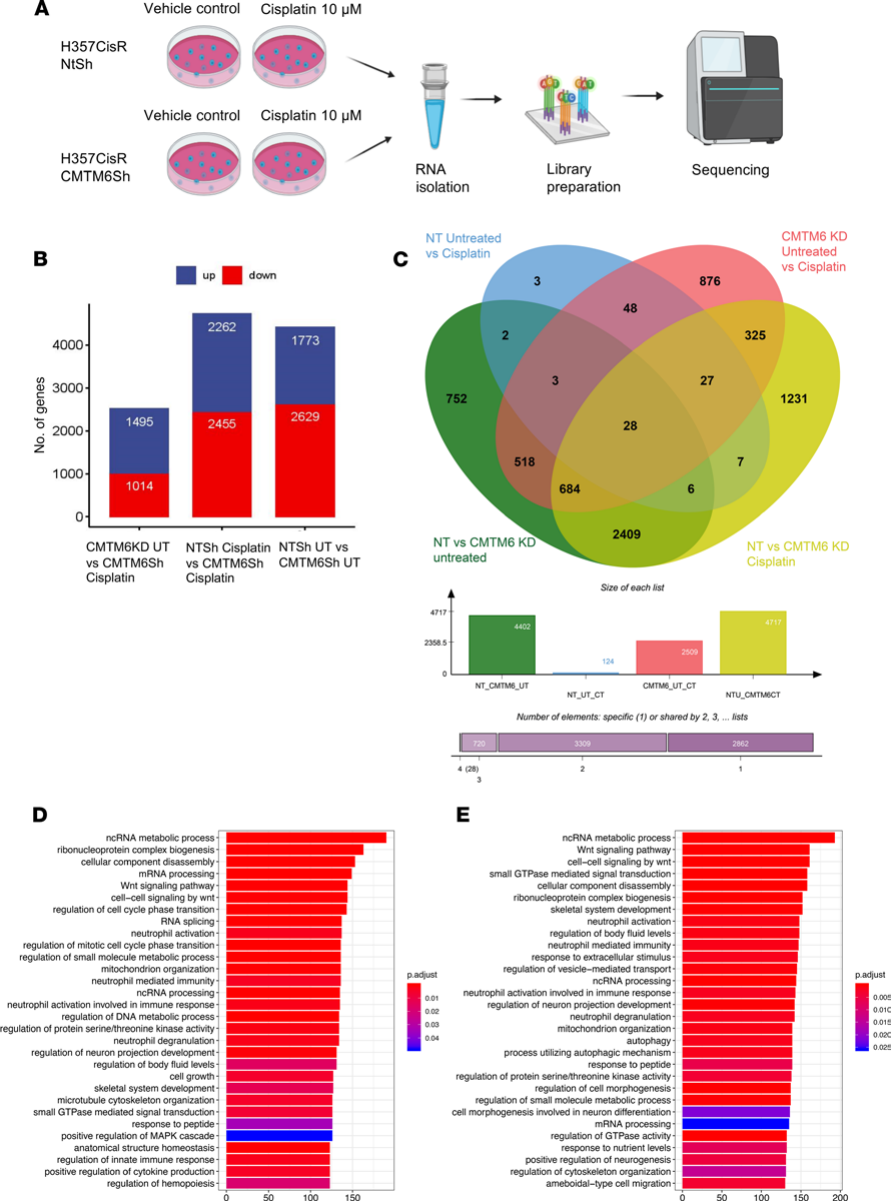
RNA-Seq revealed CMTM6 as a potential modulator of WNT signaling. (**A**) Schematic representation of workflow for RNA-Seq. (**B**) Histogram of dysregulated gene in CMTM6 Sh untreated (UT) versus CMTM6 Sh cisplatin treated, NT Sh UT versus CMTM6 Sh UT, NT Sh cisplatin treated versus CMTM6 Sh cisplatin treated. (**C**) Overlapping of differentially expressed genes from the previously mentioned comparisons. (**D** and **E**) Pathway enrichment analysis of NT Sh UT versus CMTM6 Sh UT, NT Sh cisplatin treated versus CMTM6 Sh cisplatin treated.

We compared the global gene expression profiles between CMTM6-KD and control vector–transduced (NTsh) chemoresistant cells and found 1773 genes significantly (>1.3-fold, FDR < 0.05) upregulated and 2629 genes downregulated (Figure 8, A and B). When we overlapped the differentially expressed genes between control versus CMTM6-KD cisplatin-untreated and -treated cells, more than 50% of the differentially regulated genes were similar in both lists (Figure 8C). Pathway enrichment analysis for the list of differentially regulated genes between NTsh untreated versus CMTM6-KD untreated cells (by the number of genes per Gene Ontology [GO] term) depicted significant enrichment of WNT signaling pathway (GO:0016055) with 144 genes from our data set (Figure 8D). In a similar manner, we compared gene expression profile between NTsh cisplatin-treated versus CMTM6-KD cisplatin-treated cells. The pathway enrichment analysis for this list of significantly and differentially regulated genes also depicted a large set of genes (*n* = 161), strongly supporting WNT signaling pathway (GO:0016055) (Figure 8E). We also compared the global gene expression differences between control untreated and control cells with cisplatin treatment. Here, we found that Wnt signaling was not significantly altered (Supplemental Figure 6). A heatmap of Wnt target genes from the transcriptome data set between NTSh and CMTM6Sh, with and without cisplatin treatment, depicted significant deregulation of Wnt target genes (Supplemental Figure 7, A and B). From the network analysis of WNT pathway genes of these comparisons, 3 major gene clusters were observed, i.e., (a) densely enriched with proteasome family members, (b) node enriched with Wnt family genes, and (c) transducing-like enhancer family genes (Supplemental Figure 7, C and D).

### CMTM6 modulates cisplatin resistance by regulating β-catenin expression.

**Figure 9:**
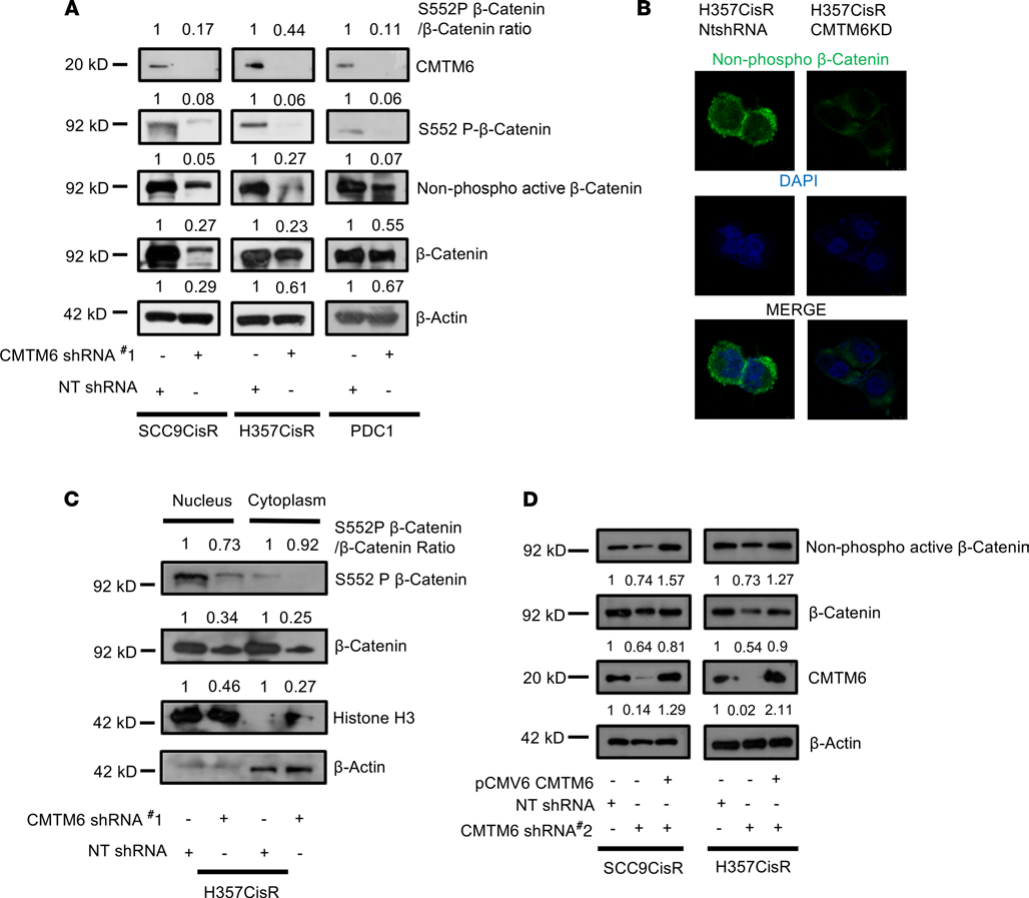
CMTM6 regulates β-catenin expression in chemoresistant OSCC. (**A**) The lysates were isolated and subjected to immunoblotting (*n* = 3) with indicated antibodies in chemoresistant cells stably expressing NTShRNA or CMTM6ShRNA#1. (**B**) Chemoresistant cells stably expressing NTShRNA or CMTM6ShRNA#1 were subjected to immunostaining and confocal microscopy with indicated antibodies. (**C**) Lysates from cytoplasm and nucleus of H357CisR cells stably expressing NTShRNA or CMTM6ShRNA#1 were separated, and immunoblot (*n* = 3) was performed against indicated antibodies. (**D**) CMTM6 was overexpressed in chemoresistant cells stably expressing NTShRNA or CMTM6ShRNA#2, and immunoblotting was performed with indicated antibodies.

**Figure 10:**
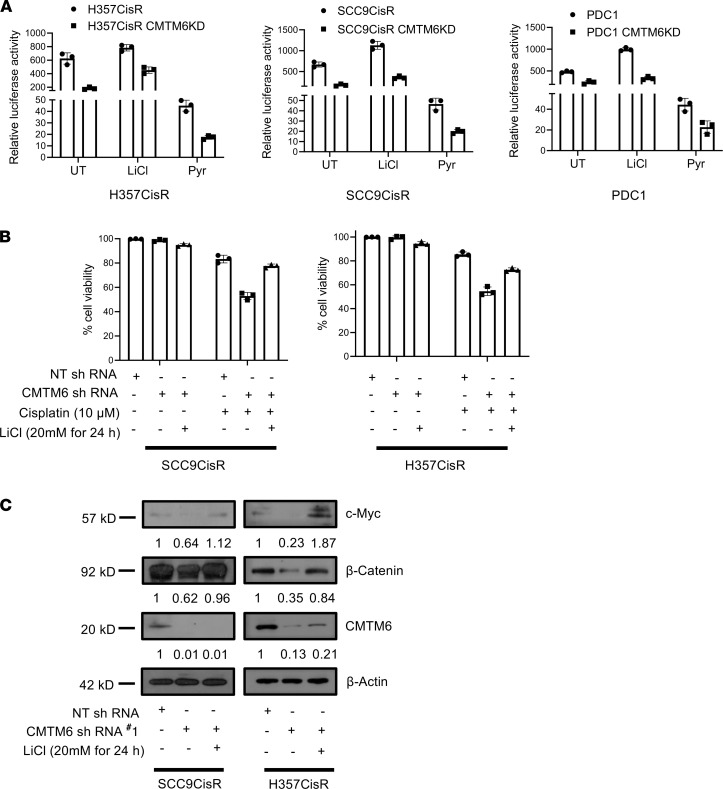
CMTM6 activates Wnt signaling in chemoresistant OSCC. (**A**) Chemoresistant cells stably expressing NTShRNA and CMTM6ShRNA#1 were cotransfected with either the TOPflash firefly vector and pRL Renilla control vectors following the treatment with LiCl (20 mM) or pyrvinium (50 μM) for 12 hours, and luciferase activity was measured as described in Methods. The bar diagram indicates the relative luciferase activity in each group (*n* = 3), 2-way ANOVA. (**B**) Cisplatin-resistant cells stably expressing NTShRNA and CMTM6ShRNA#1 were treated with cisplatin for 48 hours and LiCl for 24 hours, and cell viability was determined by MTT assay (*n* = 3), 2-way ANOVA. (**C**) Cisplatin-resistant cells stably expressing NTShRNA and CMTM6ShRNA#1 were treated with LiCl for 24 hours, and immunoblotting (*n* = 3) was performed with indicated antibodies.

**Figure 11:**
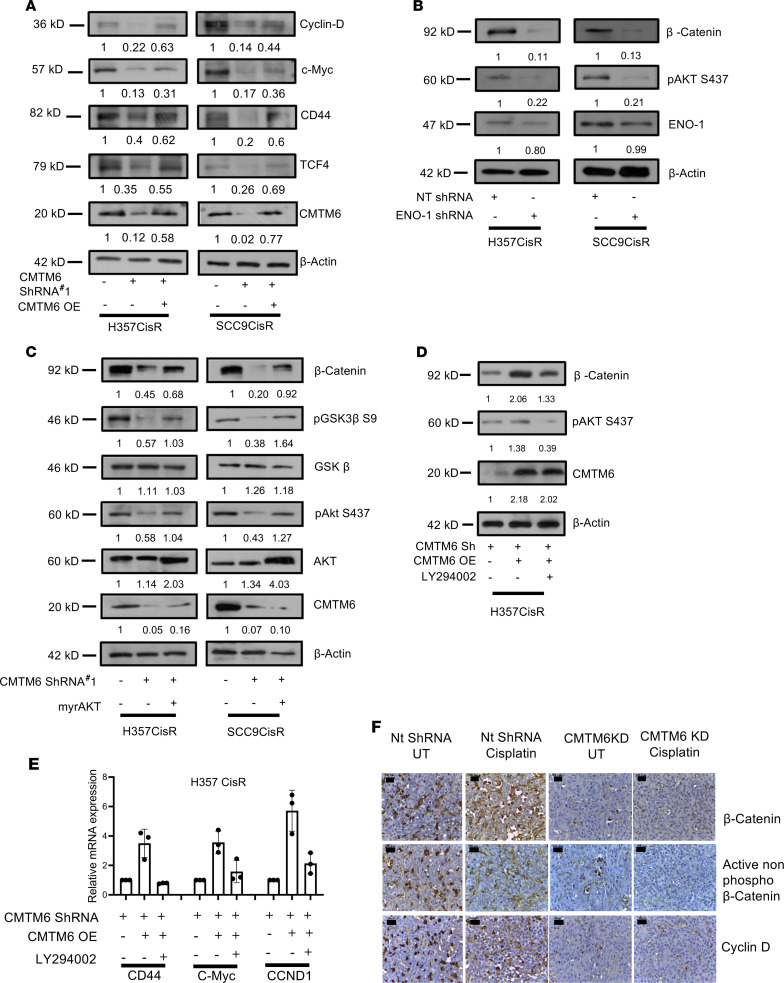
CMTM6 regulates Wnt signaling via the ENO-1/AKT/GSK3β/β-catenin axis. (**A**) CMTM6 was overexpressed in chemoresistant cells stably expressing NTShRNA or CMTM6ShRNA#2, and immunoblotting (*n* = 3) was performed with indicated antibodies. (**B**) Lysates were isolated from cisplatin-resistant lines stably transfected with NtSh and ENO1Sh and subjected to immunoblotting (*n* = 3) for indicated antibodies. (**C**) Myr AKT (constitutively active AKT) was overexpressed in chemoresistant cells stably expressing NTShRNA or CMTM6ShRNA#2, and immunoblotting (*n* = 3) was performed with indicated antibodies. (**D**) Lysates were isolated from LY294002-treated cells and vehicle control, and immunoblotting (*n* = 3) was performed using indicated antibodies. (**E**) mRNA was isolated from LY294002-treated cells and vehicle control, and relative mRNA (fold change) expression (*n* = 3) of indicated genes was analyzed by qRT-PCR in the indicated cell. Two-way ANOVA. (**F**) IHC of β-catenin and target genes in tumor of PDX model. Scale bar: 50 μm.

To investigate the mechanism of CMTM6 as a regulator of Wnt/β-catenin pathway in cisplatin resistance, we evaluated the expression of total β-catenin, p–β-catenin (S552 phosphorylated by AKT signaling) (19), as well as nonphosphorylated (S33/37/T41) active β-catenin. The immunoblotting and immunostaining data showed a substantial downregulation of β-catenin, p–β-catenin (S552), and nonphosphorylated active β-catenin in CMTM6-KD cells as compared with control cells (Figure 9, A and B, and Supplemental Figure 8A). Further, we evaluated β-catenin and p–β-catenin (S552) expression in nuclear and cytoplasmic fraction of cisplatin-resistant cells, which confirmed the reduced expression of both β-catenin and p–β-catenin (S552) in nuclear and cytoplasmic fraction of CMTM6-KD cells (Figure 9C). These data suggest that with the depletion of CMTM6, p-AKT S437 was reduced, in turn reducing p-GSK3β S9. Therefore, with the activation of GSK3β, total β-catenin was reduced, which also resulted in reduced expression of active β-catenin in CMTM6-depleted cisplatin-resistant lines. Again, reconstitution of CMTM6 in CMTM6-KD cells reversed active β-catenin expression, further complementing our observation (Figure 9D). In addition, stable CMTM6-KD chemoresistant cells depicted significantly reduced TOPflash luciferase activity, indicating diminished β-catenin/TCF-LEF–mediated transcriptional activity in both the presence and absence of Wnt-activator lithium chloride (LiCl) as well as Wnt inhibitor pyrvinium (Figure 10A). Similarly, MTT assay demonstrated the reversal of chemoresistant phenotype of CMTM6-KD cells by treating with Wnt activator LiCl (Figure 10B and Supplemental Figure 8B). In the presence of LiCl, we observed an upregulation of β-catenin and its target c-Myc in CMTM6-KD cells (Figure 10C). Moreover, we evaluated the effect of CMTM6 KD on downstream target genes of the Wnt/β-catenin signaling pathway. The immunoblotting and qRT-PCR data suggested that in CMTM6-KD chemoresistant cells, there was a significant downregulation of cyclin D, c-Myc, TCF4, and CD44, which were efficiently rescued upon ectopic overexpression of CMTM6 (Figure 11A and Supplemental Figure 8, C and D). Additionally, association analysis of CMTM6 mRNA levels with Wnt target genes (TCF4, LEF1, CD-44, MMP14, ENC1, ID2, PPARA, and JAG1) from the cancer genome atlas HNSCC cohort using GEPIA showed a positive correlation (*r* > 0.2) (Supplemental Figure 9). Further, upon Enolase-1 KD in chemoresistant cells, p-AKT S437 and β-catenin expression declined markedly (Figure 11B). Moreover, the myr-AKT overexpression (constitutively active AKT) in CMTM6-KD cells efficiently rescued p-GSK3β S9 and β-catenin expression (Figure 11C). Similarly, ectopic overexpression of CMTM6 in CMTM6-KD cells and treatment with AKT inhibitor LY294002 resulted in reduced expression of β-catenin and its target genes (Figure 11, D and E). In vivo data also suggested marked reduction of β-catenin and its target gene expression in CMTM6-depleted tumors (Figure 11F). Together, these observations indicate that CMTM6 regulation of cisplatin resistance works in ENO-1/AKT/GSK3β/β-catenin direction.

### Targeting CMTM6 suppresses CSC phenotypes.

**Figure 12:**
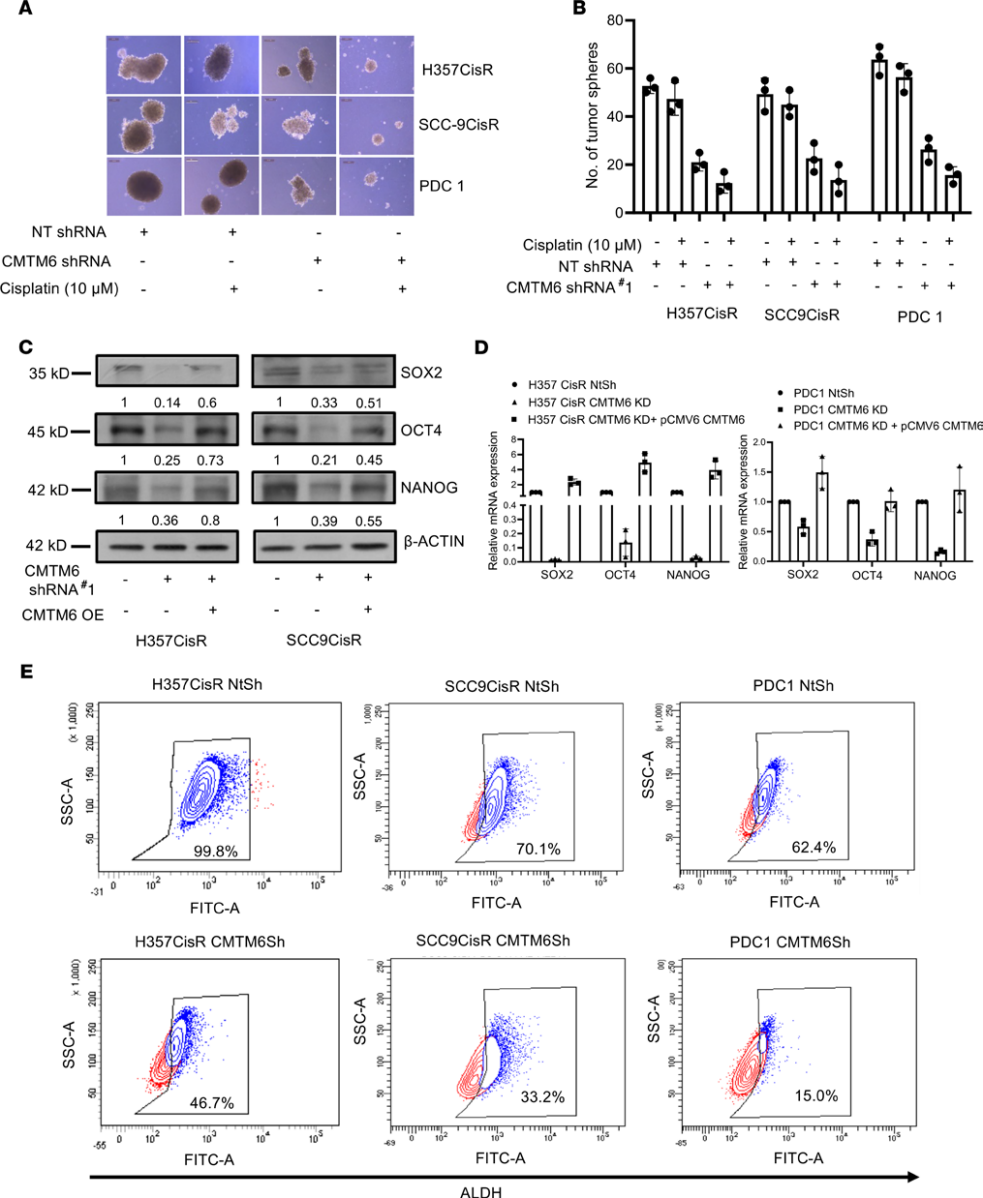
CMTM6 regulates stemness in chemoresistant cells. (**A**) Tumor spheroid assay was performed as described in Methods with chemoresistant cells stably expressing NTShRNA and CMTM6ShRNA#1 followed by treatment with indicated concentration of cisplatin for 5 days. At the end of the experiment, spheroid photographs were captured using Leica DMIL microscope. Scale bar, 200 μm. (**B**) Number of tumor spheres formed from the experiment in **A** was counted. (*n* = 3), 2-way ANOVA. (**C**) CMTM6 was overexpressed in chemoresistant cells stably expressing NTShRNA or CMTM6ShRNA#2, and immunoblotting (*n* = 3) was performed with indicated antibodies. (**D**) CMTM6 was transiently overexpressed in chemoresistant cells stably expressing CMTM6ShRNA#2, and relative mRNA (fold change) expression of indicated genes was analyzed by qRT-PCR in indicated cells (mean ± SEM, *n* = 3), 2-way ANOVA. (**E**) An ALDEFLUOR assay was conducted in chemoresistant cells stably expressing NTShRNA and CMTM6ShRNA#1, and the percentage of ALDH-high cells was quantified by flow cytometry (see Methods).

It is well established that Wnt signaling promotes stemness of cancer cells (20). Spheroid formation assay of cisplatin-treated CMTM6-KD chemoresistant cells showed reduced tumor spheroids (Figure 12, A and B). Hallmark stem cell marker expression was markedly reduced in CMTM6-KD chemoresistant cells, which in turn were rescued upon ectopic CMTM6 overexpression (Figure 12, C and D). Notably, aldehyde dehydrogenase (ALDH) activity assay clearly depicted that CMTM6 KD resulted in reduced ALDH activity in chemoresistant OSCC (Figure 12E). ABC transporters play an important role in acquired chemoresistance in cancer. Therefore, we measured ABC transporter expression in chemoresistant cells stably expressing NTSh or CMTM6ShRNA. The qRT-PCR data indicate reduced expression of major ABC transporters (ABCC1, ABCC2, ABCC3, ABCC4, ABCC5, and ABCG2) in CMTM6-depleted cells. Again, ABC transporter gene expression was elevated when we ectopically overexpressed CMTM6 in CMTM6-depleted cells (Supplemental Figure 10A). Association analysis of CMTM6 mRNA levels with ABC transporters from The Cancer Genome Atlas HNSCC cohort using GEPIA showed a positive correlation (*r* > 0.2) (Supplemental Figure 10B). Overall, CMTM6 regulates the Wnt/β-catenin signaling and cancer stemness augmenting chemoresistance in OSCC.

## Discussion

CT successfully eliminates the rapidly dividing cells in tumor mass but poorly targets the slowly dividing cells. Either these cells have inherent resistance properties, or they acquired chemoresistance during drug treatment. Owing to development of chemoresistance, patients continued to experience tumor growth with metastatic disease. The chemoresistance phenotypes can be attributed to reduced apoptosis, enhanced CSC population, altered metabolic activity, and decreased drug accumulation (6, 21, 22). All of these hallmarks are endpoint events, when the tumor cells have already acquired drug resistance. However, the precise causal process remains undetermined. Most studies to date have engaged the parental sensitive cells and late drug-resistant cells to understand the molecular mechanism for chemoresistance. On the contrary, in this study, we have performed global proteome profiling of parental sensitive, early, and late cisplatin-resistant cells. Per our hypothesis, addition of the early resistant group for proteome analysis may enable us to identify the key causative factors responsible for acquired chemoresistance.

Among the set of deregulated proteins in our proteome profiling, CMTM6 was the highest upregulated protein in early and late cisplatin-resistant cells as compared with the sensitive counterpart. Here, for the first time to our knowledge, we uncover another important biological function of CMTM6; i.e., it is a major driver of cisplatin resistance.

In this study, we performed pathway analysis of the set of proteins deregulated between sensitive, early, and late cisplatin-resistant cells. Our data suggest that CMTM6 regulates Wnt signaling through the AKT/GSK3β axis. In a previous study, Burr and colleagues (12) coimmunoprecipitated CMTM6 from digitonin lysate of pancreatic cancer cells and subjected it to mass spectrometry to explore the potential interacting partners of CMTM6. The data suggest that CMTM6 interacts with membrane Enolase-1, but the biological relevance of this interaction was not explored. In this study, the coimmunoprecipitation and confocal microscopy data suggest that CMTM6 interacted with membrane Enolase-1 and colocalized in plasma membrane. We also demonstrate here that knocking down CMTM6 reduced membrane Enolase-1 expression. It is well documented by other groups that Enolase-1 enhances the phosphorylation of AKT and GSK3β (18, 23). Therefore, we predict that CMTM6 stabilizes membrane Enolase-1 expression and activates the AKT/GSK3β-mediated Wnt signaling. It is evident from previous reports that Wnt/β-catenin signaling plays an important role in acquiring chemoresistance as it regulates the cancer stemness (24–26). Here, we have also demonstrated that knocking down CMTM6 significantly reduced the stemness properties of chemoresistant cells. Overall, we uncover the potentially novel mechanism by which CMTM6 regulates Wnt signaling and mediates cisplatin resistance in OSCC.

In conclusion, it was earlier established that CMTM6 is a novel protein, which stabilizes PD-L1 and potentiates the immune evasion by tumor cells. For the first time to our knowledge, we demonstrate CMTM6 is a major driver of cisplatin resistance. Therefore, targeting CMTM6 can be a useful strategy to overcome therapy resistance in advanced squamous cell carcinomas.

## Methods

### Cell culture.

H357, SCC9, and SCC4 (human tongue OSCC) cell lines were obtained from MilliporeSigma, sourced from a European collection of authenticated cell culture. All OSCC cell lines were cultured and maintained as described earlier. A549 (lung carcinoma) and A375 (melanoma) cell lines were obtained from National Centre for Cell Science, Pune, and were maintained in DMEM supplemented with 10% FBS (Thermo Fisher Scientific) penicillin–streptomycin (Pan Biotech).

### Generation of early and late cisplatin-resistant cell lines.

For establishment of the cisplatin-resistant cell line, H357, SCC9, and SCC4 (OSCC), A549 (lung carcinoma), and A375 (melanoma) cell lines were initially treated with cisplatin at 1 μM (lower dose) for 1 week, and then the cisplatin concentration was increased gradually up to IC_50_ value, i.e., 15 μM for H357, SCC9, SCC4, and A549, and 10 μM for A375 in a span of 3 months. Parental cells were grouped as sensitive (CisS), and after a period of 4 and 8 months of treatment, were termed as early (CisR 4M) and late resistant (CisR 8M) cells, respectively.

### iTRAQ-based proteomics analysis.

Harvested cells (5 × 10^6^) from 3 time points (0 month, 4 months, and 8 months) were treated with RIPA buffer (Thermo Fisher Scientific, catalog: 88665) supplemented with protease and phosphatase inhibitor (MilliporeSigma, catalog: P0044). Extracted cellular proteins from all 3 time points with appropriate technical and biological replicates were used in an isobaric tag for the relative and absolute quantification (iTRAQ) experiment (Figure 1B). Equal amounts of proteins (100 μg) from all samples were taken for tryptic protein preparation following the manufacturer’s instructions (AB Sciex). Study samples with the tag details used for labeling in the iTRAQ experiment are presented in Figure 1B. Trypsin treatment was performed using trypsin supplied by the manufacturer and incubated at 37°C for 16–20 hours. Tryptic peptides were dried at 40°C using SpeedVac (LabConco). Dried tryptic peptides were dissolved using dissolution buffer, and isobaric tags reconstituted with isopropanol were added for incubation at room temperature for 2 hours. After completion of the reaction, tagged tryptic peptides from all samples were pooled and dried. Tagged tryptic peptides (approximately 250 μg) were subjected to strong cation exchange fractionation using a handheld ICAT Cartridge-cation-exchange system (Applied Biosystems). Peptides were eluted using a gradient of 30, 50, 80, 120, 250, 300, 400, and 500 mM ammonium formate solutions with a flow rate of 10 drops per minute. Each SCX fraction was dried at 40°C using a Speed Vac (CentriVap, Labconco) and cleaned using a Pierce C18 spin column (Thermo Fisher Scientific).

Each SCX fraction was resuspended in 20 μL buffer (water with 0.1% formic acid) and introduced to the easy-nano-LC 1000 HPLC system (Thermo Fisher Scientific) connected to hybrid Orbitrap Velos Pro Mass Spectrometer (Thermo Fisher Scientific). The nano-LC system contains the Acclaim PepMap100 C18 column (75 μm × 2 cm) packed with 3 μm C18 resin connected to the Acclaim PepMap100 C18 column (50 μm × 15 cm) packed with 2 μm C18 beads. A 120-minute gradient of 5%–90% buffer B (0.1% formic acid in 95% acetonitrile) and buffer A (0.1% formic acid in 5% acetonitrile) was applied for separation of the peptide with a flow rate of 300 nL/min. The eluted peptides were electrosprayed with a spray voltage of 1.5 kV in positive ion mode. Mass spectrometry data acquisition was carried out using a data-dependent mode to switch between MS1 and MS2.

### Protein identification and iTRAQ quantitation.

Protein identification and quantification were carried out using SEQUEST search algorithm of Proteome Discoverer Software 1.4 (Thermo Fisher Scientific). Each MS/MS spectrum was searched against a human proteome database (UniProt, 89,796 total proteins, downloaded in April 2017). Precursor ion mass tolerance (20 ppm), fragmented ion mass tolerance (0.1 Da), missed cleavages (<2) for trypsin specificity, carbamidomethyl (C), deamidation (N and Q), oxidation (M), and 8-plex iTRAQ label (N terminus and K) were set as variable modifications. The FDR at both protein and peptide level was calculated at 5%. The identified protein list with fold change values were exported to Microsoft Excel for further statistical analysis. Identified proteins from study samples and relative fold change values were selected for PCA, and a partial least-squares discriminant analysis model was built using MetaboAnalyst 3.0. Proteins with at least 2.0-fold change (log_2_ resistance/sensitive > ±1.0, *P* < 0.05, variable important projection value > 1.0) were selected as deregulated proteins. All mass spectrometry data files (.raw and.mgf) with result files were deposited in the ProteomeXchange Consortium (PXD016977). The deregulated proteins, identified from global proteomics analysis, were converted to a gene list, and a functional analysis was generated using IPA.

### Lentivirus production and generation of stable CMTM6-KD and ENO-1–KD cell lines.

ShRNAs targeting CMTM6 were cloned into pLKO.1 vector as per the Addgene protocol. Cloning of shRNAs was followed by confirmation using sequencing method. Lentivirus was produced by transfection of pLKO.1-shRNA plasmid along with packaging plasmid psPAX2 and envelope plasmid pMD2G into HEK293T cells (American Type Culture Collection). Lentivirus particles were generated using the protocol as described in Shriwas et al. (27). Lentivirus-infected cells were incubated with puromycin up to 5 μg/mL for 2 weeks, and stable clones were picked and confirmed by immunoblotting. All shRNA sequences used in this study are mentioned (Supplemental Table 2).

### Transient transfection and overexpression of CMTM6 as well as myr-AKT in CMTM6-KD cell lines.

CMTM6-KD cells, stably expressing shRNA#2 targeting 3′UTR of CMTM6 mRNA, were transiently transfected with pCMV6 CMTM6 (Myc-DDK-tagged) (Origene catalog: RC201061) using the ViaFect transfection reagent (Promega catalog: E4982). The transfection efficiency was confirmed by immunoblotting against anti-CMTM6 and anti-DDK. 901 pLNCX myr HA Akt1 (Addgene plasmid 9005) was used for transient overexpression of active AKT. The myr HA Akt1 vector was deposited to Addgene by Sellers WR laboratory.

### OSCC patient sample.

All samples collected were locoregionally advanced OSCC. Neoadjuvant CT had been prescribed before surgery and/or radiotherapy. The 3-drug combination like TPF was having highest response (TAX 324). However, some cases had poor Eastern Cooperative Oncology Group performance status; hence 2 drugs (TP) were prescribed instead of TPF. After CT the response was evaluated as per Response Evaluation Criteria In Solid Tumors criteria by clinical and radiological evaluation. If there was no evidence of malignancy, then it was diagnosed as complete response (CR). If the target lesions had decreased more than equal to 30% of the sum of the longest diameter, then it was diagnosed as partial response (PR). If there was no sign of either CR or PR, then it was called stable disease, and if the target lesions had increased more than or equal to 20% of the sum of the longest diameter, then it was called PD (progressive disease). As the patients showing CR and PR responded to the CT, they were categorized as responders, and the patients with stable disease or PD with almost no response to CT were categorized as nonresponders. The Human Ethics Committee (HEC) of the Institution of Life Sciences approved all patient-related studies, and informed consent was obtained from all patients. Study subject details with treatment modalities are presented in Supplemental Table 1, A and B.

### Immunoblotting.

Cell lysates were used for immunoblotting experiments as described earlier (28). For this study, we used antibody against β-actin (MilliporeSigma, catalog: A2066), PARP (Cell Signaling Technology [CST], catalog: 9542L), p–S139-H2AX (CST, catalog: 9718S), CMTM6 (MilliporeSigma, catalog: HPA026980), DDK (CST: catalog: 14793), AKT (CST, catalog: 9272S), p-AKT (S473) (CST, catalog: 4058S), GSK3β (CST, catalog: 9315s), p-GSK3β (S9) (CST, catalog: 9323S), β-catenin (CST, catalog: 9562), p–β-catenin (S552) (CST, catalog: 9566), nonphosphorylated (active) β-catenin (S33/37/T41) (CST, catalog: 8814), CD44 (Novus, catalog: NBP1-31488), TCF4/TCF7L2 (C48H11) (CST, catalog: 2569S), c-Myc (CST, catalog: 9402), cyclin D1 (CST, catalog: 2922S), LRP1 (Cloud clone, catalog: PAB010Hu01), PSMD2 (Cloud clone, catalog: PAG279Hu01), Enolase (L-27) (Santa Cruz Biotechnology, catalog: sc-100812), Sox2 (CST, catalog: 2748), Nanog (CST, catalog: 4893S), and Oct-4 (CST, catalog: 2750S). Membrane and cytoplasmic fractions were separated using the Mem-PER Plus Membrane Protein Extraction Kit (Thermo Fisher Scientific, catalog: 78833), according to the manufacturer’s instructions. The band intensity in all immunoblots (*n* = 3) was quantified using ImageJ software (NIH). The mean value of band intensity is indicated under the immunoblots. The intensity for each blot was calculated by normalizing with the intensity of β-actin blot.

### Coimmunoprecipitation.

For coimmunoprecipitation experiments, cells were lysed in 1% digitonin for 30 minutes on ice. The lysates were incubated with primary antibody for 1–4 hours, followed by addition of Protein A/G PLUS-Agarose beads (Santa Cruz Biotechnology, catalog: sc-2003) overnight at 4°C. After 4 washes in 0.2% digitonin, samples were eluted in SDS sample buffer with 50 mM DTT for 10 minutes at 70°C, separated by SDS-PAGE, and immunoblotted. VeriBlot for IP Detection Reagent (HRP) (Abcam, catalog: ab131366) was used for immunoblotting.

### Patient-derived xenograft.

BALB/C-nude mice (6–8 weeks, male, NCr-Foxn1nu athymic) were purchased from Vivo Bio Tech Ltd. For the xenograft model, early passage PDC1 cells established from the CT nonresponder patient (treated with TP, 50 mg carboplatin and 20 mg paclitaxel for 3 cycles without having any response) were used. Two million cells were suspended in PBS-Matrigel (1:1, 100 μL) and transplanted into upper flank of mice. The PDC1 cells stably expressing NtShRNA were injected in right upper flank and PDC1 cells CMTM6ShRNA#1 (PDC1 CMTM6KD) were injected in the left upper flank of same mice. These mice were randomly divided into 2 groups (*n* = 6) once the tumor reached volume of 50 mm^3^ and injected with vehicle or cisplatin (3 mg/kg) intraperitoneally twice a week. Tumor size was measured using digital Vernier caliper twice a week until the completion of the experiment. Tumor volume was determined using the following formula: tumor volume (mm^3^) = (minimum diameter)^2^ × (maximum diameter)/2.

### RT-PCR and qRT-PCR.

RNA mini kit (Himedia, catalog: MB602) was used to isolate total RNA according to the manufacturer’s instructions and quantified by NanoDrop (Thermo Fisher Scientific). cDNA was synthesized by RT-PCR using Verso cDNA synthesis kit (Thermo Fisher Scientific, catalog: AB1453A) from 300 ng RNA. qRT-PCR was carried out using SYBR Green master mix (Thermo Fisher Scientific, catalog: 4367659). GAPDH was used as a loading control. The primer (oligonucleotides) details used for qRT-PCR in this article are listed (Supplemental Table 2).

### IHC.

IHC of formalin-fixed, paraffin-embedded samples (OSCC patients’ tumor and xenograft tumors from mice) was performed as previously described (29). Antibodies against CMTM6 (MilliporeSigma, catalog: HPA026980), PD-L1 (Invitrogen, catalog PA5-20343), β-catenin (CST, catalog: 9562), nonphosphorylated (active) β-catenin (S33/37/T41) (CST, catalog: 8814), cyclin D1 (CST, catalog: 2922S), and Ki67 (Vector, catalog: VPRM04) were used for IHC. Images were obtained using Leica DM500 microscope. Q score was calculated by multiplying percentage of positive cells with staining (P) and intensity of staining (I). P was determined by the percentage of positively stained cells in the section. I was determined by the intensity of the staining in the section; i.e., strong (value = 3), intermediate (value = 2), weak (value = 1), and negative (value = 0).

### Annexin V PE/7AAD assay.

Apoptosis and cell death assay was performed using Annexin V Apoptosis Detection Kit PE (eBioscience, catalog: 88-8102-74) as previously described (27), and cell death was monitored using a flow cytometer (BD FACS Fortessa).

### Assessment of cell viability.

Cell viability was measured by MTT assay (MilliporeSigma).

### Immunofluorescence.

Cells were seeded on the lysine-coated coverslip and cultured overnight. Cells were fixed with 4% formaldehyde for 15 minutes, permeabilized with 1× permeabilization buffer (eBioscience 00-8333-56), and blocked with 3% BSA for 1 hour at room temperature. Then, cells were incubated with primary antibody overnight at 4°C, washed 3 times with PBS-Tween (PBST), followed by 1 hour of incubation with goat anti–rabbit IgG (H+L) secondary antibody, Alexa Fluor 488 conjugate (Invitrogen, catalog: A -11008), and rabbit anti–mouse IgG (H+L) secondary antibody, Alexa Fluor 647 conjugate (Invitrogen, catalog: A – 21239). After final wash 3 times with PBST, coverslips were mounted with DAPI (Slow Fade GOLD Antifade, Thermo Fisher Scientific, catalog: S36938). Images were captured using a confocal microscopy (LEICA TCS-SP8). Anti-CMTM6 (MilliporeSigma, catalog: HPA026980), Anti–α-Enolase (L-27) (Santa Cruz Biotechnology, catalog: sc-100812), and anti–nonphosphorylated (active) β-Catenin (S33/37/T41) (CST, catalog: 8814) were used in this study.

### Colony formation assay.

Colony formation assay was performed as previously described in Shriwas et al. (27).

### Library preparation and RNA-Seq.

RNA-Seq library preparation was performed for H357 CisR Nt Sh and H357 CisR CMTM6 Sh treated with vehicle control and cisplatin (10 μM) for 24 hours. We have included 2 independent biological replicates to identify the CMTM6 downregulation-mediated global transcriptome changes. For RNA-Seq library preparation, 2 μg total RNA was used to isolate mRNA through magnetic beads using mRNA isolation kit (PolyA mRNA Isolation Module, NEB) followed by RNA-Seq library preparation using mRNA library preparation kit (NEB) strictly following the vendor’s recommended protocol. After library preparation, library concentration was estimated using qubit 2.0 (Invitrogen), and the recommended fragmentation sizes were confirmed by Bio-analyzer (Agilent).

### Dual luciferase reporter assay.

For this assay cells were cotransfected with M50 Super 8x TOPFlash (which was a gift from Randall Moon, University of Washington Institute for Stem Cell and Regenerative Medicine, Seattle, Washington, USA; Addgene catalog: 12456) (30) and pRL-TK (Promega) in a ratio of 100:1 using ViaFect transfection reagent (Promega catalog: E4982). Seven TCF/LEF-binding sites are present upstream of a firefly luciferase gene in the TOPflash vector, whereas pRL-TK provides constitutive expression of Renilla luciferase and was used as an internal control for the experiment. Cells were treated with vehicle control, LiCl (MilliporeSigma, catalog: 62476-100G-F), and Pyrvinium (MilliporeSigma, catalog: P0027-10MG) 48 hours after transfection, and luciferase reading was taken using the Dual-Glo luciferase assay kit (Promega, catalog: E1910) according to the manufacturer’s instructions.

### Tumor sphere formation assay.

A total of 1000 cells were seeded on 6-well ultralow attachment plates (Corning-Costar, Inc.) and were grown with 1× B27 (Invitrogen 17502048), 1× N2 supplement (Invitrogen 17502048), 20 ng/mL human recombinant epidermal growth factor (Invitrogen PHG0313), and 10 ng/mL basic fibroblast growth factor (Invitrogen PHG0263) in serum-free DMEM-F12 medium (Pan biotech P04-41500). After spheroid formation, treatment was done with DMSO and cisplatin. Images were captured using a microscope (LEICA DMIL).

### ALDH activity assays.

ALDH activity was detected using an ALDEFLUOR assay kit (StemCell Technologies, catalog: 1700) according to the manufacturer’s protocol. Cells were incubated with ALDH protein substrate (BAAA) in ALDEFLUOR assay buffer for 30 minutes at 37°C. A specific inhibitor of ALDH (DEAB) was used as a negative control. Fluorescence was measured by BD LSR Fortessa.

### Correlation analysis of β-catenin target genes and CMTM6.

The correlation analysis was performed between β-catenin target genes and CMTM6 in HNSCC patient tumors using GEPIA (http://gepia.cancer-pku.cn/detail.php?gene=CMTM6) online analysis software based on the TCGA database and Genotype, using |log_2_FC|≥1 and a *P* value of less than or equal to 0.05 as the cutoff criteria.

### Relapse-free survival analysis.

The relapse-free survival value of various cancer types was plotted for CMTM6 and PD-L1 using KM Plotter (http://kmplot.com/analysis/index.php?p=service).

### Data availability.

Proteome files were deposited in the ProteomeXchange Consortium with accession number PXD016977, and raw files for RNA-Seq data were deposited in European Bioinformatics Institute’s ArrayExpress platform with E-MTAB-9424 as the accession number.

### Statistics.

All data are shown as the mean ± SD, and GraphPad Prism 5.0 was used for calculation. The statistical significance was calculated by 1- and 2-way ANOVA, and a *P* value of less than or equal to 0.05 was considered significant.

### Study approval.

This study was approved by the IRB and HEC of Institute of Life Sciences, Bhubaneswar (84/HEC/18), and All India Institute of Medical Sciences, Bhubaneswar (T/EMF/Surg.Onco/19/03). The animal-related experiments were performed in accordance to the protocol approved by Institutional Animal Ethics Committee of Institute of Life Sciences, Bhubaneswar (ILS/IAEC-190-AH/DEC-19). Approved procedures were followed for patient recruitment, and after receiving written informed consent from each patient, tissues samples were collected.

## Author contributions

PM, OS, SM, RA, and SRK, under direction of RD and RKN, designed and performed the experiments, analyzed the data, and wrote the first version of the manuscript. AG, SS, SKR, RR, DKM, and SKM performed part of the experiments. PM, OS, and RD designed the experiments and supervised the study. RD and RKN wrote the manuscript. All authors approved the final version.

## Supplementary Material

Supplemental data

## Version 1 01/12/2021

In-Press Preview

## Version 2 02/22/2021

Electronic publication
