# Supplementary material for: CMTM6 drives cisplatin resistance by regulating Wnt signaling through the ENO-1/AKT/GSK3β axis
Source: JCI Insight. 2021 Feb 22;6(4):e143643. doi: 10.1172/jci.insight.143643 (PMC7934946; doi:10.1172/jci.insight.143643)
Supplement: Supplemental data [file jciinsight-6-143643-s204.pdf]

Supplementary Figure 1

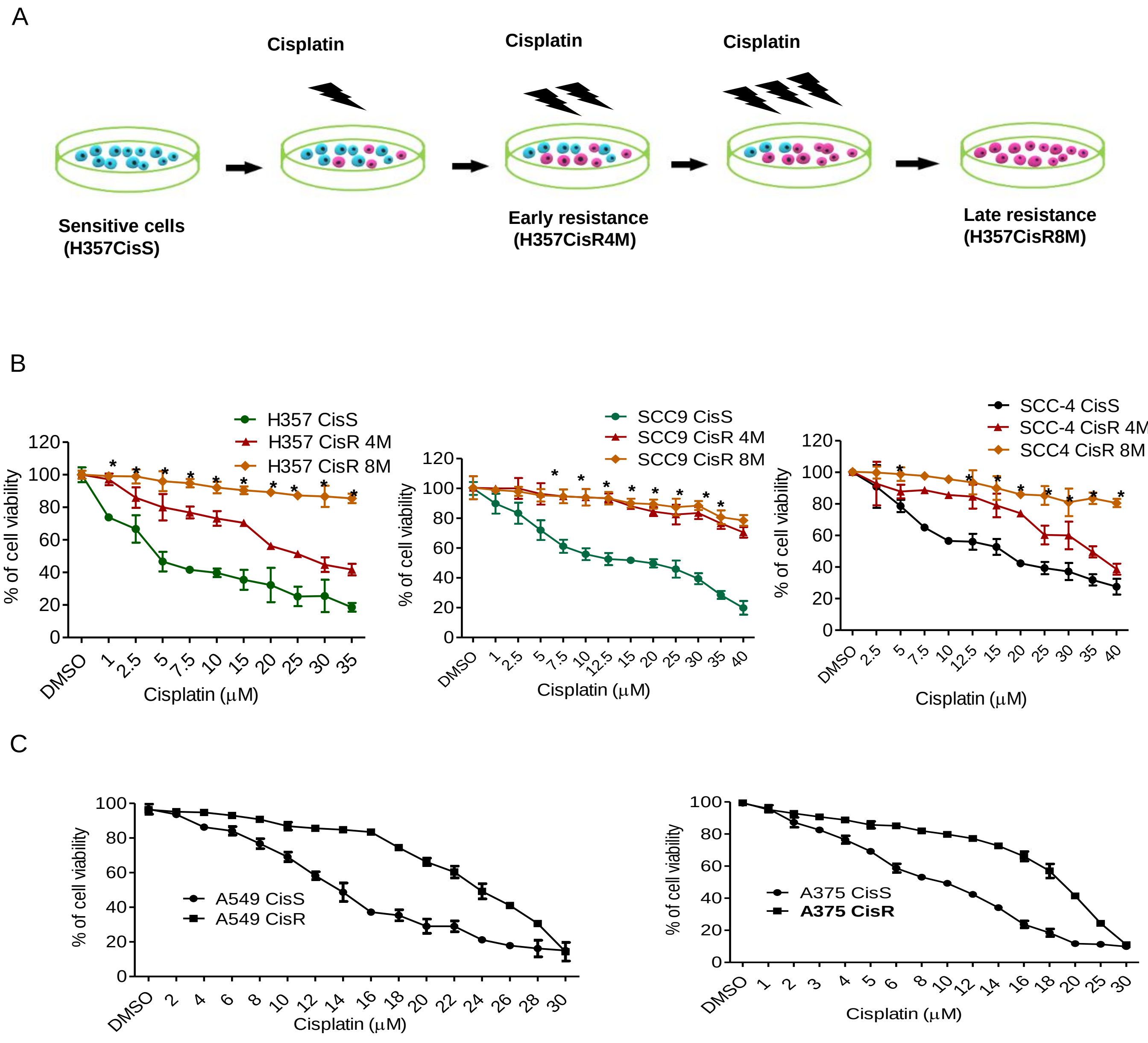

Supplementary figure 1: Characterization of sensitive, early and late cisplatin resistant lines: A) Schematic presentation of establishing sensitive, early and late cisplatin resistant cancer lines **B)** Sensitive, early and late cisplatin resistant pattern (CisS, CisR4M and CisR8M) of H357, SCC9 and SCC4 cells were treated with indicated concentrations of cisplatin for 48h and cell viability was determined by MTT assay (n=3, *: $P < 0.05$). **C)** Sensitive and late cisplatin resistant lung cancer (A549) and melanoma (A375) lines were established as described in method section. Sensitive and resistant cells were treated with cisplatin with indicated concentrations for 48h and cell viability was determined by MTT assay (n=3 *: $P < 0.05$).

Supplementary Figure 2

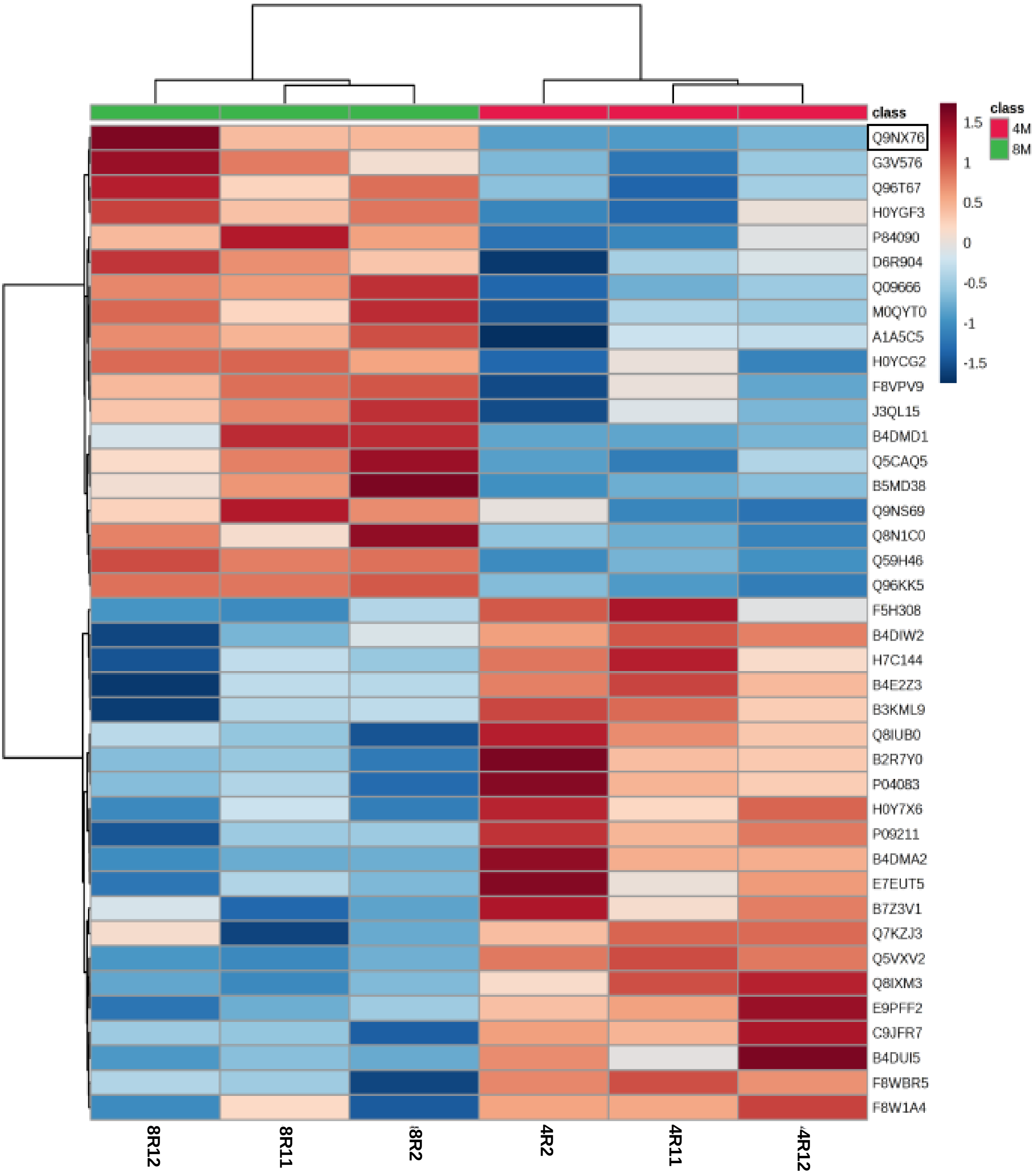

Supplementary figure 2: Global proteomic profiling of sensitive, early and late chemoresistant cells. The lysates were isolated from parental sensitive (H357CisS), early (H357CisR4M) and late (H357CisR8M) cisplatin resistant cells and subjected to global proteomic profiling. The dendrogram represents the dysregulated genes from proteomic analysis between early (H357CisR4M) and late (H357CisR8M) cisplatin resistant cells after normalizing with parental sensitive (H357CisS) cells.

Supplementary Figure 3

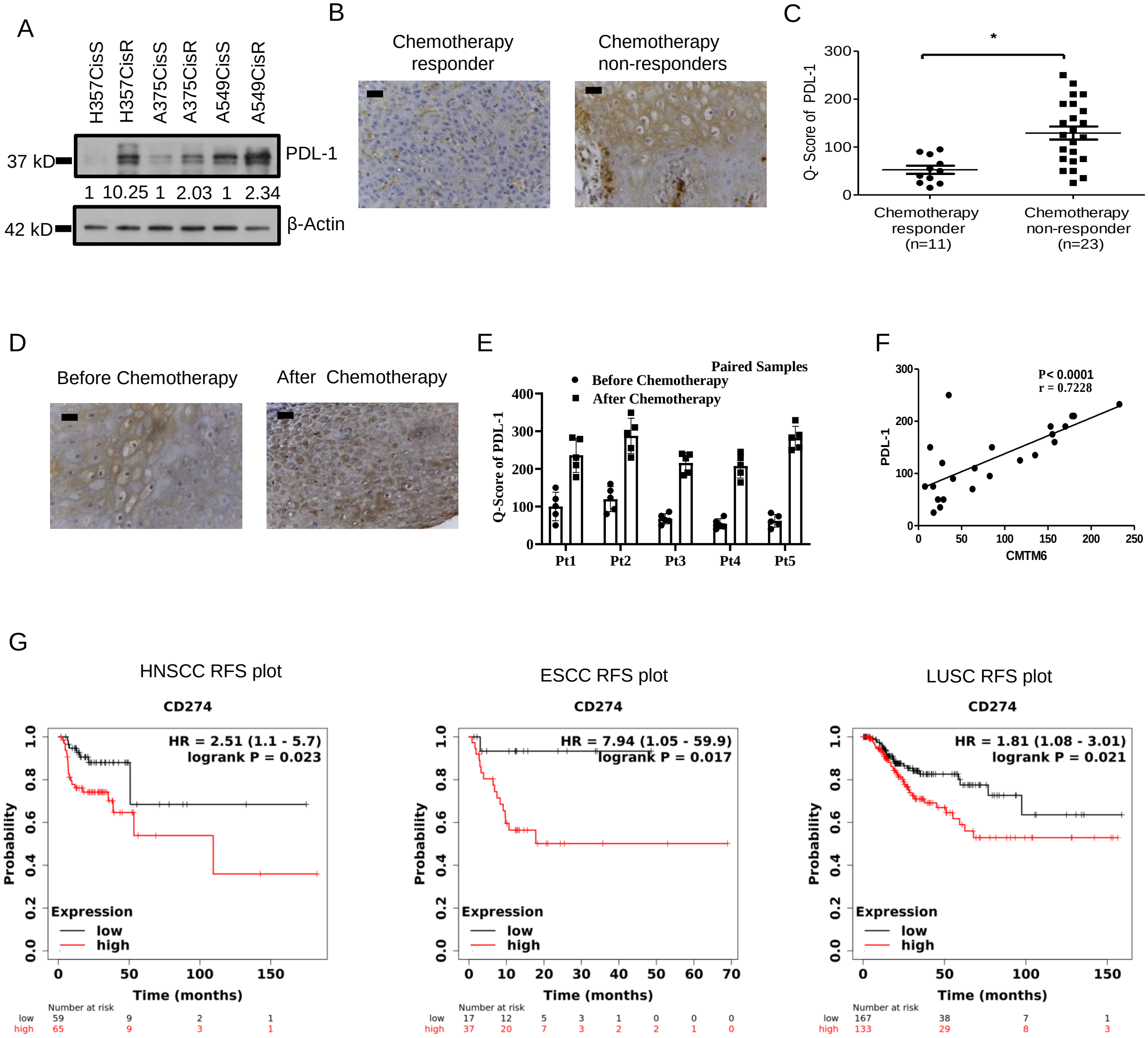

Supplementary figure 3: PDL1 expression is elevated in cisplatin resistant carcinomas:
A) Cell lysates from indicated cisplatin resistant and sensitive cancer lines were isolated and subjected to immunoblotting (n=3) against PDL1 and β-actin antibodies. B) Protein expression of PDL1 was analyzed by IHC in chemotherapy-responder and chemotherapy-non-responder OSCC tumors C) IHC scoring of PDL1 (Median, n=11 for chemotherapy-responder and n=23 for chemotherapy-non-responder) *: $P < 0.05$. D) Protein expression of PDL1 was analyzed by immunohistochemistry (IHC) in pre- and post-TPF treated paired tumor samples for chemotherapy-non-responder patients E) IHC scoring of CMTM6 (n=6) F) Co-relation analysis of CMTM6 and PDL1 in chemotherapy-non-responder patients (n=23) G) Relapse Free Survival (RFS) plot for PDL1 using KM-Plotter. *For IHC scoring, Q Score =Staining Intensity × % of IHC Staining.

Supplementary Figure 4

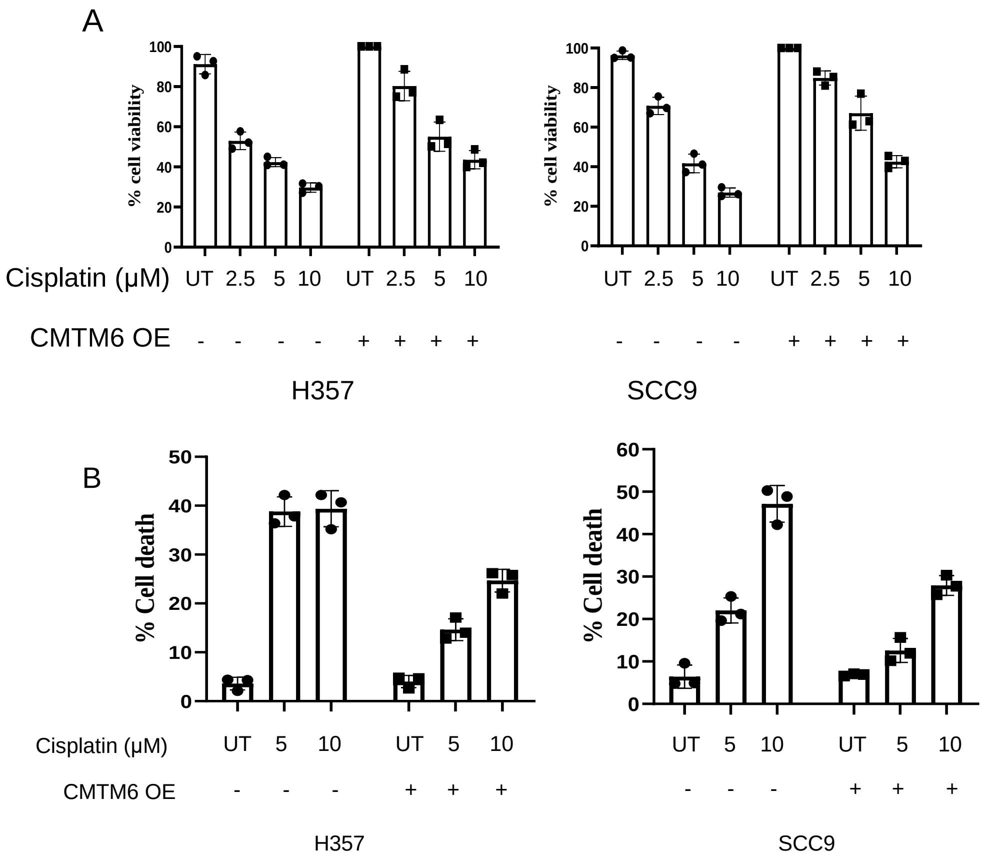

Supplementary figure 4: Ectopic overexpression of CMTM6 induced drug resistance phenotype in Cisplatin sensitive cells: A) CMTM6 was overexpressed in cisplatin sensitive H357 and SCC9 cell lines and treated with cisplatin with indicated concentration for 48h. Cell viability was determined by MTT (n=3), *: $P < 0.05$ by **B)** Cells were treated as described in panel E and **c**ell death was determined by annexin V/7AAD assay using flow cytometer (n=3).

Supplementary Figure 5

A

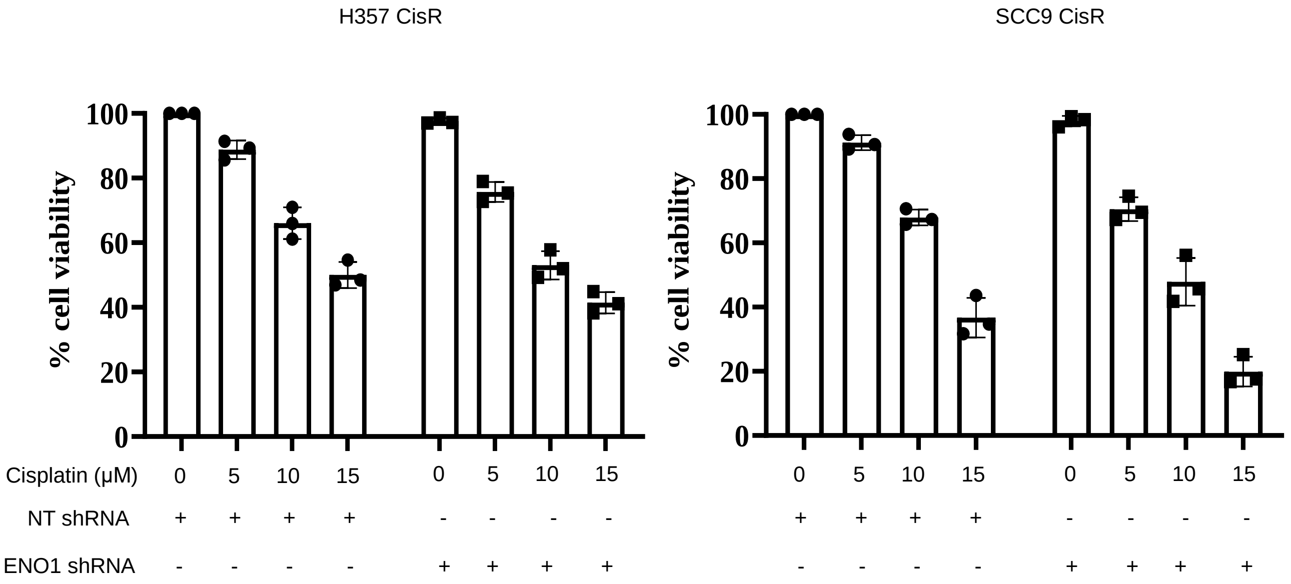

B

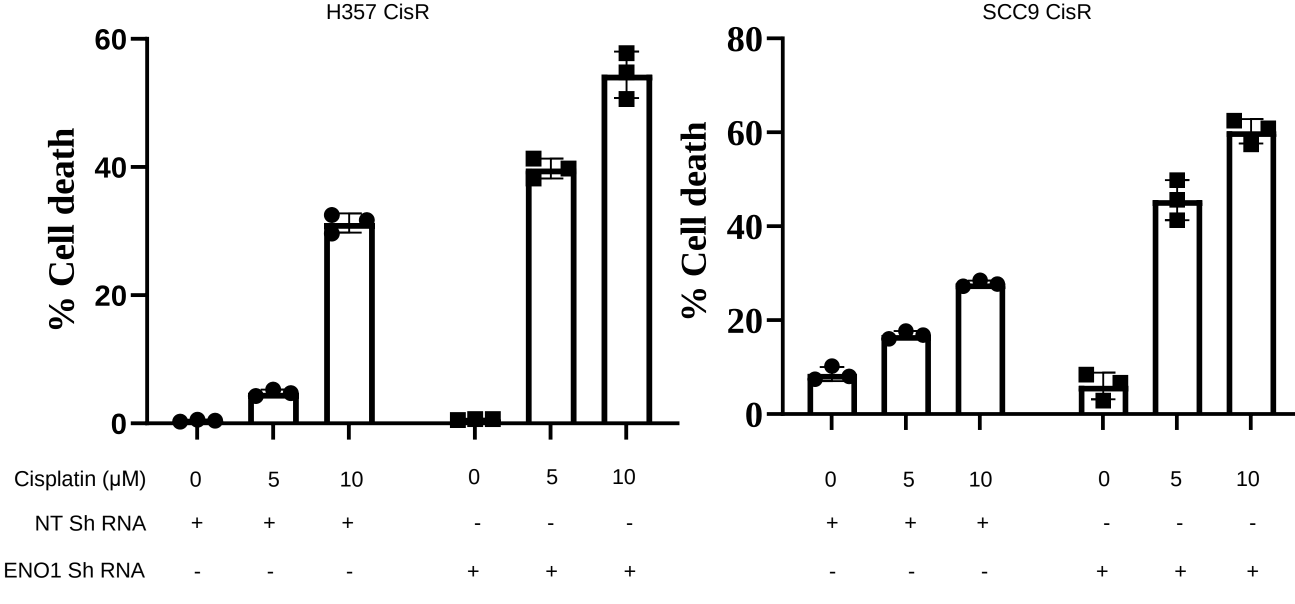

Supplementary figure 5: **ENO1 knockdown re-sensitises chemoresistant cells to cisplatin** : A) Cisplatin resistant H357 and SCC9 cells stably expressing NTShRNA and ENO1ShRNA were treated with cisplatin for 48h and cell viability was determined by MTT assay B) Cisplatin resistant cells stably expressing NTShRNA and ENO1ShRNA were treated with cisplatin for 48h and after which cell death was determined by annexin V/7AAD assay using flow cytometer.

Supplementary Figure 6

Nt Sh UT Vs Nt Sh Cisplatin

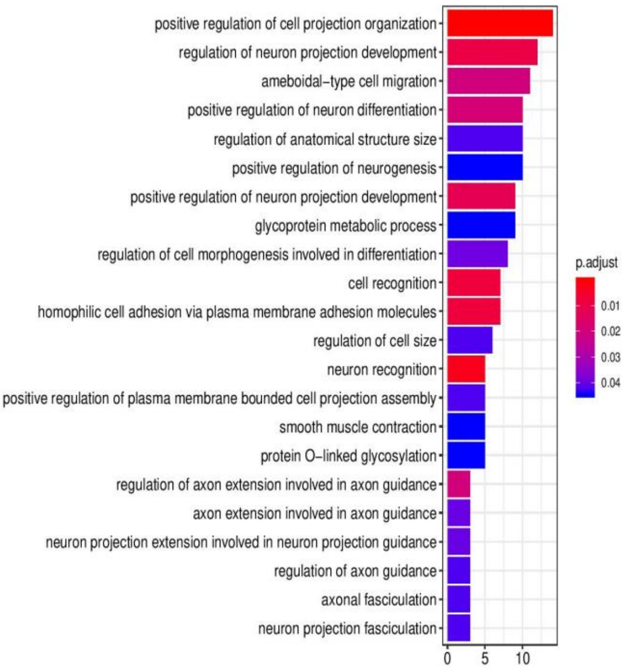

CMTM6 Sh UT Vs CMTM6 Sh Cisplatin

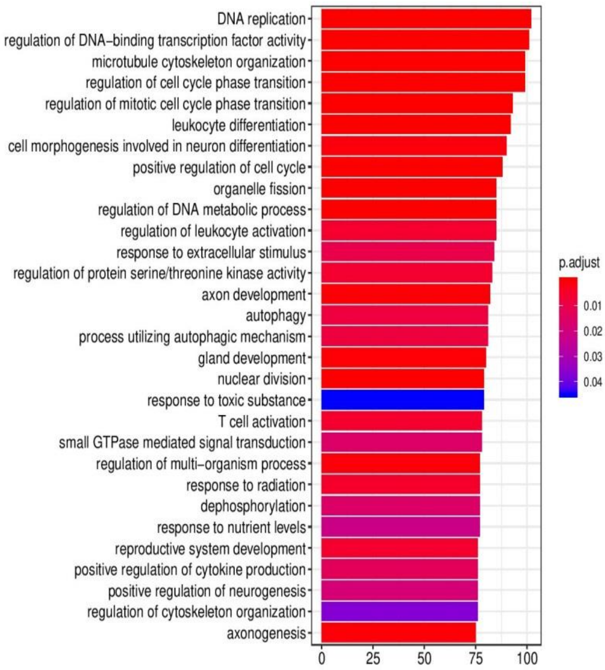

Supplementary figure 6: Pathway enrichment analysis: Pathway enrichment analysis of NT Sh UT vs NT Sh Cisplatin, CMTM6 Sh UT vs CMTM6 Sh Cisplatin

Supplementary Figure 7

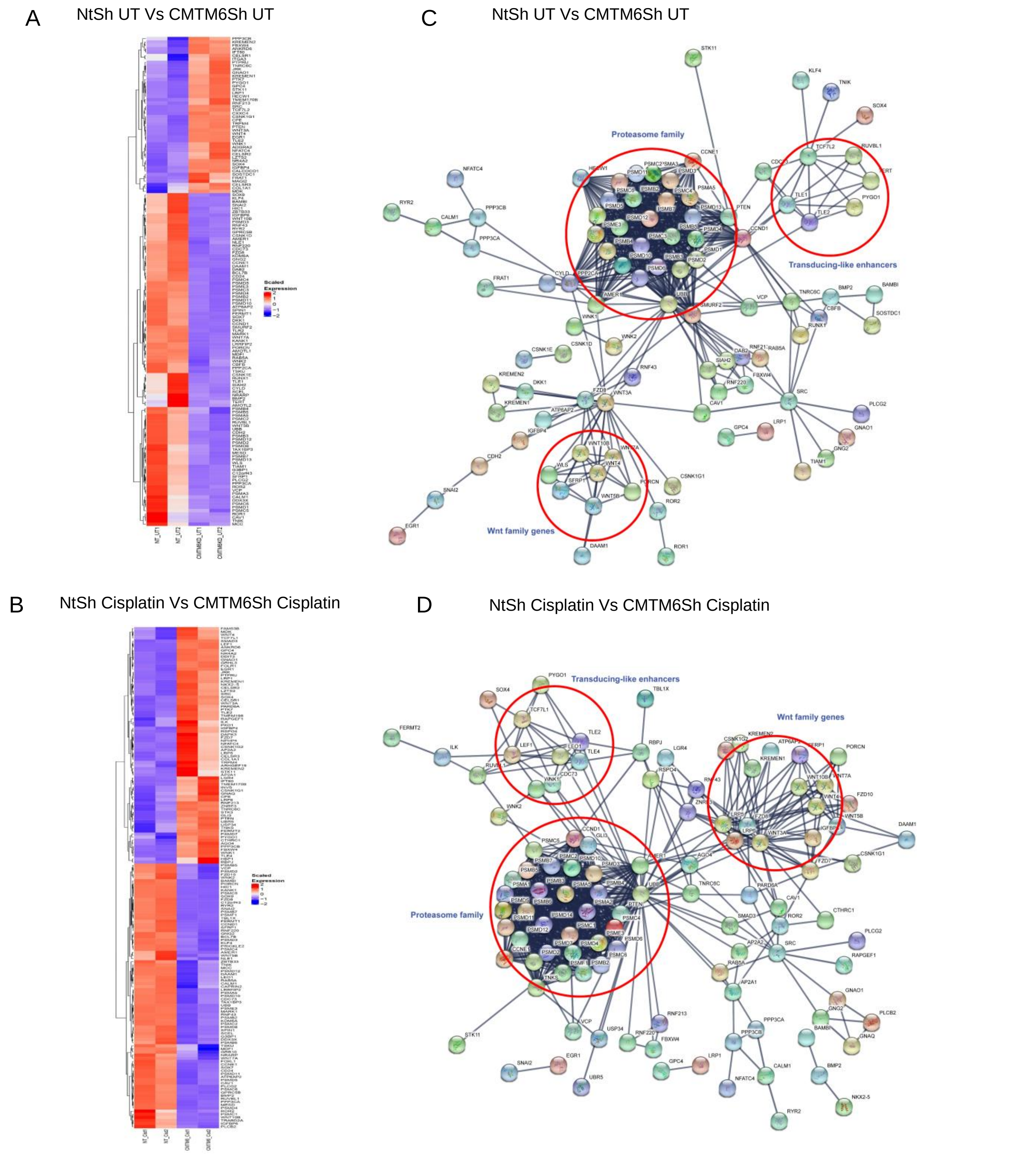

Supplementary figure 7: Wnt target genes are deregulated in CMTM6 dependent manner: A, B) Heat map of WNT signaling gene dysregulated in NT Sh UT vs CMTM6 Sh UT, NT Sh Cisplatin treated vs CMTM6 Sh Cisplatin treated. **C, D)** Network analysis of WNT pathway genes in NtSh Vs CMTM6Sh UT and NtSh Vs CMTM6Sh Cisplatin.

Supplementary Figure 8

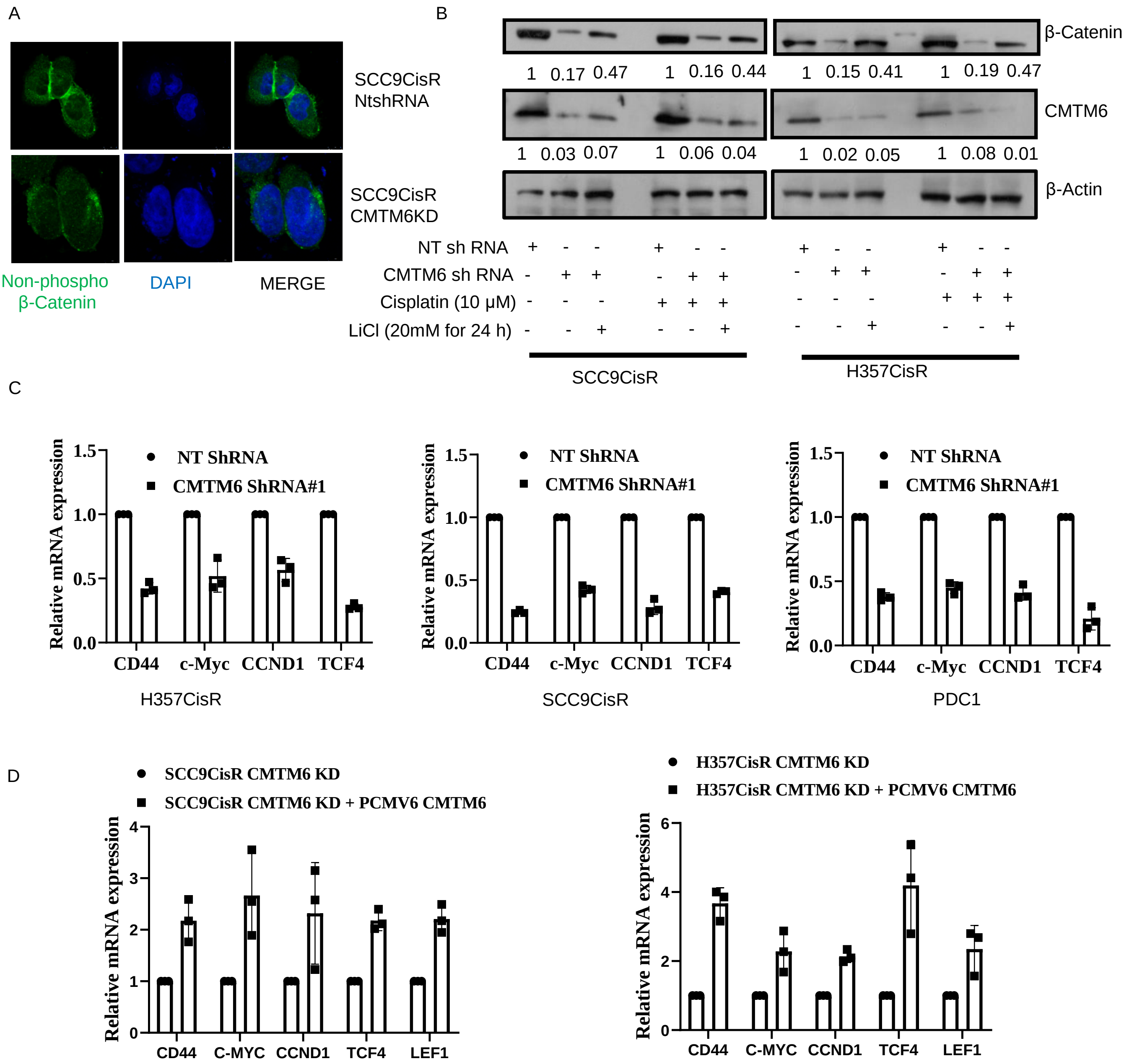

Supplementary figure 8: CMTM6 correlates with Wnt signalling : A) Confocal imaging was done with the indicated antibodies B) Lysates were isolated from indicated lines and immunoblotting (n=3) was performed with the indicated antibodies C) Relative mRNA (fold change) expression of indicated genes were analyzed by qRT PCR in indicted cells stably expressing NTShRNA or CMTM6ShRNA#1 (mean ±SEM, n=3). D) CMTM6 was overexpressed in chemoresistant CMTM6KD (ShRNA#2) and relative mRNA expression of indicated genes were determined by qRT PCR (mean ±SEM, n=3).

Supplementary Figure 9

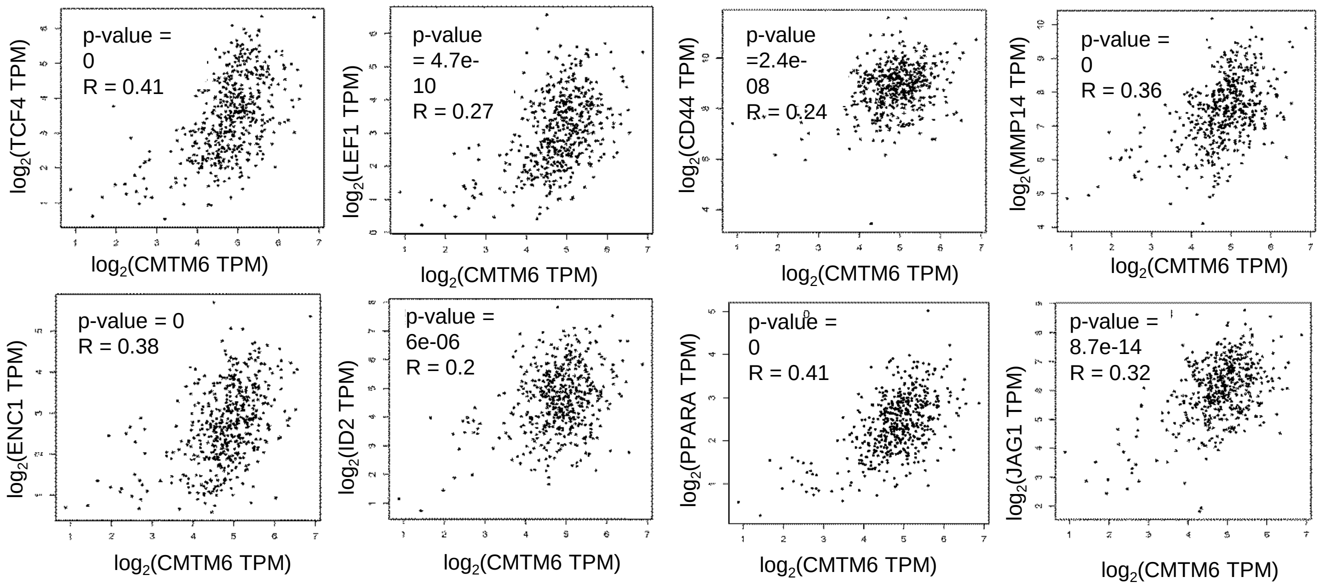

Supplementary figure 9: Expression correlation between CMTM6 and Wnt target prosurvival genes : TCF4, LEF1, CD44, MMP14, ENC1, ID2, PPARA and JAG1 RNA expression in the TCGA HNSCC database. Correlation was analyzed using Spearman's correlation coefficient test, n = 520. The analysis was performed in Gene expression profiling interactive analysis (GEPIA) platform.

Supplementary Figure 10

A

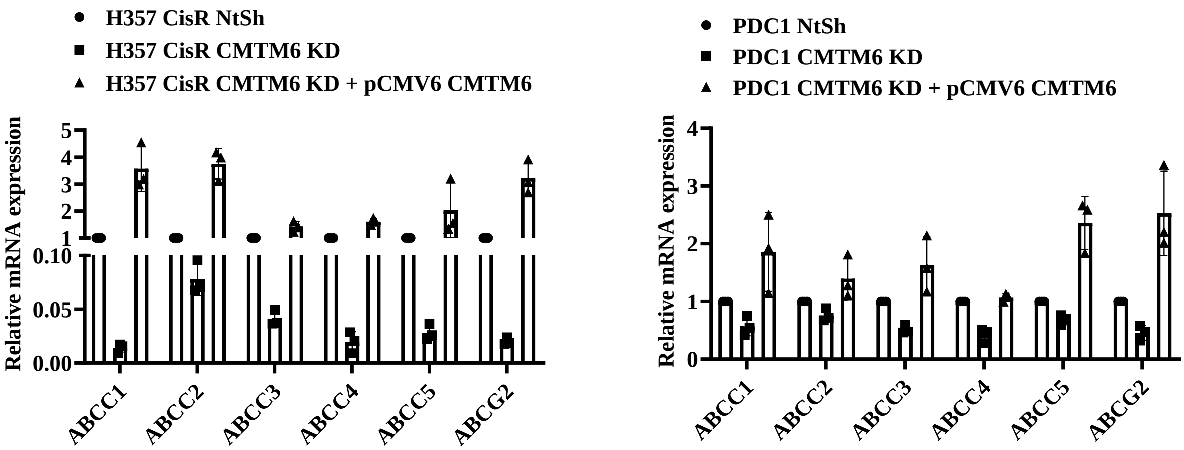

B

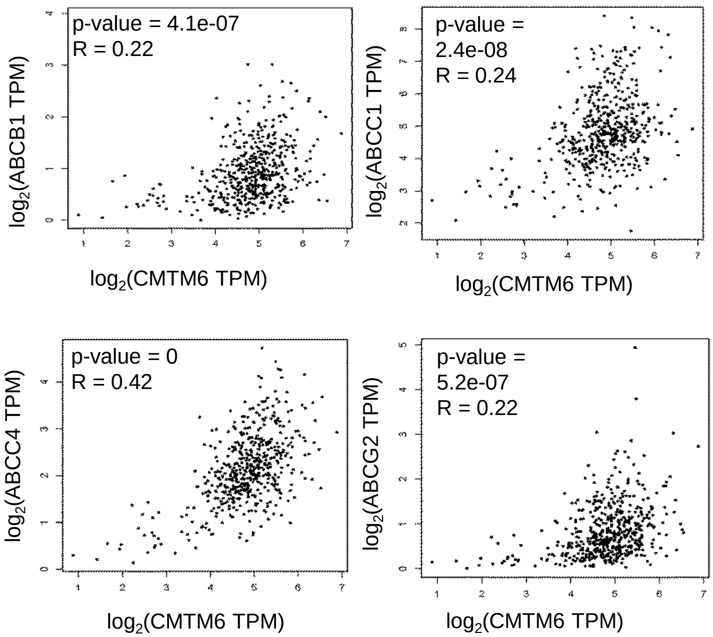

Supplementary figure 10: Correlation between CMTM6 and ABC transporters: A) CMTM6 was transiently overexpressed in chemoresistant cells stably expressing CMTM6ShRNA#2 and qRTPCR was performed for indicated genes to evaluate relative mRNA expression (mean ±SEM, n=3). B**)** Expression correlation between CMTM6 and ABC transporter genes (ABCB1, ABCC1, ABCC4 and ABCG2) mRNA in the TCGA HNSCC database. Correlation was analyzed using Spearman's correlation coefficient test, n = 520. The analysis was performed in gene expression profiling expression analysis (GEPIA) platform.

Supplemntary Table 1a

chemotherapy-responder patient details

Sl No	Tumor samples	Age/Sex	Site of disease	Clinical stage	Chemotherapy (NACT)	Cycle
1	**Patient#1**	42/M	Tongue Rt lateral border	T4aN1M0	Docetaxel + Cisplatin+ 5FU	2
2	**Patient#2**	67/M	Tongue Lt lateral border	T4aN1Mx	Docetaxel + Cisplatin+ 5FU	3
3	**Patient#3**	50/M	Rt- Buccal mucosa	T4aN2bM0	Docetaxel + Cisplatin+ 5FU	3
4	**Patient#4**	75/M	Oral cavity	T3N2bM0	Doceaqualip + Carboplatin	3
5	**Patient#5**	46/M	Tongue	T3N1M0	Docetaxel + Cisplatin+ 5FU	3
6	**Patient#6**	35/M	Tongue	T4aN2eM0	Docetaxel + Cisplatin+ 5FU	3
7	**Patient#7**	38/M	Right Buccal Mucosa	T4bN2bM0	Docetaxel + Cisplatin+ 5FU	3
8	**Patient#8**	34/M	Left Buccal Mucosa	T4aN2bMx	Docetaxel + Cisplatin+ 5FU	3
9	**Patient#9**	40/M	Tongue	T2N2cM0	Docetaxel + Cisplatin+ 5FU	3
10	**Patient#10**	45/M	Tongue	T2N1M0	Docetaxel + Cisplatin+ 5FU	3
11	**Patient#11**	51/M	Tongue Lt. lateral border	T4aN2cM0	Docetaxel + Cisplatin+ 5FU	3

Chemotherapy Doses: **Cisplatin**: 100mg. **Docetaxel**: 100mg, 5FU:1000mg, **Doceaqualip**: 80mg, **Carboplatin:** AUC 4 (area under the ROC curve)

Supplemntary Table 1b

Chemotherapy-non-responders patient Details

Sl No	Tumor samples	Age /Sex	Site of disease	Clinical stage	Chemotherapy (NACT)	Cycle
1	**Patient# 1 (PDC#1)**	76/M	Tongue Rt lateral border	T4N0M0	Paclitaxel + Cisplatin	3
2	**Patient#2**	51/M	Rt- Buccal mucosa	T2N2bM0	Docetaxel + Cisplatin+ 5FU	2
3	**Patient# 3**	60/M	Tongue Rt lateral border	T3N1M0	Paclitaxel + Cisplatin +5FU	3
4	**Patient#4**	33/M	Rt- Lower Alveolar mucosa	T3N1Mx	Docetaxel + Cisplatin	3
5	**Patient#5**	60/F	Tongue Lt lateral border	T4N0M0	Docetaxel + Cisplatin+ 5FU	3
6	**Patient#6**	59/M	Tongue Rt lateral border	T4aN1M0	Docetaxel + Cisplatin+ 5FU	3
7	**Patient#7**	46/M	Tongue	T4N3M0	Docetaxel + Cisplatin+ 5FU	3
8	**Patient#8**	55/F	Rt- Buccal Mucosa	T4aN2M0	Docetaxel + Cisplatin+ 5FU	2
9	**Patient#9**	37/M	Tongue	T4N3M0	Docetaxel + Cisplatin+ 5FU	2
10	**Patient#10**	27/M	Lt-Buccal Mucosa	T4N2M0	Docetaxel + Cisplatin+ 5FU	2
11	**Patient#11**	46/F	Rt- oral cavity	T4N1M0	Docetaxel + Cisplatin+ 5FU	3
12	**Patient#12**	42/M	Rt- Buccal Mucosa	TxN3bM0	Paclitaxel + Cisplatin+ 5FU	2
13	**Patient#13**	30/M	Tongue Rt lateral border	T2N0Mx	Paclitaxel + Cisplatin+ 5FU	3
14	**Patient#14**	52/M	Rt- Buccal Mucosa	T4N2M0	Docetaxel + Cisplatin+ 5FU	3
15	**Patient#15**	32/M	Tongue	T3N1M0	Docetaxel + Cisplatin+ 5FU	3
16	**Patient#16**	35/M	Tongue	T4aN2aM0	Docetaxel + Cisplatin+ 5FU	3
17	**Patient#17**	36/M	Left Buccal Mucosa	T4aN2aM0	Docetaxel + Cisplatin+ 5FU	3
18	**Patient #18**	38/M		T4bN2bMO	Docetaxel + Cisplatin+ 5FU	2
19	**Patient # 19**	55/M	Tongue, Left lateral border,		Docetaxel + Cisplatin+ 5FU	3
20	**Patient # 20**	35/M	Tongue	T4aN2eM0+	Docetaxel + Cisplatin+ 5FU	3
21	**Patient # 21**	36/M	Left Buccal Mucosa	cT4aN2aM0	Docetaxel + Cisplatin+ 5FU	3
22	**Patient # 22**	39/M	Right mandible	cT4bN0Mx	Docetaxel + Cisplatin+ 5FU	
23	**Patient # 23**	55/M	Tongue, Left lateral border,	cT4aN2cM0	Docetaxel + Cisplatin+ 5FU	3

chemotherapy but after 1-2 cycles became non responded.

Chemotherapy Doses: **Cisplatin**: 100mg. **Paclitaxe**l: 260 mg, **Docetaxel**: 100mg, **5FU**:1000mg **Lt-Left, Rt-Right**

Supplementary table 2

Sh RNA primers	Oligo sequence
pLKO.1 CMTM6 sh RNA F (ShRNA#1)	CCGGCTTTCTTCTGAGTCTCCTTATCTCGAGATAAGGAGACTCAGAAGAAAGTTTTTG
pLKO.1 CMTM6 sh RNA R (ShRNA#1)	AATTCAAAAACTTTCTTCTGAGTCTCCTTATCTCGAGATAAGGAGACTCAGAAGAAAG
pLKO.1 CMTM6 5' UTR sh RNA F (ShRNA#2)	CCGGCCCAAGACAGTGAAAGTAATTCTCGAGAATTACTTTCACTGTCTTGGGTTTTTG
pLKO.1 CMTM6 5' UTR sh RNA R (ShRNA#2)	AATTCAAAAACCCAAGACAGTGAAAGTAATTCTCGAGAATTACTTTCACTGTCTTGGG

qRT PCR Primers	Primer sequence
CMTM6 qRT F	CGCTGCCTACTTTTTCATGG
CMTM6 qRT R	GAAGAAAGGCACTGCAGCTT
18S qRT F	GTAACCCGTTGAACCCCATT
18S qRT R	CCATCCAATCGGTAGTAGCG
GAPDH qRT F	TCGGAGTCAACGGATTTGGT
GAPDH qRT R	TTGCCATGGGTGGAATCATA
OCT4 qRT F	CGACCATCTGCCGCTTTGAG
OCT4 qRT R	CCCCCTGTCCCCCATTCCTA
SOX2 qRT F	CACCTACAGCATGTCCTACTC
SOX2 qRT R	CATGCTGTTTCTTACTCTCCTC
Nanog qRT F	CAACTGGCCGAAGAATAGCA
Nanog qRT R	GCAGGAGAATTTGGCTGGAA
CD44 qRT F	TGGCACCCGCTATGTCCAG
CD44 qRT R	GTAGCAGGGATTCTGTCTG
LEF1 qRT F	ATCAAGTCTTCCTTGGTGAA
LEF1 qRT R	TATGTACCCGGAATAACTCG
TCF4 qRT F	AGAGCGACAAGCCCCAGAC
TCF4 qRT R	ATTCGCTGCGTCTCCCATC
CCND1 qRT F	TGTGAAGTTCATTTCCAATCC
CCND1 qRT R	GTCACACTTGATCACTCTGG
c-Myc F	CCTGGTGCTCCATGAGGAGAC
c-Myc R	CAGACTCTGACCTTTTGCCAG

Supplementary Table 3

Accession	Description	Score	Coverage	# Proteins	Unique Peptid	# Peptides	# PSMs	114/115	113/115	116/115	117/115	118/115	119/115	121/115	# AAs	MW [kDa]	calc. pI
B4DVQ0	cDNA FLJ58286, highly similar to Actin, cytoplasmic 2 OS=H	604.42	33.93	64	2	12	273	1.243	1.102	1.372	1.446	1.321	1.663	1.433	333.000	37.325	5.707
Q5T8M8	Actin, alpha skeletal muscle OS=Homo sapiens GN=ACTA1	596.32	35.19	40	2	12	271	1.977	1.217	1.596	2.029	1.748	1.836	1.432	287.000	32.028	5.415
	>sp|TRY1_BOVIN|	232.42	33.33	7	6	7	120	1.422	1.114	1.388	1.480	1.430	1.630	1.354	243	25.4	7.91
P02545	Prelamin-A/C OS=Homo sapiens GN=LMNA PE=1 SV=1 - [L	171.62	40.36	11	23	23	71	1.205	1.138	1.176	1.394	1.201	1.338	1.154	664	74.1	7.02
P07355	Annexin A2 OS=Homo sapiens GN=ANXA2 PE=1 SV=2 - [A	116.60	44.84	26	17	17	47	1.433	1.214	1.357	1.395	1.541	1.624	1.430	339	38.6	7.75
C9JFR7	Cytochrome c (Fragment) OS=Homo sapiens GN=CYCS PE=	102.40	49.50	5	5	5	43	1.714	1.157	1.722	1.927	1.574	1.746	1.504	101	11.3	9.66
P11021	78 kDa glucose-regulated protein OS=Homo sapiens GN=H	98.37	32.57	7	15	17	48	1.515	1.126	1.389	1.733	1.415	1.737	1.314	654	72.3	5.16
B3GQS7	Mitochondrial heat shock 60kD protein 1 variant 1 OS=Hom	80.24	35.68	18	18	18	41	1.732	1.131	1.712	1.911	1.603	1.701	1.523	569	60.6	6.04
Q0QF37	Malate dehydrogenase (Fragment) OS=Homo sapiens GN=I	73.67	34.43	8	9	9	41	1.529	1.121	1.471	1.644	1.364	1.651	1.430	305	31.9	7.88
I3L2P8	Protein disulfide-isomerase OS=Homo sapiens GN=P4HB PE	60.31	17.78	18	8	8	32	1.525	1.043	1.468	1.736	1.512	1.773	1.389	450	51.0	4.91
Q9BTI9	NPM1 protein (Fragment) OS=Homo sapiens GN=NPM1 PE=	57.32	31.14	13	7	7	21	1.892	1.199	1.950	2.088	1.817	2.056	1.740	228	25.0	4.86
B3KML9	cDNA FLJ11352 fis, clone HEMBA1000020, highly similar to	52.64	14.61	61	5	5	22	2.024	1.117	1.865	2.302	1.732	1.987	1.476	397	44.6	4.93
Q53G71	Calreticulin variant (Fragment) OS=Homo sapiens PE=2 SV=	51.75	10.59	6	5	5	29	1.558	1.114	1.664	1.822	1.617	1.780	1.474	406	46.9	4.45
P25705	ATP synthase subunit alpha, mitochondrial OS=Homo sapier	50.90	24.59	12	12	12	17	1.642	1.113	1.569	1.826	1.553	1.799	1.484	553	59.7	9.13
B7Z4V2	cDNA FLJ51907, highly similar to Stress-70 protein, mitocho	49.97	14.59	10	8	8	34	1.570	1.058	1.654	1.883	1.639	1.781	1.542	665	72.4	5.94
B3KQT9	cDNA PSEC0175 fis, clone OVARC1000169, highly similar to	48.28	22.29	6	8	11	26	1.618	1.140	1.605	1.816	1.574	1.808	1.532	480	54.1	7.21
Q96KK5	Histone H2A type 1-H OS=Homo sapiens GN=HIST1H2AH F	46.82	27.34	22	3	3	21	1.209	1.154	1.151	1.383	1.341	1.555	1.282	128	13.9	10.89
F8VVB9	Tubulin alpha-1B chain (Fragment) OS=Homo sapiens GN=T	46.39	11.34	37	2	2	15	2.295	1.249	2.145	3.028	1.996	2.492	1.811	247	27.5	5.20
Q5CAQ5	Tumor rejection antigen (Gp96) 1 OS=Homo sapiens GN=T	42.15	11.47	9	10	10	20	1.634	1.110	1.612	1.838	1.620	1.878	1.526	802	92.3	4.86
F8VPV9	ATP synthase subunit beta OS=Homo sapiens GN=ATP5B P	39.23	27.03	9	10	10	16	1.521	1.048	1.412	1.562	1.519	1.750	1.414	518	55.3	5.40
B4DMA2	cDNA FLJ54023, highly similar to Heat shock protein HSP 90	38.89	7.29	15	3	6	22	2.387	1.165	2.321	2.937	2.016	2.311	1.879	686	79.1	5.02
B4E2Z3	cDNA FLJ54090, highly similar to 4F2 cell-surface antigen he	38.57	14.48	9	5	5	13	1.613	1.122	1.534	1.786	1.471	1.680	1.344	511	55.9	5.17
P26599	Polypyrimidine tract-binding protein 1 OS=Homo sapiens GN	38.11	10.55	13	5	5	18	1.427	1.073	1.404	1.592	1.345	1.526	1.289	531	57.2	9.17
P63104	14-3-3 protein zeta/delta OS=Homo sapiens GN=YWHAZ PE	37.65	33.06	34	3	6	19	1.867	0.954	1.570	1.919	1.655	1.972	1.658	245	27.7	4.79
Q53HF2	Heat shock 70kDa protein 8 isoform 2 variant (Fragment) O	36.23	15.82	40	4	7	19	2.000	1.101	1.973	2.186	1.836	2.000	1.748	493	53.5	5.86
Q2VPJ6	HSP90AA1 protein (Fragment) OS=Homo sapiens GN=HSP9	35.11	8.03	15	2	5	19	1.199	1.056	0.978	1.397	1.163	1.393	0.930	585	68.3	5.19
P04083	Annexin A1 OS=Homo sapiens GN=ANXA1 PE=1 SV=2 - [A	33.38	23.99	5	6	6	11	2.445	1.121	2.321	3.167	2.082	2.112	1.922	346	38.7	7.02
P62424	60S ribosomal protein L7a OS=Homo sapiens GN=RPL7A PE	32.79	12.03	4	3	3	10	1.606	1.148	1.679	1.830	1.639	1.803	1.509	266	30.0	10.61
B2R984	cDNA, FLJ94268, highly similar to Homo sapiens histone 1,	32.50	26.48	8	5	7	19	1.754	1.243	1.720	2.008	1.806	1.904	1.552	219	21.9	11.03
B3KUZ8	Aspartate aminotransferase OS=Homo sapiens PE=2 SV=1	30.59	16.98	5	5	5	9	1.684	1.116	1.662	1.905	1.679	1.830	1.585	371	41.3	8.84
E7EUT5	Glyceraldehyde-3-phosphate dehydrogenase OS=Homo sap	30.42	22.31	10	5	5	22	2.070	1.129	2.102	2.598	1.912	2.141	1.715	260	27.9	6.95
H6VRG2	Keratin 1 OS=Homo sapiens GN=KRT1 PE=3 SV=1 - [H6VR	28.67	8.70	8	3	5	12	1.732	1.161	1.624	1.801	1.904	3.017	1.626	644	66.0	8.12
P23284	Peptidyl-prolyl cis-trans isomerase B OS=Homo sapiens GN=	28.58	29.63	9	5	6	19	1.615	1.091	1.523	1.706	1.494	1.612	1.368	216	23.7	9.41
P22626	Heterogeneous nuclear ribonucleoproteins A2/B1 OS=Homo	28.24	19.55	2	6	6	12	1.227	1.155	1.165	1.301	1.123	1.188	1.237	353	37.4	8.95
B4DEI3	cDNA FLJ57715, highly similar to Voltage-dependent anion-s	28.21	17.95	4	2	2	13	1.824	1.137	1.795	2.202	1.862	2.089	1.656	156	16.7	9.39
B4DFG4	cDNA FLJ58801, highly similar to Interleukin enhancer-bindi	27.45	5.14	10	3	3	20	1.718	1.188	1.584	1.854	1.688	1.842	1.441	506	54.7	8.81
Q9UNM1	Chaperonin 10-related protein (Fragment) OS=Homo sapien	26.40	55.67	4	5	5	12	1.526	1.091	1.431	1.549	1.452	1.627	1.319	97	10.3	9.00
P49411	Elongation factor Tu, mitochondrial OS=Homo sapiens GN=T	25.01	12.17	2	5	5	9	1.530	1.114	1.573	1.805	1.560	1.696	1.503	452	49.5	7.61
B4DNX1	cDNA FLJ53752, highly similar to Heat shock 70 kDa protein	24.41	11.03	26	1	4	9	1.902	1.034	2.327	2.703	2.092	2.412	1.769	417	45.1	5.73
B4DE78	cDNA FLJ52141, highly similar to 14-3-3 protein gamma OS	24.36	28.50	24	2	5	17	1.467	1.063	1.265	1.427	1.132	1.500	0.980	207	23.5	4.82
	>sp|GSTP1_HUMAN|	24.17	14.83	3	1	2	20	2.453	1.112	2.398	3.158	1.758	1.846	2.127	209	23.2	5.64
P27824	Calnexin OS=Homo sapiens GN=CANX PE=1 SV=2 - [CALX_	22.75	7.09	14	4	4	10	1.729	1.113	1.593	1.963	1.639	1.753	1.547	592	67.5	4.60
Q09666	Neuroblast differentiation-associated protein AHNAK OS=Ho	22.16	10.54	5	10	10	13	1.499	1.163	1.490	1.582	1.633	1.978	1.574	5890	628.7	6.15
Q04695	Keratin, type I cytoskeletal 17 OS=Homo sapiens GN=KRT1	22.10	18.98	44	1	7	7	1.275	1.011	1.375	1.672	1.305	1.589	1.347	432	48.1	5.02
D6RF44	Heterogeneous nuclear ribonucleoprotein D0 (Fragment) OS	21.84	19.64	12	2	2	30	1.567	1.162	1.663	1.768	1.661	1.646	1.494	112	12.6	8.57
P62258	14-3-3 protein epsilon OS=Homo sapiens GN=YWHAE PE=1	21.16	14.12	23	1	4	11	1.551	0.819	1.154	1.509	1.156	1.188	1.240	255	29.2	4.74
P31947	14-3-3 protein sigma OS=Homo sapiens GN=SFN PE=1 SV=	21.16	19.76	21	1	4	10								248	27.8	4.74
P27348	14-3-3 protein theta OS=Homo sapiens GN=YWHAQ PE=1	21.16	15.92	23	1	4	10	2.201	1.235	2.527	2.995	1.745	2.548	2.320	245	27.7	4.78
B5BU24	14-3-3 protein beta/alpha OS=Homo sapiens GN=YWHAB P	21.16	19.11	22	1	4	15	1.218	1.095	1.259	1.610	1.359	1.632	1.257	246	28.1	4.83
E9PFF2	Transketolase OS=Homo sapiens GN=TKT PE=2 SV=1 - [E9	20.86	13.47	7	3	3	10	2.411	1.126	2.471	2.663	2.118	2.461	1.963	334	36.4	7.77
G3V576	Heterogeneous nuclear ribonucleoproteins C1/C2 OS=Homo	19.76	15.58	46	4	4	9	1.272	1.125	1.299	1.479	1.404	1.526	1.406	231	25.2	9.82
P09211	Glutathione S-transferase P OS=Homo sapiens GN=GSTP1 F	19.08	15.24	5	1	2	18	1.852	1.006	1.934	2.404	1.460	1.666	1.151	210	23.3	5.64
C9JW96	Prohibitin (Fragment) OS=Homo sapiens GN=PHB PE=2 SV	18.96	32.52	11	7	7	15	1.554	1.059	1.627	1.878	1.663	1.742	1.449	246	26.9	5.40
Q15149	Plectin OS=Homo sapiens GN=PLEC PE=1 SV=3 - [PLEC_HU	18.64	2.07	6	4	6	10	1.541	1.136	1.401	1.748	1.485	1.629	1.362	4684	531.5	5.96
B3KTN4	Citrate synthase OS=Homo sapiens PE=2 SV=1 - [B3KTN4_	18.37	9.50	12	4	4	9	1.696	1.193	1.645	1.858	1.698	1.780	1.542	421	47.0	7.24
B7Z254	Protein disulfide-isomerase A6 OS=Homo sapiens GN=PDIA	18.06	9.84	6	3	3	6	1.621	1.302	1.574	1.873	1.616	1.760	1.454	437	47.8	5.08
F5GWP8	Junction plakoglobin OS=Homo sapiens GN=JUP PE=2 SV=	17.82	12.69	38	1	6	6	1.479	1.028	1.602	2.054	1.632	1.905	1.599	591	66.3	5.19
B3KRY5	cDNA FLJ35087 fis, clone PLACE6005546, highly similar to F	17.71	7.83	4	2	2	9	1.817	1.202	1.827	1.774	1.647	1.880	1.552	345	39.0	6.51
Q7Z612	Acidic ribosomal phosphoprotein P1 OS=Homo sapiens PE=	17.60	14.16	4	1	1	7	1.666	1.006	1.722	2.030	1.592	1.859	1.583	113	11.4	4.36
P16401	Histone H1.5 OS=Homo sapiens GN=HIST1H1B PE=1 SV=3	16.92	12.39	3	1	3	7	1.752	1.074	1.753	2.046	1.804	2.088	1.601	226	22.6	10.92
B4E241	Serine/arginine-rich-splicing factor 3 OS=Homo sapiens GN=	16.43	15.32	2	2	2	7	1.539	1.057	1.345	1.470	1.370	1.659	1.348	124	14.2	10.08
Q02539	Histone H1.1 OS=Homo sapiens GN=HIST1H1A PE=1 SV=3	16.36	12.09	3	1	3	7	1.919	1.128	1.794	2.318	1.870	2.312	1.847	215	21.8	10.99
P35908	Keratin, type II cytoskeletal 2 epidermal OS=Homo sapiens	16.21	9.08	26	2	5	6	1.539	1.241	1.404	1.548	1.596	1.770	1.236	639	65.4	8.00
P06733	Alpha-enolase OS=Homo sapiens GN=ENO1 PE=1 SV=2 - [	15.10	7.83	7	3	3	10	1.749	1.068	1.433	2.110	1.689	1.818	1.760	434	47.1	7.39
K7EMV3	Histone H3 OS=Homo sapiens GN=H3F3B PE=3 SV=1 - [K7	15.00	17.39	15	2	2	6	1.448	1.139	1.344	1.502	1.394	1.494	1.303	92	10.3	11.82

A8K4W7	cDNA FLJ76284, highly similar to Homo sapiens succinate-C	14.91	8.11	5	2	2	4	1.796	1.166	1.699	2.085	1.706	1.879	1.656	333	35.0	8.79
B4DUI5	Triosephosphate isomerase OS=Homo sapiens PE=2 SV=1 -	14.39	20.19	4	3	3	5	2.001	1.163	2.461	2.491	1.758	1.966	1.608	213	22.9	6.92
Q8IUB0	Elongation factor 1-alpha OS=Homo sapiens PE=2 SV=1 - [	14.28	8.86	34	3	3	5	1.730	1.123	1.638	2.015	1.512	1.625	1.465	361	38.6	8.85
B4E0N9	Glutamate dehydrogenase OS=Homo sapiens PE=2 SV=1 -	14.21	10.82	13	5	5	6	1.459	1.116	1.571	1.726	1.531	1.688	1.470	490	54.2	7.58
Q562M3	Actin-like protein (Fragment) OS=Homo sapiens GN=ACT PI	13.84	17.48	1	1	1	6	1.606	1.104	1.581	1.965	1.540	1.799	1.427	103	11.5	6.68
P13639	Elongation factor 2 OS=Homo sapiens GN=EEF2 PE=1 SV=	13.61	3.15	16	3	3	5	2.271	1.135	1.996	2.781	2.012	2.245	1.786	858	95.3	6.83
B3KTP9	cDNA FLJ38578 fis, clone HCHON2007674, highly similar to	13.15	5.37	10	5	5	5	1.780	1.090	2.140	2.274	1.880	2.209	1.964	633	68.4	4.65
F8VVM2	Phosphate carrier protein, mitochondrial OS=Homo sapiens	12.89	8.33	5	3	3	4	1.666	1.135	1.532	1.774	1.569	1.697	1.463	324	36.1	9.26
F8W1A4	Adenylate kinase 2, mitochondrial OS=Homo sapiens GN=A	12.69	23.28	7	4	4	6	1.905	1.132	1.906	2.133	1.782	1.872	1.593	232	25.6	7.83
P40939	Trifunctional enzyme subunit alpha, mitochondrial OS=Hom	12.56	9.96	6	5	5	6	1.582	1.183	1.305	1.908	1.615	1.781	1.490	763	82.9	9.04
H7BZJ3	Thioredoxin (Fragment) OS=Homo sapiens GN=PDIA3 PE=	12.35	34.15	1	1	4	6	1.434	0.963	1.436	1.722	1.575	1.714	1.404	123	13.5	7.30
F8VPE8	60S acidic ribosomal protein P0 (Fragment) OS=Homo sapie	11.94	7.84	12	1	1	11	1.569	1.007	1.528	1.765	1.432	1.598	1.423	153	16.7	9.33
B4DIW2	cDNA FLJ54035, highly similar to Neutral alpha-glucosidase	11.87	4.10	9	3	3	9	1.857	1.156	1.790	2.049	1.672	1.950	1.549	830	93.9	5.87
B4E1T1	cDNA FLJ54081, highly similar to Keratin, type II cytoskelet	11.87	8.11	21	2	4	4	1.237	1.126	1.218	1.473	1.190	1.595	1.240	555	58.8	5.97
P58107	Epiplakin OS=Homo sapiens GN=EPPK1 PE=1 SV=2 - [EPIP	11.84	6.35	3	2	4	16	1.019	1.200	0.996	1.262	1.072	1.136	0.897	5090	555.3	5.60
D6REM6	Matrin-3 OS=Homo sapiens GN=MATR3 PE=2 SV=1 - [D6R	11.45	3.78	16	3	3	5	1.552	1.121	1.540	1.622	1.507	1.659	1.466	794	88.3	5.76
A8K9J7	Histone H2B OS=Homo sapiens PE=2 SV=1 - [A8K9J7_HUM	11.28	12.70	20	2	2	5	1.518	1.211	1.404	1.741	1.347	1.728	1.307	126	14.0	10.32
M0QYT0	Uncharacterized protein (Fragment) OS=Homo sapiens PE=	11.22	4.36	5	1	1	16	1.518	1.064	1.467	1.628	1.483	1.766	1.454	321	36.0	7.42
D6R904	Tropomyosin alpha-3 chain OS=Homo sapiens GN=TPM3 PI	11.20	20.00	46	2	2	3	1.308	1.143	1.300	1.348	1.349	1.507	1.330	95	11.0	4.79
B4DMD1	cDNA FLJ53360, highly similar to Heterogeneous nuclear rib	10.87	6.98	15	3	3	3	1.464	1.224	1.435	1.637	1.617	1.849	1.397	473	52.9	9.32
F8VUA6	60S ribosomal protein L18 (Fragment) OS=Homo sapiens G	10.41	7.69	9	1	1	6	1.560	0.976	1.383	1.482	1.405	1.592	1.225	130	14.5	11.75
Q9Y5B2	Junction adhesion molecule OS=Homo sapiens PE=2 SV=1	10.01	13.13	4	2	2	6	1.589	1.189	1.544	1.623	1.545	1.735	1.463	259	28.1	8.29
B4DUQ1	cDNA FLJ54552, highly similar to Heterogeneous nuclear rib	9.92	14.58	9	4	4	5	1.721	1.043	1.351	1.637	1.443	1.591	1.287	439	48.5	5.92
Q96AG4	Leucine-rich repeat-containing protein 59 OS=Homo sapiens	9.84	3.91	1	1	1	5	1.535	1.124	1.524	1.660	1.484	1.637	1.502	307	34.9	9.57
P84090	Enhancer of rudimentary homolog OS=Homo sapiens GN=E	9.62	5.77	1	1	1	5	1.356	1.076	1.382	1.505	1.449	1.600	1.324	104	12.3	5.92
B4DSR0	cDNA FLJ60080, highly similar to 130 kDa leucine-rich prote	9.51	1.38	2	2	2	4	1.572	1.079	1.559	1.723	1.534	1.779	1.609	1087	123.2	5.54
H7C469	Uncharacterized protein (Fragment) OS=Homo sapiens PE=	9.35	7.12	6	2	2	3	1.347	1.246	1.233	1.474	1.347	1.370	1.224	379	40.4	5.76
Q8WUW7	Pyruvate kinase (Fragment) OS=Homo sapiens GN=PKM2 P	9.29	9.04	8	3	3	5	1.941	1.212	1.895	2.292	1.866	2.071	1.633	343	37.3	8.22
A8K787	cDNA FLJ75273, highly similar to Homo sapiens solute carrie	9.13	7.38	10	3	3	4	1.407	1.157	1.515	1.707	1.554	1.746	1.511	298	33.1	9.76
H7C144	Alpha-actinin-4 (Fragment) OS=Homo sapiens GN=ACTN4 F	9.10	11.99	18	3	3	6	1.781	1.093	1.598	1.928	1.521	1.710	1.338	342	39.0	5.24
B4DM82	cDNA FLJ53060, moderately similar to Peptidyl-prolyl cis-tra	9.02	10.08	20	1	2	7	2.030	1.057	2.312	3.028	2.145	2.464	2.034	129	14.1	8.47
Q7KZJ3	Catenin (Cadherin-associated protein), delta 1 OS=Homo sa	8.88	5.90	6	3	3	3	1.715	0.948	1.663	1.839	1.339	1.632	1.463	610	68.0	8.13
H0YEX5	Splicing factor 3B subunit 2 (Fragment) OS=Homo sapiens (	8.84	7.62	4	2	2	3	1.518	1.053	1.553	1.667	1.544	1.732	1.573	315	35.0	4.81
Q8N1C0	CTNNA1 protein OS=Homo sapiens GN=CTNNA1 PE=2 SV=	8.82	9.33	11	3	4	4	1.554	1.151	1.489	1.750	1.534	1.852	1.500	536	59.5	5.60
P13667	Protein disulfide-isomerase A4 OS=Homo sapiens GN=PDIA	8.66	5.27	1	4	4	7	1.701	1.062	1.590	1.775	1.632	1.839	1.444	645	72.9	5.07
A8KA83	cDNA FLJ78586, highly similar to Homo sapiens VAMP (vesi	8.54	14.05	2	2	2	8	1.502	1.075	1.352	1.685	1.495	1.552	1.359	242	27.3	8.62
H0YDD8	60S acidic ribosomal protein P2 (Fragment) OS=Homo sapie	8.38	48.91	2	2	2	4	1.819	1.001	1.823	1.787	1.549	1.761	1.573	92	9.1	4.46
M0R0N3	Heterogeneous nuclear ribonucleoprotein M (Fragment) OS=	8.21	20.65	10	2	2	3	1.598	1.276	1.444	1.531	1.272	1.632	1.352	276	30.1	8.72
A2T926	Thymopentin OS=Homo sapiens GN=TMPO PE=2 SV=1 - [A	7.93	16.53	6	3	3	6	1.464	1.130	1.430	1.491	1.378	1.615	1.293	248	27.4	8.48
Q08211	ATP-dependent RNA helicase A OS=Homo sapiens GN=DHX	7.91	2.36	2	2	2	4	1.519	1.367	1.434	1.848	1.445	1.530	1.325	1270	140.9	6.84
O95678	Keratin, type II cytoskeletal 75 OS=Homo sapiens GN=KRT	7.52	5.44	21	1	3	3	1.347	0.887	1.236	1.648	1.372	1.560	1.182	551	59.5	7.74
H0YE40	CD44 antigen (Fragment) OS=Homo sapiens GN=CD44 PE=	7.36	35.37	9	2	2	3	1.443	1.027	1.558	1.958	1.517	1.706	1.283	82	9.1	8.16
P62805	Histone H4 OS=Homo sapiens GN=HIST1H4A PE=1 SV=2 -	7.27	19.42	3	2	2	2	1.660	1.252	1.481	1.869	1.527	1.797	1.504	103	11.4	11.36
B4DHG0	Dihydrolipoyl dehydrogenase, mitochondrial OS=Homo sapi	7.17	7.32	6	3	3	3	1.802	1.129	1.753	1.820	1.704	1.786	1.587	410	43.6	7.03
D6R9B6	40S ribosomal protein S3a OS=Homo sapiens GN=RPS3A PI	7.16	8.28	5	1	1	4	1.119	0.731	0.786	1.029	0.938	0.864	0.902	145	16.5	9.25
P13645	Keratin, type I cytoskeletal 10 OS=Homo sapiens GN=KRT1	7.09	6.68	29	2	4	4	1.395	1.137	1.389	1.714	1.695	2.254	1.297	584	58.8	5.21
Q96Q74	Mitochondrial ribosomal protein L7/L12 (Fragment) OS=Hon	6.99	42.37	3	2	2	3	1.275	1.181	1.250	1.441	1.263	1.475	1.390	59	6.4	9.04
P37802	Transgelin-2 OS=Homo sapiens GN=TAGLN2 PE=1 SV=3 -	6.81	6.03	1	1	1	2	1.939	1.124	1.657	2.228	1.647	1.994	1.540	199	22.4	8.25
B4DY09	cDNA FLJ51660, highly similar to Interleukin enhancer-bindi	6.51	3.13	4	1	1	2	1.332	1.150	1.233	1.425	1.305	1.471	1.340	352	38.9	4.94
E7BSV0	Epidermal growth factor receptor variant A OS=Homo sapie	6.42	1.67	22	2	2	2	1.921	1.241	1.893	2.277	1.915	2.208	1.806	1136	125.7	6.65
P14314	Glucosidase 2 subunit beta OS=Homo sapiens GN=PRKCSH	6.22	5.68	7	3	3	3	1.366	0.985	1.362	1.725	1.349	1.564	1.358	528	59.4	4.41
Q96HX3	Similar to ribophorin I (Fragment) OS=Homo sapiens PE=2	6.17	6.69	8	3	3	3	1.434	1.122	1.321	1.440	1.284	1.495	1.220	568	64.5	6.55
H7BY36	RNA-binding protein EWS (Fragment) OS=Homo sapiens GN	6.15	4.55	4	1	1	2	1.214	0.830	1.244	1.406	1.391	1.592	1.141	308	32.2	9.82
F8W8J4	Myoferlin OS=Homo sapiens GN=MYOF PE=2 SV=1 - [F8W	5.91	1.12	3	2	2	6	1.664	1.066	1.571	1.773	1.574	1.848	1.460	2061	234.5	6.18
B7Z5V2	cDNA FLJ54141, highly similar to Ezrin OS=Homo sapiens P	5.88	5.96	24	4	4	4	1.841	1.067	1.716	2.248	1.679	1.944	1.648	554	65.5	5.91
Q01650	Large neutral amino acids transporter small subunit 1 OS=H	5.88	5.33	3	2	2	3	1.211	1.167	1.374	1.708	1.128	1.712	1.257	507	55.0	7.72
B4DG62	cDNA FLJ56506, highly similar to Hexokinase-1 (EC 2.7.1.1)	5.73	1.97	7	2	2	2	1.576	0.975	1.433	1.648	1.470	1.558	1.401	915	102.2	6.80
Q5VXV2	Protein SET OS=Homo sapiens GN=SET PE=2 SV=2 - [Q5V	5.67	6.72	3	2	2	2	3.394	1.260	3.207	3.694	2.567	3.031	2.477	268	31.3	4.21
E7EN95	Filamin-B OS=Homo sapiens GN=FLNB PE=2 SV=1 - [E7EN	5.66	1.08	3	2	2	4	1.522	1.149	1.383	1.465	1.487	1.609	1.387	2409	256.1	5.73
Q13751	Laminin subunit beta-3 OS=Homo sapiens GN=LAMB3 PE=	5.65	2.90	2	2	2	2	1.004	0.743	1.023	1.081	0.982	1.136	0.888	1172	129.5	7.21
B2R4P2	cDNA, FLJ92164, highly similar to Homo sapiens peroxiredo	5.57	10.05	4	2	2	2	2.346	1.173	2.513	3.179	2.099	2.536	2.020	199	22.2	8.38
Q96T67	TOB3 OS=Homo sapiens PE=2 SV=1 - [Q96T67_HUMAN]	5.54	3.98	3	1	1	2	0.916	1.034	1.057	1.186	1.194	1.545	1.405	578	65.1	9.33
P46783	40S ribosomal protein S10 OS=Homo sapiens GN=RPS10 PI	5.44	5.45	4	1	1	2	2.237	1.225	1.919	2.296	1.954	2.485	2.243	165	18.9	10.15
A6XGL3	Protease serine 1 OS=Homo sapiens PE=2 SV=1 - [A6XGL3	5.33	7.59	19	1	2	5	1.575	1.199	1.403	1.277	1.415	1.657	1.488	237	25.4	7.55
P26232	Catenin alpha-2 OS=Homo sapiens GN=CTNNA2 PE=1 SV=	5.32	3.67	5	1	2	2	1.365	0.830	1.412	1.743	1.166	1.698	1.138	953	105.2	5.71
B4E091	cDNA FLJ55438, highly similar to Splicing factor 3 subunit 1	5.16	2.32	2	1	1	4	1.214	1.217	0.988	1.076	1.115	1.392	1.086	690	77.4	5.16
P80723	Brain acid soluble protein 1 OS=Homo sapiens GN=BASP1 F	5.16	6.17	1	1	1	2	1.828	1.593	1.673	2.030	1.627	1.764	1.610	227	22.7	4.63

B4DJE3	cDNA FLJ52929, highly similar to Dolichyl-diphosphooligosac	5.14	2.15	4	1	1	2	1.454	1.034	1.486	1.673	1.450	1.665	1.426	419	46.4	7.06
Q15365	Poly(rC)-binding protein 1 OS=Homo sapiens GN=PCBP1 PE	5.01	9.27	16	2	2	2	1.345	1.019	1.378	1.539	1.375	1.665	1.292	356	37.5	7.09
B7Z3V1	cDNA FLJ60077, highly similar to Sodium/potassium-transpo	4.97	1.96	7	2	2	2	1.435	0.935	1.492	1.821	1.181	1.420	1.270	1020	112.4	5.35
E5RK64	Vesicle-associated membrane protein-associated protein B/C	4.97	19.72	3	1	1	3	1.712	1.114	1.757	1.826	1.639	1.903	1.669	71	7.8	9.42
Q59FZ0	LAMP1 protein variant (Fragment) OS=Homo sapiens PE=2	4.97	2.30	3	1	1	2	1.271	1.049	1.210	1.435	1.306	1.362	1.286	392	41.8	7.87
F8WBR5	Calmodulin OS=Homo sapiens GN=CALM2 PE=2 SV=1 - [F8	4.83	10.77	14	1	1	2	1.804	1.298	1.736	2.001	1.652	1.837	1.580	65	7.4	4.01
H3BRM5	Cytochrome c oxidase subunit 5A, mitochondrial OS=Homo	4.82	18.84	8	2	2	2	1.401	1.037	1.440	1.453	1.400	1.578	1.446	69	7.8	6.02
P30050	60S ribosomal protein L12 OS=Homo sapiens GN=RPL12 PE	4.80	9.09	1	1	1	1	1.359	0.992	1.441	1.452	1.305	1.583	1.306	165	17.8	9.42
Q6PIX2	SFPQ protein (Fragment) OS=Homo sapiens GN=SFPQ PE=	4.78	5.33	5	3	3	3	1.793	1.233	1.627	1.944	1.538	1.988	1.576	525	55.4	9.89
P18859	ATP synthase-coupling factor 6, mitochondrial OS=Homo sa	4.75	17.59	3	1	1	1	1.608	1.255	1.493	1.503	1.628	1.582	1.547	108	12.6	9.52
B3KUF6	cDNA FLJ39748 fis, clone SMINT2017436, highly similar to I	4.75	7.97	2	1	1	2	1.167	1.066	1.147	1.194	1.090	1.193	1.257	251	28.0	6.71
G3V2D2	Serine hydroxymethyltransferase, mitochondrial OS=Homo s	4.72	26.92	21	1	1	2	1.462	1.068	1.472	1.521	1.316	1.576	1.302	52	5.8	6.74
Q7Z434	Mitochondrial antiviral-signaling protein OS=Homo sapiens G	4.71	5.19	1	1	1	2	0.990	0.960	1.117	1.378	1.096	1.449	0.964	540	56.5	5.52
B5MD38	3-ketoacyl-CoA thiolase OS=Homo sapiens GN=HADHB PE=	4.67	7.41	10	3	3	5	1.533	1.040	1.501	1.693	1.584	1.913	1.463	351	37.9	9.45
B4DZZ0	cDNA FLJ52128, highly similar to PRA1 family protein 3 OS=	4.60	12.12	3	1	1	1	1.156	0.715	1.240	1.087	1.242	0.964	0.948	165	19.2	9.77
Q9UHS8	PRO1975 OS=Homo sapiens PE=2 SV=1 - [Q9UHS8_HUMA	4.59	2.54	4	1	1	2	1.435	1.110	1.455	1.680	1.517	1.390	1.399	393	44.1	9.03
B2R7Y0	cDNA, FLJ93654, highly similar to Homo sapiens serpin pept	4.58	4.58	4	2	2	8	1.871	1.062	1.811	2.229	1.701	1.883	1.614	415	46.6	5.64
D6RBE9	Annexin OS=Homo sapiens GN=ANXA5 PE=2 SV=1 - [D6RE	4.43	3.64	5	1	1	2	2.354	1.173	2.232	2.008	2.204	1.946	1.753	220	24.7	4.89
E5RJD2	2,4-dienoyl-CoA reductase, mitochondrial (Fragment) OS=H	4.38	8.61	6	1	1	2	1.341	1.505	1.354	1.264	1.320	1.483	1.371	151	15.9	8.85
C9J9K3	40S ribosomal protein SA (Fragment) OS=Homo sapiens GN	4.20	10.61	4	2	2	2	1.551	0.897	1.609	1.819	1.497	1.580	1.420	264	29.5	5.25
Q6IPH7	RPL14 protein OS=Homo sapiens GN=RPL14 PE=2 SV=1 -	4.20	13.64	7	2	2	2	2.055	1.041	2.113	2.100	1.815	2.402	1.911	220	23.8	10.93
Q9NS69	Mitochondrial import receptor subunit TOM22 homolog OS=	4.19	7.75	1	1	1	1	1.485	1.143	1.434	1.726	1.546	1.728	1.417	142	15.5	4.34
P30049	ATP synthase subunit delta, mitochondrial OS=Homo sapien	4.15	8.33	1	1	1	1	1.612	1.144	1.452	1.654	1.230	1.418	1.340	168	17.5	5.49
	>sp|ALBU_BOVIN|	4.12	3.62	1	2	2	2	0.983	1.139	0.951	1.123	1.017	1.092	0.997	607	69.2	6.18
P30084	Enoyl-CoA hydratase, mitochondrial OS=Homo sapiens GN=	4.12	4.48	1	1	1	1	1.239	0.908	1.127	1.286	1.106	1.312	0.991	290	31.4	8.07
P62304	Small nuclear ribonucleoprotein E OS=Homo sapiens GN=SN	4.08	25.00	2	2	2	3	1.420	1.466	1.355	1.493	1.396	1.815	1.298	92	10.8	9.44
H7C125	Ras-related protein Rab-2A (Fragment) OS=Homo sapiens G	4.08	13.46	2	1	1	1	1.531	1.041	1.477	1.740	1.477	1.776	2.078	104	12.1	5.08
P07737	Profilin-1 OS=Homo sapiens GN=PFN1 PE=1 SV=2 - [PROF	4.05	11.43	2	1	1	1	2.040	1.134	2.264	2.886	1.915	2.270	2.009	140	15.0	8.27
C9J4S4	Ras-related protein Rab-7a OS=Homo sapiens GN=RAB7A F	3.92	14.29	4	1	1	1	1.500	1.017	1.403	1.661	1.319	1.695	1.491	98	11.0	8.78
E9PLD0	Ras-related protein Rab-1B OS=Homo sapiens GN=RAB1B F	3.91	10.06	4	1	1	1	1.697	1.081	1.773	1.913	1.762	1.999	1.578	169	18.5	5.72
O60250	Ribosomal protein L13 (Fragment) OS=Homo sapiens PE=2	3.88	20.00	3	1	1	1	1.758	0.855	1.758	1.841	1.794	2.163	1.359	65	7.4	10.27
H3BRG4	Cytochrome b-c1 complex subunit 2, mitochondrial OS=Hon	3.82	3.88	2	1	1	1	1.917	1.272	1.721	1.956	1.845	2.122	1.858	412	44.6	9.00
F5H308	L-lactate dehydrogenase OS=Homo sapiens GN=LDHA PE=.	3.60	3.29	3	1	1	1	3.987	1.337	3.126	4.212	2.660	3.351	2.566	304	33.6	8.79
Q6E433	Activated RNA polymerase II transcription cofactor 4 (Fragm	3.58	15.07	3	1	1	1	1.726	1.119	1.889	1.842	1.562	1.767	1.570	73	8.6	8.44
Q53R94	Putative uncharacterized protein RTN4 (Fragment) OS=Hom	3.54	7.03	4	1	1	1	1.612	1.164	1.488	1.329	1.367	1.576	1.401	185	19.3	4.13
Q6DC98	LMNB1 protein (Fragment) OS=Homo sapiens GN=LMNB1 F	3.48	5.71	4	2	2	2	1.485	1.116	1.431	1.730	1.447	1.709	1.311	333	38.1	5.45
O14684	Prostaglandin E synthase OS=Homo sapiens GN=PTGES PE=	3.36	6.58	1	1	1	1	0.898	0.804	0.816	1.019	0.754	0.908	0.604	152	17.1	9.50
C9K0U8	Single-stranded DNA-binding protein (Fragment) OS=Homo	3.35	24.79	5	3	3	3	1.649	1.282	1.669	1.879	1.708	1.807	1.468	121	14.1	9.57
Q9Y2R5	28S ribosomal protein S17, mitochondrial OS=Homo sapiens	3.33	8.46	3	1	1	3	1.563	1.255	1.396	1.249	1.328	1.684	1.225	130	14.5	9.85
Q9NX76	CKLF-like MARVEL transmembrane domain-containing prote	3.28	5.46	1	1	1	2	1.037	1.028	1.054	1.170	1.329	1.529	1.663	183	20.4	5.29
D3WYY8	Cytochrome c oxidase subunit 2 (Fragment) OS=Homo sapi	3.27	26.32	164	1	1	1								38	4.3	4.44
Q6PKI6	YBX1 protein (Fragment) OS=Homo sapiens GN=YBX1 PE=	3.23	7.14	5	1	1	1	1.662	1.009	1.798	1.965	1.711	1.936	1.404	266	29.4	10.23
P47914	60S ribosomal protein L29 OS=Homo sapiens GN=RPL29 PE	3.22	14.47	3	2	2	2	1.486	1.316	1.462	1.716	1.366	1.806	1.405	159	17.7	11.66
B4E1S3	cDNA FLJ57860, highly similar to Transmembrane protein 1(	3.20	5.13	3	1	1	1	1.251	1.004	1.313	1.412	1.319	1.435	1.298	234	25.0	9.54
Q59H46	Integrin beta (Fragment) OS=Homo sapiens PE=2 SV=1 - [(	3.18	1.32	6	2	2	2	1.602	1.132	1.541	1.770	1.616	1.853	1.557	1515	169.0	6.49
A4UCT0	Fructose-bisphosphate aldolase (Fragment) OS=Homo sapie	3.18	16.39	9	1	1	1	1.576	1.116	1.350	1.331	1.386	1.308	1.294	122	13.4	6.77
F8VYN5	Heterogeneous nuclear ribonucleoprotein A1 (Fragment) OS	3.14	8.85	14	1	1	1	1.305	1.147	1.498	1.525	1.558	1.591	1.455	113	12.9	7.64
Q9BVT0	ARHA protein (Fragment) OS=Homo sapiens GN=ARHA PE=	3.12	7.92	6	1	1	1	1.842	1.106	1.842	2.436	1.782	2.161	1.796	101	11.5	9.35
P49419	Alpha-aminoadipic semialdehyde dehydrogenase OS=Homo	3.11	3.15	1	1	1	3	1.651	1.096	1.622	1.869	1.734	1.758	1.550	539	58.5	7.99
P51572	B-cell receptor-associated protein 31 OS=Homo sapiens GN	3.08	3.66	3	1	1	1	1.411	0.918	1.507	1.829	1.612	1.684	1.555	246	28.0	8.44
J3QSA3	Ubiquitin (Fragment) OS=Homo sapiens GN=UBB PE=4 SV=	3.07	37.21	41	1	1	1	1.390	1.046	1.672	1.870	1.412	1.560	1.435	43	4.9	5.19
B4DSH1	cDNA FLJ51295, highly similar to Cell division cycle 5-like pr	3.05	2.45	3	1	1	1	1.304	0.940	1.157	1.197	1.148	1.428	1.395	775	89.2	8.54
F5H823	Ras-related protein Rap-1b (Fragment) OS=Homo sapiens G	3.04	11.65	8	1	1	1	1.841	1.354	1.687	2.085	1.740	2.082	1.639	103	11.9	5.55
B4DE36	Glucose-6-phosphate isomerase OS=Homo sapiens PE=2 S\	3.02	4.91	3	2	2	4	1.579	1.047	1.202	1.254	1.436	1.615	1.302	530	60.1	8.15
Q14BN3	PKP1 protein OS=Homo sapiens GN=PKP1 PE=2 SV=1 - [Q	3.00	4.19	2	1	1	1	1.323	0.940	1.581	1.231	1.289	1.552	1.330	334	36.8	8.56
M0R221	U1 small nuclear ribonucleoprotein A (Fragment) OS=Homo	2.98	9.15	5	1	1	1	1.886	1.176	1.704	2.027	1.666	1.953	1.621	142	15.9	10.11
E9PP36	60S ribosomal protein L8 OS=Homo sapiens GN=RPL8 PE=.	2.96	7.43	5	1	1	1	1.687	1.124	1.491	1.664	1.535	1.692	1.376	148	16.2	11.90
E7EX53	Ribosomal protein L15 (Fragment) OS=Homo sapiens GN=F	2.88	6.77	4	1	1	1	2.214	1.276	1.875	2.090	1.913	2.291	1.959	133	15.7	11.00
J3QRP6	Na(+)/H(+) exchange regulatory cofactor NHE-RF1 (Fragme	2.86	12.09	4	1	1	1	1.528	1.424	1.224	1.547	1.288	2.012	1.021	215	22.9	4.82
B4DSW9	cDNA FLJ59415, highly similar to Beta-catenin OS=Homo sa	2.82	4.23	5	3	3	3	1.910	1.216	1.993	2.323	1.761	2.112	1.885	709	77.5	6.38
H0YGF3	Pre-mRNA-processing factor 19 (Fragment) OS=Homo sapie	2.76	17.78	4	1	1	1	1.452	1.061	1.598	1.660	1.619	1.933	1.655	45	5.2	9.55
Q99714	3-hydroxyacyl-CoA dehydrogenase type-2 OS=Homo sapien	2.75	4.60	1	1	1	1	0.942	0.998	0.634	0.788	0.533	0.592	0.537	261	26.9	7.78
B3KP09	cDNA FLJ30894 fis, clone FEBRA2005416, highly similar to I	2.71	3.77	4	2	2	2	2.008	1.069	1.919	1.828	1.571	2.063	1.557	531	59.3	6.55
E9PPU1	40S ribosomal protein S3 OS=Homo sapiens GN=RPS3 PE=	2.70	10.13	7	1	1	1	2.097	1.389	1.919	2.455	1.795	2.230	1.896	158	17.4	9.50
M0QY67	Electron transfer flavoprotein subunit beta (Fragment) OS=I	2.67	6.74	3	1	1	1	1.700	1.133	1.550	1.872	1.587	1.804	1.576	178	19.5	7.74
G3V4Y7	Kinectin OS=Homo sapiens GN=KTN1 PE=2 SV=1 - [G3V4Y	2.65	2.02	4	1	1	1	1.941	0.977	1.964	2.264	1.925	2.224	1.763	595	68.9	5.22
Q9HCY8	Protein S100-A14 OS=Homo sapiens GN=S100A14 PE=1 S\	2.65	25.00	1	2	2	3	1.175	1.100	1.146	1.484	1.141	1.245	1.120	104	11.7	5.24

Q8TCT9	Minor histocompatibility antigen H13 OS=Homo sapiens GN=	2.65	3.18	1	1	1	1	1.153	1.088	1.274	1.253	1.191	1.288	1.364	377	41.5	6.43
C9JK10	Integrin alpha-6 heavy chain (Fragment) OS=Homo sapiens	2.64	0.74	4	1	1	1	1.321	1.104	1.435	1.574	1.459	1.713	1.382	1086	121.6	7.36
P0CG22	Putative dehydrogenase/reductase SDR family member 4-lik	2.57	6.41	1	1	1	1	1.600	1.125	1.450	1.544	1.483	1.781	1.501	281	30.6	9.77
O15400	Syntaxin-7 OS=Homo sapiens GN=STX7 PE=1 SV=4 - [STX	2.57	5.36	1	1	1	1	1.943	1.348	1.766	2.025	1.660	1.862	1.385	261	29.8	5.55
Q05DN3	IMMT protein (Fragment) OS=Homo sapiens GN=IMMT PE=	2.53	8.86	9	1	1	3	1.773	1.283	2.216	1.991	1.672	1.988	1.576	316	33.7	9.31
E9PKV2	39S ribosomal protein L17, mitochondrial (Fragment) OS=H	2.53	5.63	2	1	1	1	1.579	1.206	1.304	1.578	1.638	1.600	1.274	142	16.4	10.48
B4E364	cDNA FLJ54954, highly similar to Ras-related protein Ral-B (	2.53	6.93	5	1	1	1	1.793	1.131	1.697	1.913	1.604	1.942	1.656	101	11.8	9.51
M0QYG1	Far upstream element-binding protein 2 (Fragment) OS=Ho	2.52	15.79	3	1	1	1	1.669	1.096	1.883	1.940	1.813	2.008	1.726	114	12.4	7.84
B4E0X6	Proteasome subunit alpha type OS=Homo sapiens PE=2 SV	2.52	8.46	4	1	1	1	1.504	0.982	1.383	1.754	1.437	1.566	1.424	130	14.6	8.51
Q59GT8	BM-010 variant (Fragment) OS=Homo sapiens PE=2 SV=1 -	2.51	23.88	31	1	1	1	1.819	1.027	1.912	2.400	1.678	2.051	1.390	67	7.7	5.35
H0YCY8	Dipeptidyl peptidase 1 exclusion domain chain (Fragment) C	2.51	4.90	3	1	1	1	1.582	1.145	1.500	1.632	1.529	1.765	1.533	245	27.9	9.16
B4E2M8	cDNA FLJ61076, highly similar to UBX domain-containing pr	2.49	5.66	2	1	1	1	1.499	1.114	1.238	1.409	1.341	1.556	1.298	212	24.7	5.74
P62263	40S ribosomal protein S14 OS=Homo sapiens GN=RPS14 PI	2.49	8.61	1	1	1	1	1.575	1.177	1.589	1.874	1.575	1.843	1.430	151	16.3	10.05
Q49AJ9	RPL3 protein OS=Homo sapiens GN=RPL3 PE=2 SV=1 - [Q	2.47	2.79	10	1	1	1	1.419	1.009	1.381	1.656	1.511	1.598	1.486	251	28.6	10.17
A1A5C5	RRBP1 protein OS=Homo sapiens GN=RRBP1 PE=2 SV=1 -	2.43	1.73	5	1	1	1	1.786	1.253	1.727	1.739	1.814	2.188	1.770	751	84.3	5.01
A8K5W7	cDNA FLJ75180, highly similar to Homo sapiens mitochondri	2.43	1.28	3	1	1	2	1.550	1.148	1.775	1.986	1.671	1.688	1.312	940	105.9	6.42
Q96B49	Mitochondrial import receptor subunit TOM6 homolog OS=H	2.42	18.92	1	1	1	1	1.207	1.072	1.219	1.574	1.342	1.590	1.353	74	8.0	4.89
B7Z9S8	Sodium/potassium-transporting ATPase subunit beta-1 OS=	2.42	5.67	4	1	1	1	1.262	1.009	1.208	1.277	1.085	1.194	1.123	247	28.6	7.59
Q5JP01	Histone-binding protein RBBP7 (Fragment) OS=Homo sapie	2.40	4.21	4	1	1	1	1.770	1.098	1.731	2.151	1.689	2.014	1.672	285	31.5	6.19
B4E0S6	cDNA FLJ55635, highly similar to pre-mRNA-splicing factorA	2.39	1.28	2	1	1	1	1.575	1.154	1.355	1.507	1.434	1.661	1.612	784	89.5	7.46
	>sp|TRYP_PIG|	2.37	14.72	7	1	2	16	1.447	1.103	1.444	1.529	1.446	1.829	1.177	231	24.4	7.18
B4E2K4	cDNA FLJ54576, highly similar to Aspartyl/asparaginyl beta-	2.35	3.16	13	3	3	3	2.169	1.233	1.809	1.945	2.101	1.942	1.798	729	83.2	4.94
P05109	Protein S100-A8 OS=Homo sapiens GN=S100A8 PE=1 SV=	2.31	8.60	1	1	1	1	1.072	0.946	1.031	1.238	1.022	1.213	1.021	93	10.8	7.03
J3KRY3	Small nuclear ribonucleoprotein-associated protein N (Fragm	2.31	11.86	12	1	1	1	1.881	1.101	1.774	2.200	1.858	1.954	1.726	59	7.0	9.52
B4DXF1	cDNA FLJ57223 OS=Homo sapiens PE=2 SV=1 - [B4DXF1_	2.31	8.97	4	1	1	2	1.458	1.029	1.373	1.591	1.327	1.522	1.205	156	16.8	7.72
H7C4L9	Sodium/potassium-transporting ATPase subunit beta-3 (Frag	2.30	40.74	6	1	1	1	1.510	1.047	1.423	1.506	1.392	1.602	1.223	27	2.8	8.22
B4E0E1	cDNA FLJ53442, highly similar to Poly (ADP-ribose) polymer	2.30	1.31	3	1	1	1	1.842	1.114	1.668	1.843	1.710	1.861	1.566	993	111.1	8.87
B2R4C1	cDNA, FLJ92036, highly similar to Homo sapiens ribosomal	2.29	15.20	8	2	2	5	1.303	1.160	1.172	1.362	1.265	1.385	1.510	125	14.5	10.54
P51148	Ras-related protein Rab-5C OS=Homo sapiens GN=RAB5C I	2.29	6.48	1	1	1	1	2.086	1.390	1.822	2.028	1.977	1.985	1.828	216	23.5	8.41
J3QL15	Ribosomal protein L19 (Fragment) OS=Homo sapiens GN=R	2.29	5.47	5	1	1	1	1.849	1.173	1.756	1.955	1.823	2.123	1.711	128	15.0	11.97
Q86TW7	Dihydrolipoamide S-succinyltransferase (E2 component of 2-	2.28	3.19	5	1	1	1	1.591	1.145	1.657	1.950	1.732	1.871	1.792	251	26.9	10.08
H0YCG2	Lysosome-associated membrane glycoprotein 2 (Fragment)	2.26	2.71	5	1	1	1	1.432	1.047	1.278	1.446	1.464	1.630	1.393	258	28.2	6.52
B4DTM7	Uncharacterized protein OS=Homo sapiens GN=VCL PE=2 S	2.24	6.42	2	1	1	2								327	36.2	6.44
I3L192	Basigin (Fragment) OS=Homo sapiens GN=BSG PE=2 SV=1	2.23	5.59	6	1	1	1	1.656	1.206	1.729	2.015	1.730	2.011	1.532	161	17.3	5.05
Q2Z195	MHC class I antigen (Fragment) OS=Homo sapiens GN=HL/	2.23	12.36	228	1	1	3	1.393	0.944	1.340	1.451	1.313	1.601	1.288	89	10.3	6.05
P07195	L-lactate dehydrogenase B chain OS=Homo sapiens GN=LD	2.12	5.39	2	2	2	2	2.274	1.141	2.249	2.544	1.953	2.344	2.058	334	36.6	6.05
Q96RX5	NADH ubiquinone oxidoreductase PDSW subunit (RH 16p13	2.12	32.56	5	1	1	1	1.134	1.328	1.603	1.648	1.397	1.465	1.230	43	5.1	6.60
Q8N183	Mimitin, mitochondrial OS=Homo sapiens GN=NDUFAF2 PE	2.08	5.92	1	1	1	1	1.814	1.113	1.795	2.028	1.761	2.316	1.727	169	19.8	8.97
Q7Z4Q5	Heterogeneous nuclear ribonucleoprotein U (Scaffold attachi	2.06	1.36	5	1	1	1	1.288	1.039	1.262	1.363	1.267	1.420	1.313	513	57.6	9.36
B7ZW15	Putative uncharacterized protein OS=Homo sapiens PE=2 S\	2.06	11.20	12	1	1	1	3.389	1.104	3.587	5.411	2.806	3.394	2.650	125	13.9	5.31
D6R9P3	Heterogeneous nuclear ribonucleoprotein A/B OS=Homo sa	2.04	7.50	6	2	2	4	1.939	1.163	1.863	2.104	1.905	1.954	1.642	280	30.3	7.91
F8WCJ1	Eukaryotic translation initiation factor 5A-2 OS=Homo sapie	2.02	11.43	8	1	1	3	1.747	1.034	1.808	2.187	1.723	1.959	1.500	105	11.7	9.14
B7Z800	Adenosine kinase OS=Homo sapiens GN=ADK PE=2 SV=1 -	2.00	3.36	4	1	1	2	1.626	0.831	1.604	2.221	1.481	1.630	1.438	327	36.6	6.79
Q4ZG51	Putative uncharacterized protein FNBP3 OS=Homo sapiens (	2.00	4.88	2	1	1	1	1.602	1.141	1.639	1.954	1.739	1.950	1.768	205	24.3	9.72
H0Y7X6	Spliceosome RNA helicase DDX39B (Fragment) OS=Homo s	1.95	8.62	33	3	3	6	1.838	1.184	1.858	2.181	1.711	1.861	1.560	325	38.0	8.95
D6R9X8	Integrin alpha-3 OS=Homo sapiens GN=ITGA3 PE=2 SV=2	1.91	6.34	4	1	1	1	1.705	1.076	1.597	1.722	1.660	1.821	1.642	142	15.2	8.21
P62269	40S ribosomal protein S18 OS=Homo sapiens GN=RPS18 PI	1.91	9.87	2	2	2	2	1.738	1.076	1.803	1.924	2.007	1.976	1.482	152	17.7	10.99
Q8IXM3	39S ribosomal protein L41, mitochondrial OS=Homo sapiens	1.72	5.11	1	1	1	1	1.387	2.322	1.389	1.387	1.017	1.219	0.998	137	15.4	9.57
Q15388	Mitochondrial import receptor subunit TOM20 homolog OS=	1.72	8.97	1	1	1	2	1.346	0.696	0.837	1.023	0.934	1.123	1.027	145	16.3	8.60
B2R5H0	cDNA, FLJ92471, highly similar to Homo sapiens S100 calciu	1.70	8.57	2	1	1	1	1.704	12.377	1.680	1.814	1.641	1.902	1.522	105	11.7	7.18
I3L3P7	40S ribosomal protein S15a OS=Homo sapiens GN=RPS15A	1.68	8.00	4	1	1	1	1.752	1.222	1.872	1.998	1.855	2.073	1.671	100	11.5	10.15
Q9Y2S7	Polymerase delta-interacting protein 2 OS=Homo sapiens Gl	1.68	2.72	1	1	1	1	1.564	1.113	1.421	1.524	1.414	1.464	1.322	368	42.0	8.63
Q6NVV1	Putative 60S ribosomal protein L13a-like MGC87657 OS=Ho	1.64	7.84	8	1	1	1	1.798	1.240	1.649	1.831	1.703	1.740	1.663	102	12.1	10.76
B4E1K7	Stomatin-like protein 2 OS=Homo sapiens GN=STOML2 PE=	1.64	9.97	2	2	2	4	0.982	1.194	0.812	0.857	0.962	0.906	0.809	311	33.3	8.25
Q8TAS0	ATP synthase subunit gamma (Fragment) OS=Homo sapien	1.62	4.47	2	1	1	1	1.320	1.368	1.182	1.612	1.434	1.965	1.347	291	32.2	9.11
Q86UK0	ATP-binding cassette sub-family A member 12 OS=Homo sa	0.00	0.31	1	1	1	1								2595	293.0	7.75
P55291	Cadherin-15 OS=Homo sapiens GN=CDH15 PE=1 SV=1 - [(	0.00	1.84	1	1	1	1	1.267	0.929	1.264	1.422	1.331	1.559	1.337	814	88.9	4.98
Q92905	COP9 signalosome complex subunit 5 OS=Homo sapiens GN	0.00	2.40	1	1	1	2								334	37.6	6.54
Q9NR30	Nucleolar RNA helicase 2 OS=Homo sapiens GN=DDX21 PE	0.00	1.66	1	1	1	1	1.654	1.071	1.583	1.828	1.563	1.721	1.522	783	87.3	9.28
Q15717	ELAV-like protein 1 OS=Homo sapiens GN=ELAVL1 PE=1 S\	0.00	4.29	2	1	1	1	1.384	2.054	1.360	1.425	1.512	1.574	1.494	326	36.1	9.17
Q96I24	Far upstream element-binding protein 3 OS=Homo sapiens	0.00	5.59	1	1	1	2	1.233	0.886	1.374	1.383	1.037	1.176	1.047	572	61.6	8.38
P01617	Ig kappa chain V-II region TEW OS=Homo sapiens PE=1 S\	0.00	23.01	1	1	1	1								113	12.3	6.00
O75197	Low-density lipoprotein receptor-related protein 5 OS=Hom	0.00	1.73	1	1	1	1								1615	179.0	5.34
Q14324	Myosin-binding protein C, fast-type OS=Homo sapiens GN=	0.00	3.86	1	1	1	1								1141	128.0	7.52
Q8WWR8	Sialidase-4 OS=Homo sapiens GN=NEU4 PE=1 SV=3 - [NEI	0.00	5.58	5	1	1	2	1.574	1.075	1.504	1.527	1.487	1.747	1.322	484	51.5	7.80
P12004	Proliferating cell nuclear antigen OS=Homo sapiens GN=PCI	0.00	2.68	3	1	1	1	2.303	0.797	1.979	1.891	1.679	2.172	1.731	261	28.8	4.69
P62891	60S ribosomal protein L39 OS=Homo sapiens GN=RPL39 PE	0.00	19.61	2	1	1	1	2.001	1.284	1.931	1.845	1.850	2.083	1.669	51	6.4	12.56

E7ERA6	RING finger protein 223 OS=Homo sapiens GN=RNF223 PE	0.00	7.63	1	1	1	2	1.222	0.887	1.119	1.079	1.021	0.940	0.860	249	26.6	9.04
P62854	40S ribosomal protein S26 OS=Homo sapiens GN=RPS26 PI	0.00	13.04	2	1	1	1	2.156	1.185	2.201	2.443	2.378	2.537	1.545	115	13.0	11.00
P82673	28S ribosomal protein S35, mitochondrial OS=Homo sapiens	0.00	3.10	1	1	1	1	1.537	1.475	1.496	1.589	1.435	1.507	1.427	323	36.8	8.24
P35712	Transcription factor SOX-6 OS=Homo sapiens GN=SOX6 PE	0.00	3.50	1	1	1	1								828	91.9	7.78
Q13813	Spectrin alpha chain, non-erythrocytic 1 OS=Homo sapiens	0.00	1.09	6	2	2	2	1.011	0.700	1.089	1.214	1.066	1.287	1.201	2472	284.4	5.35
Q92750	Transcription initiation factor TFIID subunit 4B OS=Homo sa	0.00	3.83	3	1	1	1	1.518	1.337	1.506	1.356	1.307	1.393	1.526	862	91.0	9.54
Q9P273	Teneurin-3 OS=Homo sapiens GN=TENM3 PE=2 SV=3 - [TI	0.00	1.48	1	1	1	1								2699	300.8	6.42
P25942	Tumor necrosis factor receptor superfamily member 5 OS=H	0.00	8.30	1	1	1	1								277	30.6	5.76
O75691	Small subunit processome component 20 homolog OS=Hom	0.00	1.01	1	1	1	1		0.869	1.325	0.954		1.380		2785	318.2	7.39
A8MPV3	Putative uncharacterized protein ENSP00000344348 OS=Ho	0.00	2.28	1	1	1	1	0.835	0.773	1.094	0.802	1.018	1.022	1.150	395	46.2	6.02
B3KRB7	Inhibitor of kappa light polypeptide gene enhancer in B-cells	0.00	1.69	3	1	1	1	1.516	1.047	1.198	1.407	1.359	1.538	1.377	533	60.8	5.58
K7ERI7	60S ribosomal protein L22 OS=Homo sapiens GN=RPL22 PE	0.00	15.79	3	1	1	2	1.505	1.162	1.375	1.660	1.566	1.959	2.119	95	11.2	6.80
B4DSZ6	Isocitrate dehydrogenase [NADP], mitochondrial OS=Homo	0.00	4.04	5	1	1	1	1.622	1.364	1.752	1.739	1.962	2.002	1.695	322	36.1	8.38
D3DPF9	Titin, isoform CRA_b OS=Homo sapiens GN=TTN PE=2 SV=	0.00	0.09	4	1	1	3	1.977	1.146	1.910	2.590	2.034	2.447	1.741	26926	2991.2	6.74
D3DWL9	Cleavage and polyadenylation specific factor 1, 160kDa, isof	0.00	1.47	2	1	1	1	1.861	1.047	1.899	2.404	1.607	1.892	1.483	1365	151.9	6.33
B4E072	3-ketoacyl-CoA thiolase, peroxisomal OS=Homo sapiens GN	0.00	4.76	7	1	1	1	1.611	0.970	1.613	1.873	1.397	1.592	1.326	126	13.5	8.85
H3BR27	RNA-binding motif protein, X chromosome, N-terminally pro	0.00	10.26	6	1	1	1		1.162		0.678		0.853	1.183	78	8.6	5.49
B4DKM5	Voltage-dependent anion-selective channel protein 2 OS=Ho	0.00	3.92	2	1	1	1	1.289	0.991	1.294	1.290	1.228	1.320	1.157	255	27.5	5.47
Q9UMZ1	Prothymosin a14 OS=Homo sapiens PE=1 SV=1 - [Q9UMZ1	0.00	12.87	1	1	1	1	1.440	0.981	1.576	1.776	1.463	1.758	1.435	101	11.1	3.79
Q13841	Beta-centractin (Fragment) OS=Homo sapiens PE=2 SV=1 -	0.00	2.74	2	1	1	1	1.707	1.230	1.576	1.923	1.601	1.798	1.583	329	37.1	6.62
Q53TL0	Putative uncharacterized protein LRP2 (Fragment) OS=Hom	0.00	5.05	5	1	1	2								773	86.1	4.87
Q2MD46	B-cell linker protein (Fragment) OS=Homo sapiens GN=BAS	0.00	10.05	7	1	1	2								209	23.7	5.29
B2R4M6	cDNA, FLJ92148, highly similar to Homo sapiens S100 calciu	0.00	13.16	2	1	1	2	1.013	1.002	1.276	1.578	1.135	1.411	1.227	114	13.2	6.13
B4DYC0	cDNA FLJ57012, highly similar to AT-rich interactive domain	0.00	3.17	4	1	1	1								757	84.3	5.25
B4E0B9	cDNA FLJ54526, highly similar to 5'-3' exoribonuclease 2 (E(	0.00	1.14	4	1	1	1	1.652	1.139	1.762	2.119	1.900	1.896	1.532	874	99.9	8.02
B4DHA5	cDNA FLJ61253, highly similar to Low-density lipoprotein re	0.00	2.86	1	1	1	3								1225	136.1	4.97
B4E1K4	cDNA FLJ51563 OS=Homo sapiens PE=2 SV=1 - [B4E1K4_I	0.00	13.61	2	1	1	1	0.899	0.888	1.252	1.021	0.989	1.732	0.534	191	21.4	7.49
B7Z636	cDNA FLJ55887, highly similar to Ankyrin-2 (Fragment) OS=	0.00	0.81	4	1	1	1								1726	188.5	7.20
B4DLF0	cDNA FLJ50795, highly similar to Cadherin-3 OS=Homo sap	0.00	1.42	7	1	1	1	1.154	0.940	1.006	1.409	1.199	1.223	1.086	774	85.6	4.77
H3BRA8	Nucleoside diphosphate kinase 3 (Fragment) OS=Homo sap	0.00	6.50	1	1	1	1	1.364	1.007	1.064	1.677	1.368	1.541	0.942	123	13.5	11.71
E9PK86	Serpin H1 (Fragment) OS=Homo sapiens GN=SERPINH1 PE	0.00	14.98	14	2	2	2	1.153	1.269	0.989	1.449	1.177	1.282	1.072	247	26.9	8.90
J3KPX7	Prohibitin-2 OS=Homo sapiens GN=PHB2 PE=4 SV=1 - [J3K	0.00	6.69	4	2	2	3	1.174	0.874	1.347	1.435	1.238	1.389	0.979	299	33.4	9.80
B4DYX7	cDNA FLJ58647, highly similar to Signal-induced proliferatio	0.00	0.88	5	1	1	1	1.409	0.996	1.459	1.603	1.534	1.688	1.963	1257	139.4	8.18
J3QLI9	Small nuclear ribonucleoprotein Sm D1 OS=Homo sapiens G	0.00	26.67	3	1	1	1								75	8.4	11.84
M0R3C3	Very-long-chain enoyl-CoA reductase OS=Homo sapiens GN	0.00	8.57	5	1	1	1	1.572	1.116	1.540	1.658	1.499	1.848	1.497	105	11.8	9.20
K7EKT0	Dedicator of cytokinesis protein 6 (Fragment) OS=Homo sa	0.00	6.49	1	1	1	1								185	19.4	8.66
Q9H5C8	cDNA: FLJ23571 fis, clone LNG12303 OS=Homo sapiens PE	0.00	2.71	1	1	1	1	2.137	1.095	1.949	2.392	2.096	2.037	1.688	443	51.8	6.51
H0YH88	Nucleosome assembly protein 1-like 1 (Fragment) OS=Hom	0.00	6.21	17	1	1	2	1.092	0.808	0.816	0.711	0.860	0.992	0.780	177	21.1	4.83
B2RAQ9	Proteasome subunit beta type OS=Homo sapiens PE=2 SV=	0.00	6.86	4	1	1	1								277	29.9	7.68
F5H5Y2	Translocon-associated protein subunit alpha OS=Homo sapi	0.00	3.09	7	1	1	1	1.614	1.427	1.514	1.677	1.395	1.584	1.334	259	29.4	4.72
E5RHJ4	DBIRD complex subunit KIAA1967 (Fragment) OS=Homo sa	0.00	7.94	3	1	1	1	2.144	1.670	1.903	1.800	1.669	2.128	1.417	277	31.1	5.29
B4DUP0	cDNA FLJ59433, highly similar to Elongation factor 1-gamma	0.00	4.85	4	1	1	1	2.181	1.202	2.186	3.112	2.205	2.518	1.962	206	24.1	7.05
H0YB86	Polyadenylate-binding protein 1 (Fragment) OS=Homo sapie	0.00	8.48	12	1	1	2	1.935	1.213	1.855	2.087	1.943	2.003	1.600	165	18.1	9.82
F8VY02	Endoplasmic reticulum resident protein 29 OS=Homo sapier	0.00	6.25	2	1	1	1	1.948	1.254	1.608	1.780	1.696	1.968	1.329	160	18.1	8.00
H0Y7C8	Torsin-1B (Fragment) OS=Homo sapiens GN=TOR1B PE=4	0.00	9.93	2	1	1	1	1.354	1.010	1.626	1.523	1.376	1.602	1.446	141	15.7	9.10
B4DHX4	cDNA FLJ52902, highly similar to Rab GDP dissociation inhib	0.00	3.60	2	1	1	1	1.653	1.042	1.456	1.958	1.404	2.269	1.478	417	46.9	5.05
I1VE16	SEC22 vesicle trafficking protein B (Fragment) OS=Homo sa	0.00	27.78	2	1	1	1	1.619	1.227	1.533	1.575	1.613	1.755	1.438	36	4.1	9.04
Q9P1K6	PRO1094 OS=Homo sapiens PE=4 SV=1 - [Q9P1K6_HUMAI	0.00	19.05	1	1	1	2	1.771	1.382	1.523	1.757	1.518	1.465	1.153	84	9.9	9.73
B4DF97	cDNA FLJ59673, highly similar to Homo sapiens growth and	0.00	4.96	4	1	1	1	1.573	1.158	1.544	2.392	1.640	1.592	1.310	141	15.9	9.95
B2RAU8	cDNA, FLJ95131, highly similar to Homo sapiens nucleolar a	0.00	1.14	2	1	1	1	1.947	1.102	2.159	2.454	1.901	2.193	1.834	699	73.6	9.42
H0Y5A6	EF-hand domain-containing family member C2 (Fragment) (	0.00	1.78	4	1	1	1	1.353	1.048	1.368	1.417	1.007	1.349	1.386	730	85.2	6.89
B7Z431	cDNA FLJ51510, highly similar to Exostosin-2 (EC 2.4.1.224)	0.00	3.86	4	1	1	1								337	38.3	7.56
B4DLL8	cDNA FLJ59335, highly similar to Transmembrane glycoprot	0.00	1.98	10	1	1	1	1.866	1.303	1.857	1.950	1.474	1.639	1.010	455	50.5	6.24
Q9HD54	Uncharacterized gastric protein ZA51P (Fragment) OS=Hom	0.00	47.62	1	1	1	1	1.859	1.221	1.515	2.183	1.699	1.941	1.159	21	2.5	12.31
C9J8E0	Adenylate cyclase type 3 (Fragment) OS=Homo sapiens GN	0.00	27.81	5	1	1	1								169	19.1	7.33
F5H1Y3	Flap endonuclease 1 (Fragment) OS=Homo sapiens GN=FEI	0.00	9.70	3	1	1	1	1.043	0.962	1.036	1.057	0.980	1.159	0.945	165	18.3	7.37
B1AP42	Tetratricopeptide repeat protein 40 (Fragment) OS=Homo s	0.00	9.55	1	1	1	1	1.323	0.714	0.907	0.824	1.104	0.886	0.740	220	24.4	5.01
B4DN39	cDNA FLJ53065, highly similar to T-complex protein 1 subur	0.00	4.37	6	1	1	2	1.711	0.806	1.450	2.474	1.554	2.234	1.188	389	42.9	6.62
H0Y512	Adipocyte plasma membrane-associated protein (Fragment)	0.00	2.93	2	1	1	1	1.340	0.927	1.306	1.586	1.271	1.331	1.261	409	45.4	5.66
B4DSE2	cDNA FLJ57277, highly similar to Tripeptidyl-peptidase 1 (E(	0.00	3.64	6	1	1	1	1.483	0.916	1.415	1.695	1.307	1.673	1.226	385	41.6	5.45
F8WE98	Filamin-A (Fragment) OS=Homo sapiens GN=FLNA PE=2 S\	0.00	1.99	6	1	1	2	1.734	1.155	1.338	1.645	1.454	1.639	1.445	604	66.6	8.95
H0YAW4	Eukaryotic translation initiation factor 3 subunit E (Fragment	0.00	8.97	6	1	1	1	1.358	0.852	1.772	1.622	1.795	1.377	1.539	156	18.1	7.58
B4DZD7	cDNA FLJ58963, highly similar to Beta-glucuronidase-like pr	0.00	15.82	1	1	1	1	1.402	0.974	1.436	1.385	1.546	1.487	1.250	158	17.4	7.24
B3KN64	cDNA FLJ13762 fis, clone PLACE4000014, weakly similar to	0.00	1.13	6	1	1	1								793	86.4	8.59
H7BZD1	Glutaminase kidney isoform, mitochondrial (Fragment) OS=	0.00	22.81	5	1	1	1	0.684	0.654	0.573	0.767	0.519	0.698	0.508	114	12.6	8.28
F8WF03	Sphingomyelin phosphodiesterase 4 OS=Homo sapiens GN=	0.00	68.00	5	1	1	1								50	5.6	5.10
Q5JT61	Ras-specific guanine nucleotide-releasing factor RalGPS1 (Fr	0.00	16.18	1	1	1	1								173	19.6	9.10

Q6ZNX4	CDNA FLJ26942 fis, clone RCT07464 OS=Homo sapiens PE=	0.00	4.81	1	1	1	1	0.795	1.045	1.015	1.016	1.469	1.677	0.760	208	22.9	9.94
E9PL01	Signal peptidase complex subunit 2 OS=Homo sapiens GN=	0.00	5.10	5	1	1	1	1.049	0.962	0.743	1.191	0.789	1.161	0.946	157	17.0	8.95
B2RD22	cDNA, FLJ96422, highly similar to Homo sapiens transmemb	0.00	9.36	3	1	1	1								374	41.5	5.15
E7EUY0	DNA-dependent protein kinase catalytic subunit OS=Homo s	0.00	0.66	2	2	2	2	1.406	1.053	1.686	1.721	1.608	1.883	1.470	4096	465.1	7.17
E9PGX9	Lupus La protein (Fragment) OS=Homo sapiens GN=SSB PE	0.00	11.67	6	1	1	2	2.474	1.150	2.405	2.060	1.723	1.993	2.037	120	13.9	9.14
D6RAA0	PR domain zinc finger protein 2 (Fragment) OS=Homo sapie	0.00	13.07	1	1	1	1								153	17.3	5.67
B4DUL5	cDNA FLJ51625, highly similar to Ubiquinol-cytochrome-c re	0.00	1.92	2	1	1	1	1.131	0.874	1.150	1.541	1.227	1.303	1.090	365	40.3	5.88
Q14214	Nebulin (Fragment) OS=Homo sapiens PE=2 SV=1 - [Q142	0.00	0.57	5	1	1	1	2.092	1.214	2.353	2.935	1.890	2.262	1.854	2472	285.6	9.13
H0YHS6	Tyrosine--tRNA ligase, mitochondrial (Fragment) OS=Homo	0.00	4.47	2	1	1	1	4.135	1.434	3.435	4.110	3.030	3.331	2.598	291	32.2	8.40
F8W6N6	Myb-related protein B OS=Homo sapiens GN=MYBL2 PE=2	0.00	3.70	3	1	1	1	1.252	1.222	1.069	1.761	1.200	1.660	1.343	676	75.8	6.84
H3BNL9	Gamma-tubulin complex component 4 (Fragment) OS=Hom	0.00	16.37	2	1	1	1								171	20.1	9.77
E7EMF1	Integrin alpha-2 OS=Homo sapiens GN=ITGA2 PE=2 SV=1	0.00	0.98	3	1	1	1	1.459	0.837	0.915	1.078	1.261	1.121	0.930	815	88.5	5.22
C9JTK6	Obg-like ATPase 1 (Fragment) OS=Homo sapiens GN=OLA1	0.00	12.93	5	1	1	1								116	12.3	8.91
B4DE42	cDNA FLJ60422, highly similar to Myb-binding protein 1A OS	0.00	3.24	3	1	1	1	1.184	0.933	1.145	1.323	1.004	1.039	0.862	370	40.3	10.74
B4DXV7	cDNA FLJ57661, highly similar to GMP synthase (glutamine-	0.00	1.85	5	1	1	1	2.506	0.981	2.342	2.758	1.931	2.397	1.428	594	65.9	7.30
A8K6A5	cDNA FLJ77742, highly similar to Homo sapiens integrin, alp	0.00	0.67	3	1	1	1								1049	114.4	5.71
E7ETC2	Serine/threonine-protein phosphatase OS=Homo sapiens GN	0.00	9.46	3	1	1	1								423	47.8	5.73
H7C1F9	Ral GTPase-activating protein subunit alpha-2 (Fragment) O	0.00	0.57	2	1	1	1	1.720	1.233	1.577	2.019	1.757	1.854	1.553	1740	194.9	5.90
Q8NDS5	Microtubule-associated protein (Fragment) OS=Homo sapier	0.00	7.49	6	1	1	2	1.146	0.796	1.747	1.844	1.162	1.298	0.891	187	19.1	9.29
Q71UF1	Aconitase OS=Homo sapiens GN=ACO2 PE=2 SV=1 - [Q71U	0.00	2.82	3	1	1	1	1.494	0.836	1.681	1.390	1.436	1.074	1.460	780	85.6	7.49
E9PAR0	Peptidyl-prolyl cis-trans isomerase OS=Homo sapiens GN=F	0.00	11.11	3	1	1	1	1.480	1.097	1.392	1.476	1.274	1.306	1.173	99	11.2	10.36
Q96IR1	RPS4X protein (Fragment) OS=Homo sapiens GN=RPS4X PE	0.00	4.12	3	1	1	4	1.727	1.153	1.809	1.964	1.713	1.972	1.623	243	27.2	9.94
Q96IF9	VCP protein (Fragment) OS=Homo sapiens GN=VCP PE=2 S	0.00	3.57	6	2	2	4	1.870	1.231	1.761	1.976	1.744	1.871	1.578	644	71.0	5.06
K7EQU6	ATP synthase subunit alpha, mitochondrial OS=Homo sapier	0.00	35.90	1	1	1	1	1.457	1.423	1.985	1.590	1.608	1.732	1.619	78	9.4	10.70
B2R8D2	cDNA, FLJ93842 OS=Homo sapiens PE=2 SV=1 - [B2R8D2_	0.00	5.71	1	1	1	1								280	32.4	5.20
Q711P9	Putative uncharacterized protein (Fragment) OS=Homo sapi	0.00	3.16	2	1	1	1								443	48.8	5.22
B4DZ36	cDNA FLJ58441, highly similar to Attractin OS=Homo sapier	0.00	2.34	2	1	1	1								1156	129.7	6.87
Q5JXM0	Putative uncharacterized protein DKFZp564C0482 OS=Homo	0.00	10.89	2	1	1	1								248	27.1	5.53
Q0EFA5	S protein OS=Homo sapiens GN=S PE=2 SV=1 - [Q0EFA5_I	0.00	2.34	6	1	1	1								512	49.9	8.13
Q8NGB0	Seven transmembrane helix receptor OS=Homo sapiens GN	0.00	0.61	1	1	1	1								1464	156.4	10.95
K7EL96	Perilipin-3 (Fragment) OS=Homo sapiens GN=PLIN3 PE=4 S	0.00	8.67	4	1	1	1	0.987	0.473	0.985	1.390	0.820	0.968	0.625	173	18.1	5.34
Q9H369	PRO1633 OS=Homo sapiens PE=2 SV=1 - [Q9H369_HUMAI	0.00	8.20	3	1	1	2								183	19.5	6.02
Q15164	Polyadenylate binding protein II (Fragment) OS=Homo sapi	0.00	7.25	10	1	1	1	1.575	0.954	1.540	1.646	1.458	1.722	1.291	193	22.0	8.95
Q96BA4	NUCB1 protein OS=Homo sapiens PE=2 SV=1 - [Q96BA4_H	0.00	7.39	6	1	1	1								176	20.5	5.11
